# Biotransformation Dynamics and Products of Cyanobacterial Secondary Metabolites in Surface Waters

**DOI:** 10.1021/acs.est.5c09247

**Published:** 2025-09-20

**Authors:** Xuejian Wang, Andrea Ingold, Elisabeth M.-L. Janssen

**Affiliations:** † Swiss Federal Institute of Aquatic Science and Technology, 28499 (EAWAG), Dübendorf 8600, Switzerland

**Keywords:** microcystins, anabaenopeptins, transformation products, reaction pathways, harmful bloom

## Abstract

Cyanobacteria produce toxic and bioactive secondary metabolites, posing risks to ecosystems and human health, yet their transformation pathways in surface waters remain unclear. We assessed biotransformation for 40 cyanopeptides including microcystins, anabaenopeptins and cyanopeptolins in surface waters and *in situ* enriched biofilm suspensions. In surface waters, most cyanopeptides did not degrade significantly over the course of 7 days. A wide range of biodegradability across cyanopeptides was apparent in biofilm suspensions from three rivers. Increasing the biofilm density shortened the lag time and increased initial removal of cyanopeptides. Increasing the initial cyanopeptide concentration lengthened the lag time and decreased their initial removal, supporting inhibitory effects of cyanopeptides toward enzymes involved in their own transformation. Transformation kinetics and product analysis demonstrated a structure–reactivity relationship across and within cyanopeptide classes. Anabaenopeptins were hydrolyzed at the C-terminus when arginine, tyrosine and (iso)­leucine were present, but not when phenylalanine or tryptophan was present. Microcystins showed tetrapeptide formation when adda linked to arginine but not when it linked to alanine, leucine, or tyrosine. Oxidation of tyrosine and deamination of arginine residues showed an interdependence across cyanopeptide classes. These novel insights into biotransformation products and pathways of a wide range of cyanopeptides facilitate assessment of exposure scenarios in surface waters and inform about kinetics and product formation in biological water treatment.

## Graphical abstract

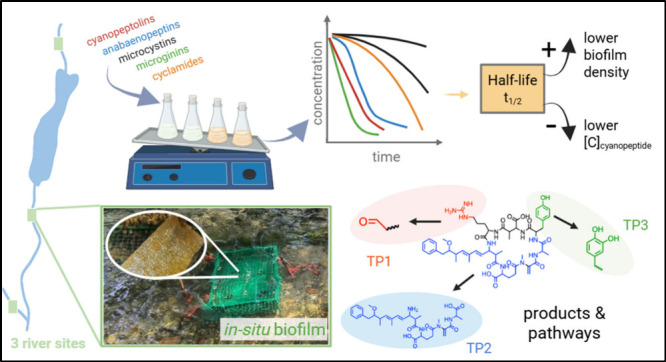

## Introduction

Cyanobacteria produce microcystins (MCs), cylindrospermopsins, anatoxins and saxitoxins, which receive special attention as recognized toxins by the World Health Organization (WHO).^1,2^ Beyond the WHO toxins, many bioactive secondary metabolites are coproduced by bloom-forming cyanobacteria and approximately 65% are peptide-based compounds, called cyanopeptides herein.^3–5^ Cyanopeptides can be grouped based on structural similarities including microcystins, anabaenopeptins, cyanopeptolins, or microginins with 329, 126, 288, and 122 respective variants known to date.^3,6,7^ Recent studies showed that other cyanopeptide classes were also detected in similar frequency and concentration levels as microcystins in both surface waters and intake of drinking water plants.^8–13^ Cyanopeptides are frequently reported to inhibit enzymes. For example, anabaenopeptins can inhibit phosphatases and carboxypeptidases.^14,15^ Cyanopeptolins can inhibit serine proteases,^12^ and microginins can inhibit zinc metalloproteases.^12,16^ Thus, exploring the persistence of these cyanopeptides for further risk assessment is of great interest. At the same time, the potential effects of their ability to inhibit various enzymes on their own transformation in surface waters remain to be explored.

While environmental decay process of only a few microcystins (e.g., MC-LR, MC-RR and MC-LF) have been examined,^17–20^ the environmental fate of other variants and coproduced cyanopeptides remain even more uncertain. Sunlight-driven photochemical transformation and biotransformation are two main transformation processes for cyanopeptides in surface waters. Sunlight driven processes can contribute to the environmental fate of cyanopeptides by direct photolysis and sensitized photochemical process including hydroxyl radicals and excited triplet states of dissolved organic matter.^21^ Photochemical half-lives can vary from hours to days across microcystins, cyanopeptolins and anabaenopeptins with structure–reactivity relationships favoring photolabile residues such as methionine and tyrosine.^22,23^

In the absence of sunlight, biotransformation can play a significant role in facilitating the transformation of cyanopeptides in surface waters. Previous studies investigated the biodegradability by some heterotrophic bacteria toward selected microcystins, nodularins and cylindrospermopsins.^24–29^ Half-lives are reported for microcystins ranging from days to weeks across various surface waters^30^ and enhanced degradation kinetics in biofilm enrichments.^31^ A recent review summarized tentative biotransformation products (TPs) of microcystins (MC-LR, MC-RR and MC-LF).^32^ The MC-LR enzymatic pathway has been best characterized initiated by hydrolysis of the adda-arginine-bound, e.g., by microcystinase, and further transformed into linear peptides and individual building blocks.^32–36^ Linearization and dehydration has also been suggested for microcystins but careful analysis of the chromatographic retention time and MS/MS fragmentation is required for correct identification.^33^ To gain a more mechanistic understanding of structural dependencies of microcystin biotransformation, a wider comparison across different variants and their TP formation is required. To date, no comprehensive analysis of biotransformation kinetics and TP formation beyond these few microcystins and none for other coproduced cyanopeptides such as anabaenopeptins, cyanopeptolins, cyclamides, or microginins is available.

Here, we studied the biotransformation dynamics of 40 cyanopeptides that were extracted from different monocultured cyanobacterial strains including microcystins, anabaenopeptins, cyanopeptolins, and microginins. We compared transformation dynamics across different surface waters and *in situ* biofilm suspensions and examined the influence of biofilm density and initial cyanopeptides concentration. We further identified biotransformation pathways and products that have not been reported to date for cyanopeptides, which allow the proposed general transformation reactions for key residues. Overall, our study provides novel insight of the environmental half-lives of cyanopeptides that are relevant for exposure scenarios in risk assessment and informs about the biotransformation process relevant to surface waters and biological water treatment.

## Materials and Methods

### Cyanobacterial Cultures and Metabolite Extraction

Nine cyanobacterial strains were used to obtain their secondary metabolites for the biotransformation study. *Microcystis aeruginosa* (PCC7806), *Dolichospermum flos aquae* (NIVA-CYA 269/6), *Planktothrix rubescence* (K-0576), *Microcystis aeruginosa* (UV006), *Microcystis panniformis* (MIRS-04), *Microcystis aeruginosa* (NPDC-01), as well as *Microcystis* G2011, *Microcystis* G2020 and *Planktothrix* G2020 isolated from Lake Greifensee in Switzerland.^13,37^ For details about origin and culture conditions see SI-1: Text S1.

Cyanobacterial secondary metabolites were extracted from lyophilized biomass using an adapted method that was previously reported.^22^ Therefore, 4 mL of methanol/water (70%/30% v/v) was added to 100 mg of lyophilized cyanobacterial biomass, vortexed, and incubated under sonication (VWR, Ultrasonic cleaner USC-THD, level 6, 15 min at 15 °C). Biomass was separated from supernatant by centrifugation (rcf of 3000 g at 10 °C for 15 min, Megafuge 1.0R), and the supernatant was transferred to a glass bottle. The same extraction was repeated one more time with the same biomass, and the supernatants were combined to yield the biomass extract. The extracts were diluted 30-fold with nanopure water and purified by solid phase extraction (SPE). For the SPE (12-fold vacuum extraction box, Visiprep, 12 ports, Sigma-Aldrich), the SPE cartridge (Oasis HLB 6 cc, 200 mg) was first conditioned with methanol and water (9 mL each) before loading the diluted extracts at a flow rate of 4–5 mL per minute. The cartridge was washed with 9 mL of nanopure water followed by 9 mL of methanol/water (20%/80% v/v) and eluted with 9 mL of methanol/water (85%/15% v/v) at a flow rate of 1–2 mL per minute. The eluted fraction was concentrated to 300 μL by vacuum-assisted evaporation (Syncore Analyst R-12, BÜCHI Labortechnik AG, 40 °C, 120 rpm, 20 mbar) and adjusted gravimetrically to 1.0 mL in ethanol. The final extract was stored at −20 °C until use.

### Biotransformation Experiments

Biotransformation experiments were performed in lake water and three river waters as well as with an *in situ* grown biofilm of these rivers. The experiment procedure was adapted from the OECD 309 guideline: Aerobic Mineralisation in Surface Water-Simulation Biodegradation Test, and previously reported methods.^38–40^

#### In-Situ Biofilms

Periphyton biofilms were grown on glass slides supplied with water from three streams in Switzerland: River Chriesbach, River Glatt, and River Mönschaltorfer Aa (MA). River Mönschaltorfer Aa (47.3516°N 8.6888°E) is upstream of lake Greifensee, a meso-eutrophic lake with mild cyanobacterial blooms.^13,41^ River Glatt (47.5740°N 8.4709°E) is downstream of lake Greifensee and River Chriesbach (47.40278°N, 8.60361°E) does not connect to lake Greifensee or other cyanobacterial impacted upstream sites. For River Chriesbach, biofilms were grown in flow-channels supplied with a bypass from the river and operated at a constant flow rate of 0.1 m/s under a light/dark cycle of 12:12 h.^40^ For River Glatt and River MA, glass slides (35.5 × 13.0 cm) were fixed vertically in perforated plastic boxes (two boxes for each river).^42^ See images of the setup in SI-1: Figure S1. The established biofilm material was removed from the glass after 4–6 weeks, suspended in streamwater, filtered (0.95 μm nylon net) to remove larger periphyton agglomerations and small organisms, and incubated in a glass bottle on a shaker (Edmund Bühler GmbH, Germany) for 0.5–2.5 days.

#### Experimental Setup

Five experiments were conducted with different surface waters, biofilm suspensions, and extracts from different cyanobacterial biomass. With the extract mixture of *M. aeruginosa* PCC7806, *D. flos aquae* NIVA-CYA 269/6, *P. rubescence* K-0576 and *M. aeruginosa* UV006, we evaluated the biotransformation of cyanobacterial metabolites: (1) comparing Lake Greifensee surface water, river water and biofilm suspensions from all three rivers; (2) for a dilution series of the initial cyanobacterial metabolite concentrations in biofilm suspensions from River Chriesbach; and (3) for a dilution series of the biofilm suspension from River Chriesbach. We further conducted additional experiments in Chriesbach biofilm suspensions with metabolite mixtures extracted from (4) *M. panniformis* MIRS-04 and *M. aeruginosa* NPDC-01 and (5) three Greifensee isolates (*M*. G2011, *M*. G2020 and *P.* G2020). An overview of the experiments is provided in SI-1: Table S1. Besides the active setup with surface waters and biofilm suspensions, abiotic controls include nanopure water, autoclaved surface waters, and autoclaved biofilm suspensions (autoclaving at 120 °C, two rounds for biofilm suspensions). For each condition, triplicates were prepared in glass vials that contained the respective matrix (nanopure water, surface water, biofilm suspension, or abiotic equivalent) and the respective metabolite mixture as well as benzoic acid (3 mM) as a readily biodegradable reference substance. Vials were covered with sterilized cotton plugs and placed on a vertical shaker with a UV-light filter (226, LEE filters, Hampshire, UK) to exclude direct photochemical transformation. Each vial was subsampled (300 μL) over the course of 4–7 days (SI-1: Table S1) and samples were immediately stored in glass vials at −20 °C until further analysis.

### Cyanobacterial Secondary Metabolite Analysis

Samples from the biotransformation experiment were thawed and centrifuged at 10621 rcf, 4 °C for 10 min (Centrifuge 5427R, Eppendorf, Germany), and the supernatants were transferred into analysis vials. All samples were analyzed within 24 h after thawing. The analysis was performed by high-performance liquid chromatography (HPLC; Dionex UltiMate3000 RS pump, Thermo Fisher Scientific) coupled with high-resolution tandem mass spectrometry (HRMS/MS, Fusion Lumos, Thermo Fisher Scientific). Chromatographic separation was carried out on a Kinetex C18 column (2.6 μm, 2.1 × 100 mm, Phenomenex, precolumn VanGuard Cartridge, Waters). The mobile phases consisted of (A) nanopure water and (B) methanol, both acidified with formic acid (0.1%). Binary gradient elution was carried out at a flow rate of 0.255 mL/min, increasing eluent B steadily from 20% to 70% between 0 and 30 min, from 70% to 100% between 30 and 31.4 min, and keeping at 100% between 31.4 and 38.5 min, then decreasing from 100% to 20% between 38.5 and 40 min, and keeping at 20% until 45 min. The injection volume was 100 μL. HRMS/MS used electrospray ionization (ESI) with 320 °C capillary temperature, 3.5 kV electrospray voltage, 275 °C vaporizer temperature, positive ionization mode, full scan from 120 to 1200 *m*/*z* with a nominal resolving power of 120000 at *m*/*z* 200, 25% automated gain control (AGC) target, and 50 ms maximal injection time. Data-dependent high-resolution product ion spectra were obtained by normalized collision energies for HCD of 15%, 30% and 45% at a resolving power of 15000 at 200 *m*/*z* isolation window, triggering data-dependent MS/MS acquisition using the public database CyanoMetDB.^7,43^

Data analysis and peak area extraction were performed with Skyline 20.1 (MacCoss Lab Software), as previously reported.^23^ Targeted analysis was performed for compounds where a reference standard or bioreagent was available (SI-1: Table S2). The stock solution of anabaenopeptin A and anabaenopeptin B were quantified by their molar extinction coefficients ε_278_ = 4190 M^–1^ cm^–1^ and ε_278_ = 2300 M^–1^ cm^–1^, respectively, in methanol,^44^ and the criteria for identification were based on exact mass (<5 ppm mass error), accurate isotopic pattern of the precursor ion (Skyline idotP > 0.95 considering top three isotopes), and match of retention time and MS^2^ spectra of reference materials. In addition, suspect screening analysis was conducted for all compounds for which no reference material was available. First, full-scan (MS^1^) spectra were screened for exact masses (<5 ppm mass error) and accurate isotopic patterns of the precursor ion (Skyline idotP > 0.95) for metabolites reported in the cyanobacterial suspect list CyanoMetDB.^7,43^ MS^2^ spectra corresponding to tentative candidates were manually annotated and supported where possible, by in-silico fragmentation predictions (MetFrag Web with CyanoMetDB Version02 2023 database).^43^ Predicted and measured spectra were manually evaluated and the level of confidence was assessed.^45^ Metabolites were classified as a tentative candidate (Level 3) based on exact mass (<5 ppm mass error), accurate isotopic pattern (Skyline idotP value >0.95), and evidence from fragmentation data; a probable structure (Level 2b) based on additional, comprehensive MS^2^ fragmentation information that helped to confirm the connectivity of molecular substructures; or a confirmed structure (Level 1) when these parameters were in agreement with available reference standards or bioreagents. Documentation of the annotations of metabolites have been previously published^13,46–48^ and additional annotated metabolites are presented in SI-2. The peak areas of selected ion chromatograms were extracted for all metabolites classified as level 1 or 2b. Analysis of benzoic acid is detailed in SI-1, Text S2.

### Identification of Biotransformation Products and Kinetics

Besides consulting the literature for reported TPs,^32^ in-silico prediction tools were implemented including EnviPath and BioTransformer3.0. EnviPath is a database and prediction system for the microbial biotransformation of organic environmental contaminants from sludge and soil.^49^ BioTransformer 3.0 is a software tool to predict small molecule metabolism in mammals, gut microbiota, as well as soil/aquatic microbiota.^50^ The prediction can be conducted from the SMILES of parent compounds and the output includes possible transformation pathways and TPs including molecular formulas and SMILES.^51^ For each cyanopeptide, a list of possible TPs was collated, considering individual transformation reactions at specific moieties and their various combined occurrence. All identified TPs, their corresponding precursors and tentative transformation pathways are listed in SI-2 (tab: “TransformationProductList”). To identify TPs in experimental samples, suspect screening of the collated list of possible TPs was applied. First, the workflow in Skyline, MS^2^ annotation, and assignment of the confidence level was conducted based on above-mentioned criteria. Any TPs present in abiotic controls were excluded from further analysis. As no reference standard or bioreagent was available for the TPs, we only report TPs with confidence level 2b and level 3. The peak areas of selected ion chromatograms of TPs were extracted for kinetic analysis.

Transformation kinetics of cyanopeptides were followed by calculating the relative abundance for all subsampling time points as *A*_t_/*A*_0_, with measured areas (*A*) at time *t* compared to the initial area at time 0. In our experiment, a lag phase was regularly observed, i.e., time while the concentration initially remained stable during exposure before a significant decrease was observed. Thus, a shoulder-log linear model was used as follows:^52^

$$\frac{A_{t}}{A_{0}}=e^{-kt}\left(\frac{e^{kS}}{1+\left(e^{kS}-1\right)e^{-kt}}\right)$$

where *t* is time (h), *S* (h) is the shoulder time, which we call lag time hereafter, and *k* (h^–1^) is the rate constant for the log linear portion of the decay curve. Nonlinear least-squares fitting was performed using the nlsLM function in *R* (2024.12.0 + 467), with parameter bounds set as follows: *k* was constrained between 10^–6^ and 10, *S* between 0 and 168 h (experiment duration), and the ratio of *A*_*t*_/*A*_0_, estimated from the average of triplicates at time 0 was restricted to 80–120% of the mean value. Data below limit of detection (LOD) are removed from fitting for compounds with reference standards; for compounds without a reference standard, the LOD of a structurally similar standards were used (SI-1: Text S3). If the 95% confidence interval for *S* included zero, indicating that the shoulder was not statistically significant and no lag phase was apparent, the model automatically followed a pseudo-first-order decay model for such data. When the regression coefficient was low (*R* ^2^ < 0.6), a biphasic model was applied were applicable (3 out of 71 cases, SI-1: Tables S4 and S5). When less than three time points were available (i.e., above LOD) or no statistically significant decrease was observable (*t* test to verify whether the relative area units (*A*_*t*_/*A*_0_) at time *t* were not significantly different from the initial values, ρ > 0.1) no model was fitted. The details of model fitting evaluation are shown in SI-1: Text S4.

## Results and Discussion

First, we assessed the biodegradability of cyanobacterial metabolites across different surface waters including lake Greifensee water and three rivers for microcystins (*n* = 12), anabaenopeptins (*n* = 8), cyanopeptolins (*n* = 12), microginins (*n* = 2), cyclamides (*n* = 4), planktocyclin, and oscillacyclin (SI-2). For 21 metabolites, we observed no significant biotransformation during incubation in surface waters for up to 7 days, with few exceptions detailed in the later discussion herein (SI-1: Figure S2). A highly biodegradable reference compound, benzoic acid, was degraded within hours of incubation, demonstrating general biological activity in these surface waters, and remained stable in abiotic controls (SI-1: Figure S3). The transformation of some microcystins was previously reported to be generally slow in ambient surface water with half-lives ranging from days to weeks.^30^ As the cyanobacterial metabolites tested were rather persistent in these surface waters, comprehensive analysis comparing their degradation kinetics and product formation required higher microbial activity or significantly longer incubation times. To avoid artifacts due to long incubation time, we further evaluated biotransformation in suspensions of in situ grown biofilms as enriched communities from the same river waters.

### Biotransformation Kinetics

We conducted three sets of experiments to compare transformation kinetics with respect to (a) location across different surface waters and their respective biofilm suspensions; (b) initial biofilm densities; and (c) initial concentration of cyanopeptides. Here we used the extracts of *M. aeruginosa* PCC7806, *D. flos aquae* NIVA-CYA 269/6, *P. rubescence* K-0576 and *M. aeruginosa* UV006 which allowed us to investigate 26 cyano-metabolites (SI-2). Three metabolites of the class of cyclamides (aerucyclamide C, aerucyclamide D and piricyclamide ILGEGEGWNYNP+prenyl), oscillacyclin and planktocyclin show statistically significant abiotic removal in the autoclaved controls (*t* test, *p* < 0.1 and *A*_t_/*A*_0_ > 0.7). Aerucyclamide D and planktocyclin carry a methionine residue which can be prone to abiotic oxidation. We excluded these compounds from further discussion of biotransformation hereafter (SI-1: Figure S4).

#### Comparison across Biofilms from Different Locations

We compared biotransformation with biofilm suspensions from three different rivers for the same metabolite mixtures and concentrations. Biotransformation kinetics of exemplary cyanobacterial metabolites are shown in Figure 1A. The concentration of cyanopeptolin 1020 and anabaenopeptin B decreased by 87% and 74% within 3 days, respectively, and were not detectable anymore at day 5. The microcystins MC-YR and MC-LA decreased by only 30% and 12% within 3 days, respectively, demonstrating significantly lower biodegradability. The shoulder-log fitting also demonstrates a longer lag time for MC-YR (4.2 days) and MC-LA (7.0 days) compared to anbaenopeptin B (2.3 days) and cyanopeptolin 1020 (1.6 days). Kinetics for all 21 metabolites are presented in SI-1: Figure S2. Data in Figure 1B show the comparison of the removal within 3 days upon incubation with biofilm from River Chriesbach relative to incubation with biofilms from River Glatt and River Mönschaltorfer Aa to compare behavior across all metabolites. In Chriesbach biofilm suspensions, most cyanopeptolins (cyanopeptolin A, cyanopeptolin B, cyanopeptolin C, cyanopeptolin D, and cyanopeptolin 1020) as well as anabaenopeptin B and anbaenopeptin F show more than 50% removal on day 3. Cyanopeptolin 963A, anabaenopeptin A and oscillamide Y were removed by 32%–35%. Removal for microcystins was more variable, decreasing from [d-Asp^3^]­MC-(H4)­YR (86%), [d-Asp^3^, (*E*)-Dhb^7^]­MC-RR (46%), MC-HtyR (31%), MC-YR (30%), MC-LR (18%) to the rather stable congeners MC-LA, [d-Asp^3^]­MC-LA, MC-LL and MC-LAba (<13%). Aerucyclamide A was the only representative of cyclamides that was stable in abiotic controls and the 3-day removal was 20%. Tabulated kinetic data (lag time, half-life, % removal) is presented in SI-1: Table S4.

**Figure 1:**
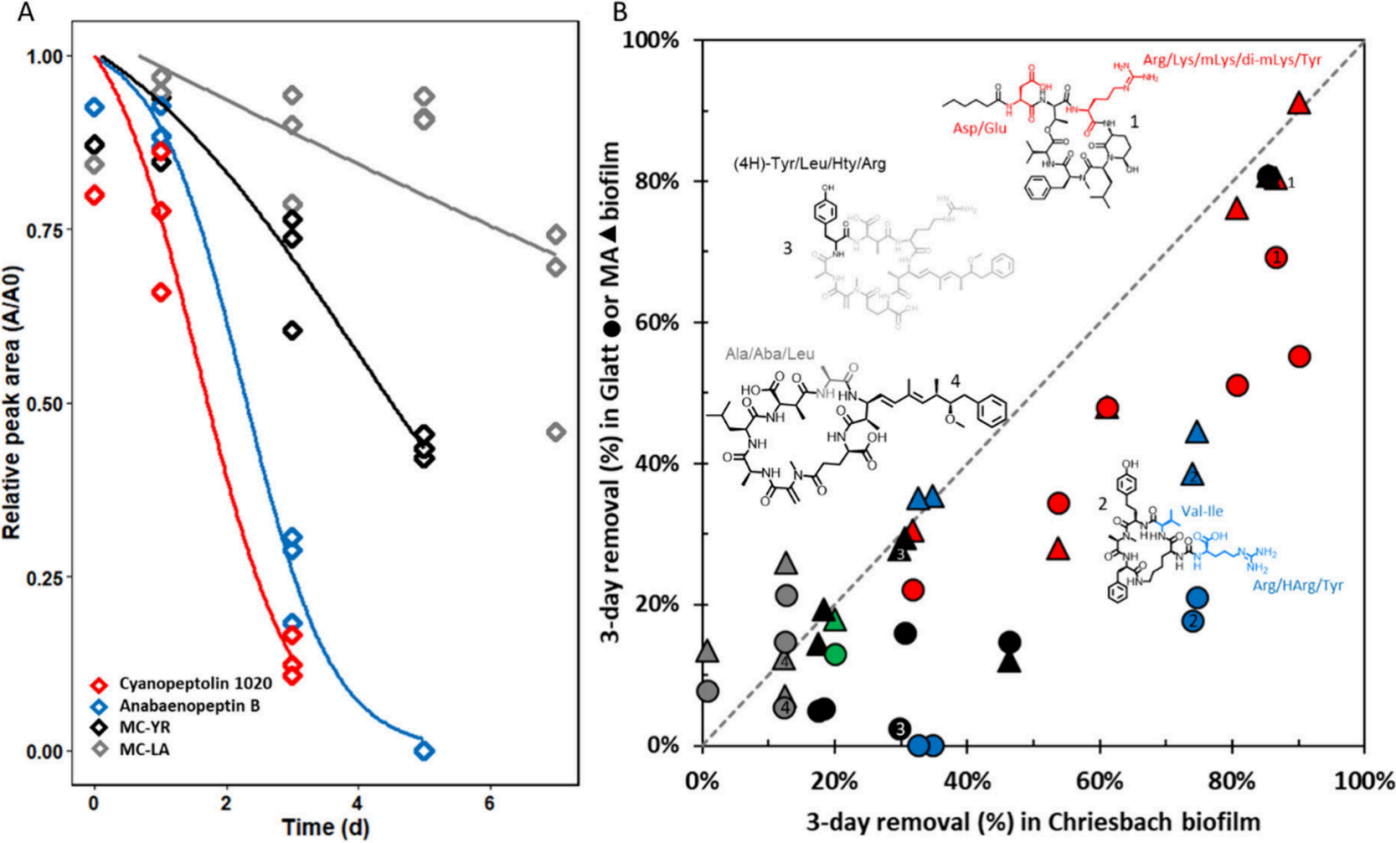
(A) Biotransformation kinetics with shoulder-log fitting in River Chriesbach biofilm suspensions of four cyanobacterial metabolites: Cyanopeptolin 1020 (red, 1), Anabaenopeptin B (blue, 2), MC-YR (black, 3) and Microcystin-LA (gray, 4) expressed as peak area units (*A*) normalized to initial values (*A*_0_) over incubation time in days showing triplicate samples. (B) Comparing percent removal in River Chriesbach biofilm (on *x*-axis) to removal in River Glatt biofilm (on *y*-axis, triangles) and River Mönschaltorfer Aa (MA) biofilm (circles) for 21 cyanobacterial metabolites marked according to their compound class of cyanopeptolins (red), anabaenopeptins (blue), microcystins (black and gray), and Aerucyclamide A (green). Representative structures are shown, and highlighted residues represent the most variable part for the compound class. Tabulated data are presented in SI-1: Table S3 and molecule structures are in SI-2.

Cyanopeptolins (red) showed overall high removal, anabaenopeptins (blue) showed intermediate removal, while microcystins showed slower (black) or no significant removal (gray), with the exception of [d-Asp^3^]­MC-(H4)­YR. The range of biodegradability was consistent across biofilms from the three rivers (i.e., linear trend in Figure 1B). Yet, the absolute removal was generally lower in River Mönschaltorfer Aa and lowest in River Glatt compared to River Chriesbach biofilm. To evaluate the overall bioactivity of biofilm suspensions, we compared the degradation of the readily bioavailable reference compound benzoic acids. The fastest removal of benzoic acid was observed in Chriesbach biofilm suspensions with 72% and 100% after 8 and 24 h, respectively, which was reproducible when repinning benzoic acid on day 2 of incubation (Figure S3). For River Mönschaltorfer Aa removal was significantly slower with 41% (8 h) and 84% (24 h) and indeed the slowest in River Glatt (3% and 75%) confirming lowest bioactivity overall, not only toward cyanopeptides, which was in line with location specific biodegradabilities.

#### Effects of Biofilm Density

Data in Figure 2A–C show the effect on transformation kinetics when diluting the biofilm suspension 10-fold (10%) and 100-fold (1%). As expected, the removal in the first 24 h (1 day) decreased with decreasing biofilm density for all metabolites that showed detectable transformation (examples in Figure 2A, others in Figure S5). In our study, metabolites generally showed a lag time before transformation was detectable. For cyanopeptolin 1020 (red) the initial removal at 1 day decreased from 91% (100% biofilm) to 31% (10% biofilm) and there was no significant removal in 1% biofilm and river water. Cyanopeptolin 1020 also showed a significant extended lag time with reduced biofilm density, from 6.7 h (100% biofilm) to 2.5 days (10% biofilm), 4.1 days (1% biofilm), and 5.4 days in ambient river water (Figure 2B. The half-lives, considering lag time and transformation rate *k*, also increased accordingly (Figure 2C). For anabaenopeptin B (blue), similar observations were made for 100% and 10% biofilm densities and at 1% density and ambient river water (patterned bars, Figure 2B,C) the kinetics becomes too slow for accurate estimates of lag time and half-lives and significant decay was detectable only at the final day, 7 days (*t* test *p* < 0.1). For the slower degrading microcystins, estimates were only possible at higher biofilm densities when overall activity was high (e.g., MC-YR, in black). MC-LA was stable in all biofilm densities. The lag time, half-lives, and initial removal for all 21 metabolites cyanopeptides are presented in SI-1: Table S4. For benzoic acid, a reference compound for overall microbial activity, significant removal occurred in all three biofilm densities after 1 day (97% removal at 100% density, 77% at 10% density, and 62% at 1% density, SI-1: Figure S3). Overall, diluting the initial biofilm suspension decreased initial removal and extended the lag time for 17 out of 21 metabolites that proved labile toward biotransformation (SI-1: Table S4).

**Figure 2:**
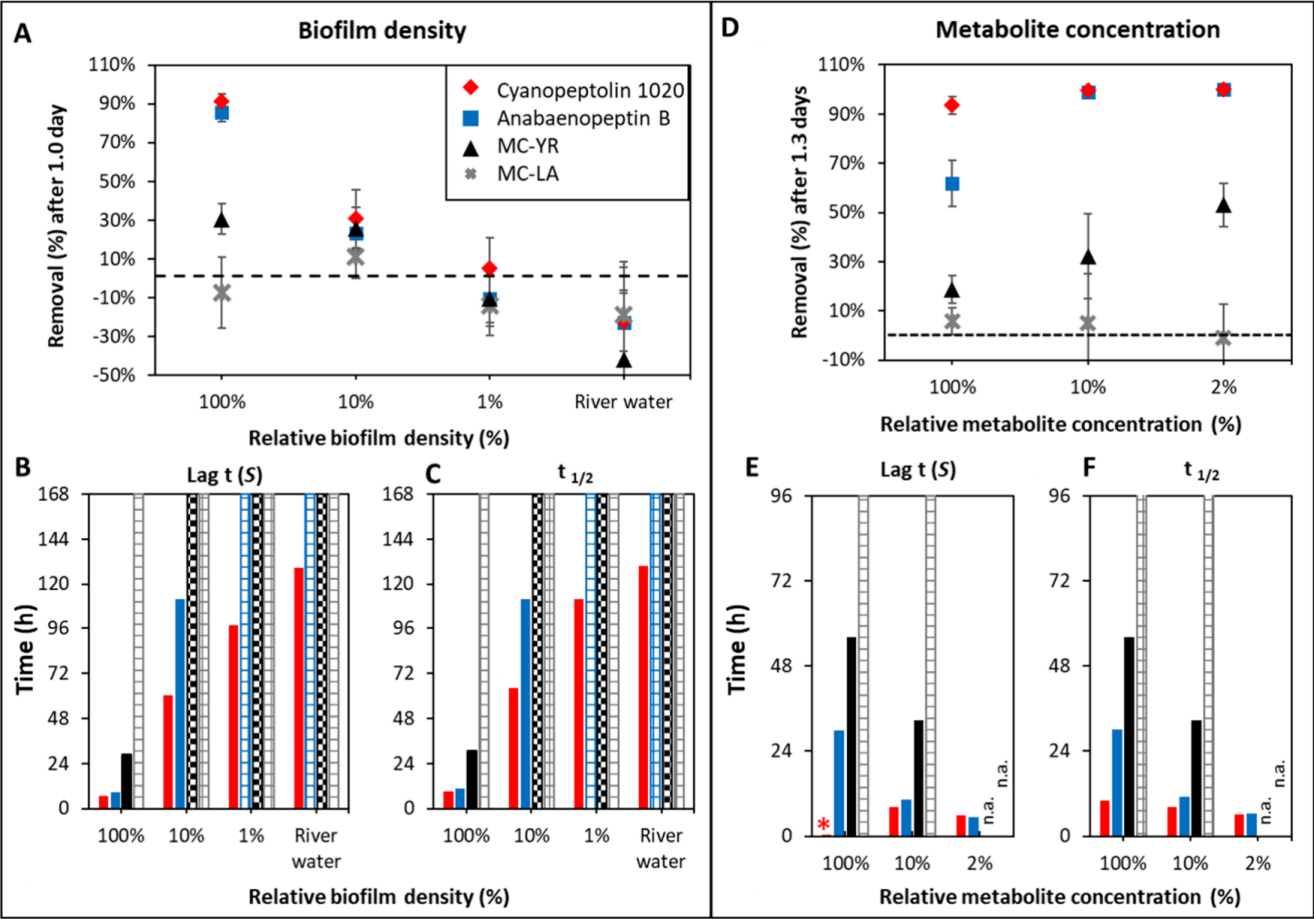
Initial metabolite removal after 1 day, lag times (Lag *t* (S)), and half-lives (*t*_1/2_) were calculated from biotransformation kinetics for incubations in three biofilm densities (100, 10, 1%) and ambient river water (panels A, B, and C) and for three initial cyanobacterial metabolite concentrations (100, 10, 2%) (panels D, E and F) for representative metabolites: cyanopeptolin 1020 (red), anabaenopeptin B (blue), MC-YR (black), and MC-LA (gray). The dashed lines mark zero percent removal in panels A and D. Hashed-bars for lag times and half-lives in panels B and C indicate that removal was not observed (or only slight removal on the final day) and hence values exceeded the experimental exposure time. Lag time and half-lives denoted by “n.a.” could not be assessed due to low regression coefficients (*R* ^2^ < 0.6) especially at low metabolite concentrations in panels E and F. The asterisk, *, in panel E represents a lag time of 0.4 h and the bar is too low to be seen properly.

The dilution of biofilm suspensions proves that with a lower concentration of biofilm, i.e., lower microbial and enzymatic density, a longer lag time and slower transformation rate occurred. Tests in diluted biofilm suspensions approached biotransformation conditions in ambient surface waters. Only cyanopeptolin 1020 and [d-Asp^3^]­MC-(H4)­YR (SI-1: Figure S5) showed detectable degradation in ambient river water. Similar to cyanopeptolin 1020, half-lives increased for [d-Asp^3^]­MC-(H4)­YR from 7.6 h in 100% biofilm density to 2.6, 5.0, and 7.1 days in 10% and 1% suspensions and in river water. For both compounds, we observed a log–linear relationship between half-lives and biofilm densities, and we used this regression to estimate that ambient river water showed biotransformation kinetics equivalent of a 0.1–0.5% biofilm suspension (Figure S6). For oscillamide Y and anabaenopeptin A, we could measure half-lives in all three biofilm densities, but kinetics were too slow in ambient river water. We used the regression of half-lives and biofilm densities and the biofilm equivalent for river water, 0.1–0.5%, to estimate expected river water half-lives ranging from 7.2 to 8.0 days for oscillamide Y and 8.6–10.6 days for anabaenopeptin A. Here, we demonstrated the ability to use kinetics comfortably measured in enriched biofilm suspensions to approximate surface water half-lives that are otherwise too slow to capture. However, biotransformation of most metabolites was too slow even in the 1% biofilm density and no regression could be fitted. To enable such assessment for more metabolites, dilution of biofilm suspensions should be selected to ensure that at least a set of three dilutions can be used to estimate the river water conditions.

#### Effect of Initial Metabolite Concentration

As microcystins, cyanopeptolins, and anabaenopeptins are known to be bioactive and have inhibiting effects on various enzymes, higher concentrations of these mixture could have had inhibiting effects on enzyme activities in the biofilm suspensions relevant for cyanopeptide biotransformation itself. For 10 out of 21 metabolites, reference standards or bioreagents were available, and concentrations added to the experiments were in the high μg/L to low mg/L range for individual compounds. Data in Figure 2D–F show the effect of reducing the initial concentration by 10-fold (10%) and 50-fold (2%) compared to the initial experiments (denoted as 100%). The initial removal at 1.3 days (Figure 2D), lag times (Figure 2E), and half-lives (Figure 2F) are shown for the four exemplary compounds. For anabaenopeptin B (blue), the removal increased from 62% to nearly 100% when decreasing the initial metabolite concentrations. The lag time reduced from 29.9 h (100% metabolite concentration) to 10.3 h (10%) and 6.7 h (2%); also, the half-lives decreased from 30.0 to 11.2 and 6.2 h. For cyanopeptolin 1020 initial removal slowed down slightly with metabolite concentrations leading to the longest half-lives of 9.9 h at 100% initial concentration compared to 8.3 and 6.2 h when concentrations decreased to 10% and 2%, respectively. For MC-YR the removal increased from 19% to 32% when lowering the initial metabolite concentration by 10-fold and the half-live decreased from 2.3 days to 1.4 days. With the lowest initial metabolite concentrations, the model fit did not always allow us to estimate lag times and half-lives as concentrations of less abundant metabolites quickly decreased below the limit of detection, resulting in not enough data points for the model fitting. MC-LA showed high stability in all metabolite concentrations during the experiment period (removal around 0%) and no decay kinetics were estimated (hashed bars, n.a., Figure 2E,F). Overall, diluting the initial metabolite concentration increased the initial removal and decreased the lag time, if present, for 17 out of 21 metabolites that show biodegradability (SI-1: Table S4). On the other hand, the reference compound for overall microbial activity, benzoic acid, showed consistent removal rates of 94–100% for all metabolite dilutions (100%, 10% and 2%) within 4 h. Consequently, we hypothesize that the higher metabolite concentrations did not inhibit general microbial processes to a significant extent but affected mostly extracellular enzymatic processes, likely due to their role as enzyme inhibitors.

A similar lag phase for biotransformation was previously described for MC-LR, dmMC-LR and MC-LF,^30,53–56^ and it was hypothesized that the initial exposure levels of microcystins may have influenced biotransformation.^55,57^ Another study regarding the biotransformation of trace organic contaminants with active sludge pointed out that some contaminants, which were removed via catabolic degradation or co-metabolism, would first trigger a biomass increase of specific degraders and authors hypothesized that the time needed for those degraders to grow may explains the lag phase before a significant removal can be observed.^58^ Here we demonstrate that higher cyanopeptide concentrations scale with slower biotransformation and longer lag times. However, it remains to be investigated to what extent each cyanopeptide or cyanopeptide class contributed to the observed effects.

### Biotransformation Products and Pathways

Biotransformation products and pathways were explored for an extended range of cyanopeptides (SI-1: Table S1, experiments 3–5). By using in-silico prediction tools and combining possible transformation on different building blocks within each cyanopeptide, we were able to screen for a wide range of possible TPs. For those TPs identified and confirmed by MS^2^ annotation (SI-2), the transformation kinetics were extracted, and plausible transformation pathways were explored. No suspected transformation products could be identified for the 12 cyanopeptolins and 2 microginins investigated (SI-1: Figure S7). For cyanopeptolins, an initial and fast hydrolysis of the ester bond is expected and subsequently fast hydrolysis of the resulting linear peptide, similar to that of microginins. Here, we will first demonstrate the main biotransformation pathways observed specifically for microcystins and anabaenopeptins; then we will point out common biotransformation pathways of individual building blocks across both metabolite classes. An overview of all identified TPs, their corresponding precursors and tentative transformation pathways are listed in SI-2 (tab: “TransformationProductList”).

#### Microcystins

One biotransformation pathway for microcystins resulted in the formation of the tetrapeptide NH_2_-Adda-Glu-Mdha-Ala-OH. The formation of this tetrapeptide can stem from different congeners and has previously been reported, for example, for MC-LR, [Dha^7^]­MC-LR, MC-RR and MC-LF.^32,34^ Data in Figure 3 show the formation of the tetrapeptide (TP1) from MC-YR with an increasing trend during the 4-day incubation to biofilm suspension. Considering the biodegradable microcystins and their amino acid residues included in this study, MC-LR, [d-Asp^3^]­MC-LR, MC-YR, MC-HtyR and [d-Asp^3^]­MC-(H4)­R are all possible precursors of the same tetrapeptide, which influences formation kinetics when precursors have different half-lives. Overall, we observed that all microcystins with the adda moiety connecting to arginine were biodegradable: MC-LR, [d-Asp^3^]­MC-LR, MC-YR, MC-HtyR, [d-Asp^3^, (E)-Dhb^7^]­MC-RR and [d-Asp^3^]­MC-(H4)­R. Microcystins with alanine, leucine or tyrosine connecting to adda remained rather stable: [d-Asp^3^]­MC-LA, MC-LAba, MC-LL, MC-LA and MC-LY (SI-1: Figures S5 and S7). Our observations suggest that peptidases that preferentially cleave peptide bonds at the carboxyl side of arginine contributed to the biotransformation of labile microcystins. Previously, also hydrolysis forming the tetrapeptide was tentatively identified when phenylalanine was in place of arginine in MC-LF.^53^ Previous studies reveal that once MC-LR is cleaved into tetrapeptide, the toxicity decreased significantly^35^ although non-negligible,^59^ suggesting lower environmental risks.

**Figure 3:**
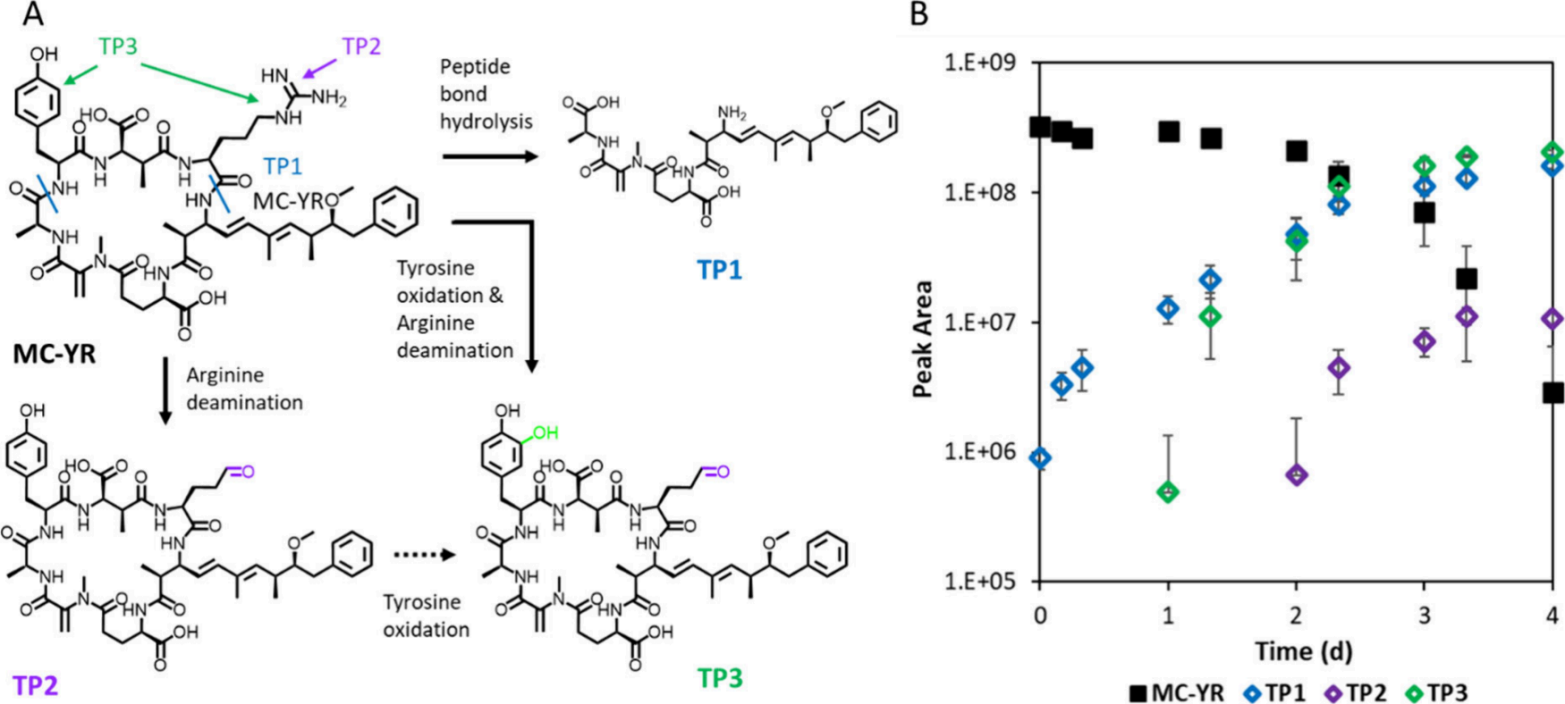
(A) Biotransformation products (TP1–TP3) of MC-YR and (B) kinetics of the parent and transformation products (TPs) upon exposure to River Chriesbach biofilm suspensions. Note that TP1 and TP2 can also be formed by other precursors (see the list in SI-2).

TP2 and TP3 will be further discussed in the subsequent section on tyrosine oxidation and arginine deamination.

#### Anabenopeptins

To the best of our knowledge, no TPs of anabaenopeptins were previously reported. In our study, biotransformation at the side chain of several anabaenopeptins was observed. Here, either the N–C bond linking the CO-moiety to the lysine R-group was hydrolyzed to form TP2 or the C–N bond between the CO-moiety and the C-terminal amino acid was oxidized to form TP1, as shown for Anabaenopeptin B in Figure 4. While TP2 is formed by hydrolysis of the quasi-peptide bond, TP1 is the oxidation product suggested by the biotransformation rule bt0243 proposed by Envipath. Bt0243 suggests oxidative removal of an aliphatic R-group from a secondary or tertiary urea or amide nitrogen.^60^ Anabaenopeptin A and anabaenopeptin J have a similar structure to anabaenopeptin B with tyrosine and isoleucine, respectively, instead of arginine as the C-terminal amino acid and both formed TP1 and TP2 in the same manner as anabenopeptin B. These TP1 and TP2 were further formed by anabaenopeptin F, oscillamide Y and anabaenopeptin 807, which carry the same C-terminal amino acids (arginine, tyrosine, isoleucine, respectively) and only differ in the cyclic part of the molecule when isoleucine replaces valine (SI-1: Figure S9). Peptidases are widely secreted by bacteria in natural environment;^61^ thus the hydrolysis of peptide bonds at the side chain of anabaenopeptins to form TP2 is plausible. The kinetics of the TPs reveal that they were formed seemingly simultaneously and subsequently decayed (Figure 4). However, three remaining anabaenopeptins, namely anabaenopeptin D, anabaenopeptin 871 and ferintoic acid B, with phenylalanine and tryptophan at the C-terminal end, did not show detectable biotransformation (SI-1: Figure S8). These observations suggest that peptidases contributed to the biotransformation of anabaenopeptins by preferentially cleaving peptide bonds at the C-terminus unless the amino acids carried an apolar aromatic R-group. As these initial TPs are not stable, we searched for TPs that result from the further hydrolysis of peptide bonds within the cyclic structure. As no linear peptide could be identified, we hypothesize that there may not be one major site for hydrolysis and various hydrolysis sides may be attacked simultaneously, resulting in a range of peptides that are challenging to detect. Anabaenopeptins are known peptidase inhibitors^14^ but the interaction of these TPs with the enzymatic function remains unknown. As the TPs themselves are biodegradable herein, their lifetime in surface waters shall not exceed that of the parent compounds.

**Figure 4:**
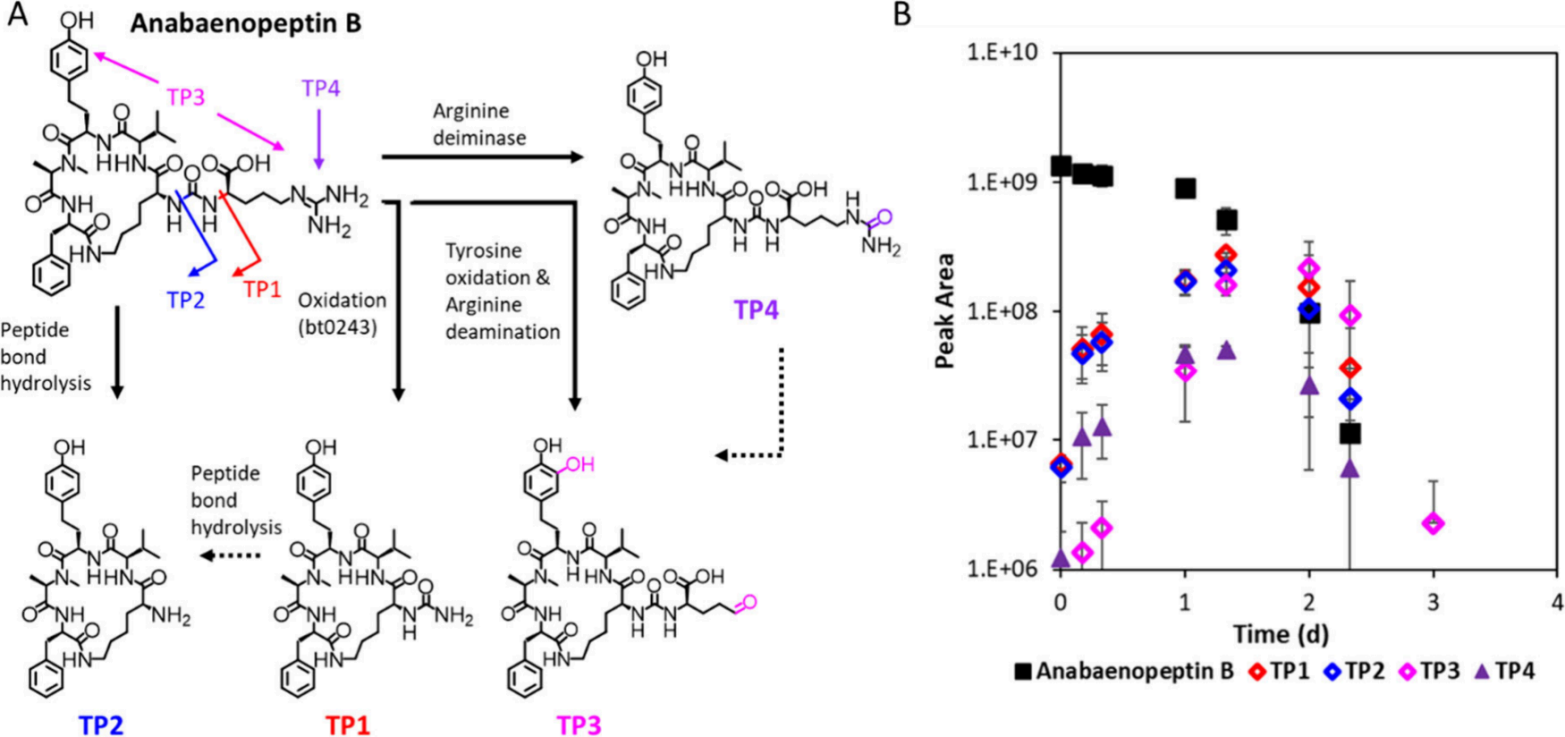
(A) Biotransformation products (TPs) of Anabaenopeptin B and (B) kinetics of the parent and transformation products (TPs) upon exposure to River Chriesbach biofilm suspensions. Note that TP1 and TP2 can also be formed by other precursors (see list in SI-2).

#### Tyrosine Oxidation

For some metabolites carrying tyrosine or homotyrosine moieties, oxidation to the diphenols was observed, as shown for MC-YR in Figure 3 (TP3) and for Anabaenopeptin B in Figure 4 (TP3). This oxidation of tyrosine was also observed for MC-HtyR and anabaenopeptin F, which also carry a tyrosine in the cyclic part of the structure and an arginine moiety in proximity (SI-1: Figures S9 and S10; SI-2). However, other anabaenopeptins carrying a tyrosine in their cyclic structure but without an arginine in proximity (at the C-terminal end) did not show formation of the diphenol including anabaenopeptin A, oscillamide Y, anabaenopeptin 871, anabaenopeptin 807, anabaenopeptin D, anabaenopeptin J, and ferintoic acid B. Similarly, MC-LY did not form a diphenol at the tyrosine and this microcystin does not contain an arginine moiety elsewhere in its structure compared to MC-YR and MC-HtyR. Previous studies reported that tyrosinases can catalyze the *ο*-hydroxylation of monophenols to the corresponding *ο*-diphenols and the subsequent two electron oxidation of *ο*-diphenols to the corresponding *ο*-quinones.^62–64^ The latter reaction was not detected herein. Our observations hint at the specificity that tyrosine oxidizing enzymes, e.g., tyrosinases, require an arginine in proximity. The detailed mechanistic understanding of this dependency remains to be elucidated beyond the presented study.

#### Arginine Deamination

The hypothesis that arginine plays a role in the oxidation of tyrosine is further supported by the proceeding or co-occurring deamination of the arginine side chain in these cyanopeptides. Deamination of arginine forms glutamate-5-semialdehyde, also called l-glutamate gamma semialdehyde or gamma-glutamyl semialdehyde, shown for MC-YR in Figure 3 (TP2 and TP3) and for anabaenopeptin B in Figure 4 (TP3). Deamination of the arginine side chain was also observed for MC-HtyR and anabaenopeptin F, for which tyrosine oxidation was observed, as well as for MC-LR and [d-Asp^3^,(*E*)­Dhb^7^]­MC-RR (SI-1: Figures S9–S11; SI-2). The glutamate-5-semialdehyde has been reported as a product not only of metal-catalyzed oxidation of arginine (or proline)^65^ but also of enzymatic reactions (Figure 5). The arginine deiminase pathways catalyzes arginine to citrulline (EC 3.5.3.6), while generating ATP, and is further transformed to ornithine by ornithine transcarbamylase (EC 2.1.3.3).^66^ The pathway is widely distributed among bacteria and often present a major mean of energy production.^67^ Ornithine aminotransferase (EC 2.6.1.13) can catalyze the reaction of ornithine to also yield glutamate-5-semialdehyde.^68–70^ Although the reaction by ornithine aminotransferase was reported in human tissue, the enzyme is also expressed in fungi.^70,71^ Inspired by these reported pathways, we further inspected possible intermediates and observed the citrulline intermediate for anabaenopeptin B (Figure 4, TP4). The kinetics of the citrulline intermediate (TP4) and the glutamate-5-semialdehyde intermediate, with additional tyrosine oxidation (TP3) are both formed seemingly simultaneously and subsequently decay.

**Figure 5:**
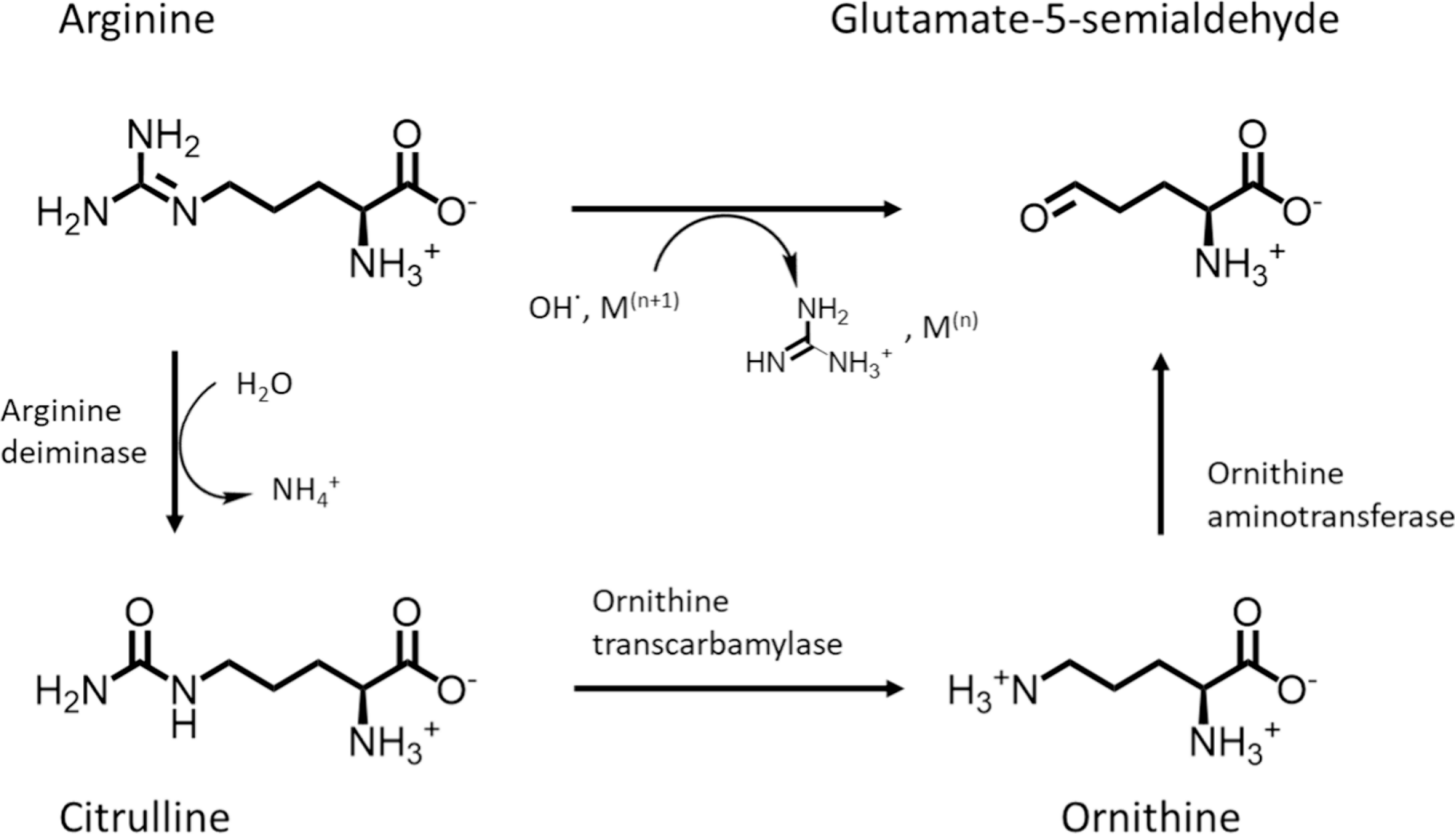
Reaction of arginine by deiminase to citrulline and further reaction with transcarbamylase to ornithine^66^ and with aminotransferase to glutamate-5-semialdehyde,^68–70^ which can also be formed directly via a metal catalyzed reaction.^65^

TPs of anabaenopeptins that result from tyrosine oxidation and arginine deamination show further decay in our experiment; thus, their lifetime in surface water is expected to be comparable to parent compounds. Except for the arginine deamination product of [d-Asp^3^, (E)­Dhb^7^]­MC-RR, TPs of MCs that result from tyrosine oxidation and arginine deamination do not show further decay in our experiment period, indicating longer lifetimes than the parent compounds and their potential toxicity has to be considered. For these reactions the ring of MCs and the adda moiety remains intact; thus, it is likely that TPs maintain toxicity. While the presence of arginine can lower the potency of MC cytotoxicity by varied interaction with transporters OATP1B1 and OATP1B3,^72^ MC-LR and MC-LA that differ in the side chain of one building block, present similar LD_50_ values for mice and similar IC_50_ values toward seine/threonine protein phosphatases.^73^ A modification of the side chain of one building block in MCs is likely to affect toxicity, but at the same time, it is not expected that the toxicity is eliminated, which requires further studies.

Overall, when anabaenopeptins and microcystins contained both residues, tyrosine and arginine, TPs with both tyrosine oxidation and arginine deamination were observed herein, which were not reported previously, while TPs from only deamination were observed for MC-YR (Figure 3, TP2), MC-LR and [d-Asp^3^, (*E*)­D-hb^7^]­MC-RR. TPs from only oxidation of tyrosine were not observed for any cyanobacterial metabolite. Kinetic analysis of MC-YR suggests that the glutamate-5-semialdehyde intermediate (TP2) was formed before TP3 with additional tyrosine oxidation, while the opposite was expected. Other than for the anabaenopeptin, these TPs were building up and not decaying, suggesting that peptidases were not able to form the microcystin tetrapeptide from these TPs as efficiently. One exception was the deamination product of [d-Asp^3^, (*E*)­D-hb^7^]­MC-RR which showed subsequent decay (SI-1: Figure S11).

### Implications

We demonstrate that biotransformation kinetics for a wide range of microcystins, anabaenopeptins, cyanopeptolins, and individual cyclamides and microginins vary widely within and across compound classes. Biotransformation in four tested surface waters was too slow to be captured during a 7-day exposure for most compounds but was apparent in concentrated *in situ* grown biofilm suspensions. A dilution series of biofilm suspensions confirmed that lag time shortened and transformation rates decreased with reduced biological densities, eventually approaching surface water conditions. Equivalent biofilm densities in river water could be estimated and allowed to approximate half-lives of cyanopeptides in surface waters that were too slow to experimentally derive. *In-situ* biofilm dilution series for biotransformation studies present a vital experimental alternative when low biological activity in surface waters hinders the assessment of transformation kinetics directly. However, a wider range of biofilm densities needs to be considered to capture varies degradation kinetics across target compounds. We further demonstrate that higher initial cyanopeptide concentrations increase the lag time and slowed down their overall removal. These observations support the widely reported effects of enzyme inhibition by cyanopeptides and further suggest that a mixture of cyanopeptide from bloom-forming cyanobacteria can inhibit those enzymes, required for their own degradation. Exposure with enhanced biofilm densities also enabled a detailed analysis of transformation products and pathways. A comprehensive transformation product analysis allowed us to identify novel biotransformation products and structure–reactivity relationships within and across compound classes. Microcystin variants with the adda moiety linked to arginine were labile and formed a characteristic tetrapeptide while those linking adda to alanine, leucine, or tyrosine were rather stable. Hydrolysis of peptide bonds at the C-terminal end outside the cyclic structure contributed mainly to the biotransformation of anabaenopeptins. However, when the C-terminus of anabaenopeptins were occupied by apolar, aromatic amino acids, this biotransformation pathway was not occurring and these anabaenopeptins were significantly more stable. Deamination of arginine and tyrosine oxidation was observed for both microcystins and anabaenopeptins. However, tyrosine oxidation in anabaenopeptins was only observed when an arginine but not when apolar amino acids were at the C-terminal end, suggesting an interdependence of these reactions that needs to be further investigated. These tentatively identified reactions present universal pathways for biotransformation not expected to stem from specialized communities and further supports that the selection of organisms and enzymatic pathways capable of degrading microcystins and anabaenopeptins is more diverse than previously known. These novel insights into biotransformation of a wide range of cyanopeptides can facilitate the assessment of exposure scenarios in surface waters. Exposure is not only defined by the mixture of cynaopeptides produced by cyanobacteria, but also their lifetimes in surface waters and potentially bioactive transformation products. More persistent cyanopeptides dominate the exposure profile over time and new transformation products add to the profile and their potential toxicological relevance must be assessed and account for.

## Supplementary Material





## Notes

Detailed methodology (Text S1–S4); biotransformation experimental overview and details, reference standards and bioreagents, removal of cyanopeptides, kinetic analysis parameters, biphasic exponential decay model (Tables S1–S5); and images of the growth of biofilm, biotransformation kinetics, biotransformation of benzoic acid, removal of cyanopeptides, and half-lives (Figures S1–S11) (PDF)
Parent metabolites and transformation products lists, and their annotation details (XLSX)

## References

[1] Janssen E. M. L. (2019). Cyanobacterial
peptides beyond microcystins - A review on co-occurrence, toxicity,
and challenges for risk assessment. Water Res..

[2] Chorus, I. , Welker, M. , Eds. Toxic Cyanobacteria in Water: A Guide to Their Public Health Consequences, Monitoring and Management (2nd ed.); CRC Press, 2021; DOI: 10.1201/9781003081449.

[3] Janssen, E. M.-L. ; Jones, M. R. ; Pinto, E. ; Dörr, F. ; Torres, M. A. ; Rios Jacinavicius, F. ; Mazur-Marzec, H. ; Szubert, K. ; Konkel, R. ; Tartaglione, L. ; Dell’Aversano, C. ; Miglione, A. ; McCarron, P. ; Beach, D. G. ; Miles, C. O. ; Fewer, D. P. ; Sivonen, K. ; Jokela, J. ; Wahlsten, M. ; Niedermeyer, T. H. J. ; Schanbacher, F. ; Leão, P. ; Preto, M. ; D’Agostino, P. M. ; Baunach, M. ; Dittmann, E. ; Miguel-Gordo, M. ; Reher, R. ; Sieber, S. S75 | CyanoMetDB | Comprehensive database of secondary metabolites from cyanobacteria. 2024; DOI: 10.5281/zenodo.13854577. 33765498

[4] Le
Manach S., Duval C., Marie A., Djediat C., Catherine A., Edery M., Bernard C., Marie B. (2019). Global Metabolomic
Characterizations of Microcystis spp. Highlights Clonal Diversity
in Natural Bloom-Forming Populations and Expands Metabolite Structural
Diversity. Frontiers in Microbiology.

[5] Natumi R., Janssen E. M. (2020). Cyanopeptide Co-Production
Dynamics beyond Mirocystins
and Effects of Growth Stages and Nutrient Availability. Environ. Sci. Technol..

[6] Welker M., von Dohren H. (2006). Cyanobacterial
peptides - Nature’s own combinatorial
biosynthesis. Fems Microbiology Reviews.

[7] Jones M. R., Pinto E., Torres M. A., Dorr F., Mazur-Marzec H., Szubert K., Tartaglione L., Dell’Aversano C., Miles C. O., Beach D. G., McCarron P., Sivonen K., Fewer D. P., Jokela J., Janssen E. M. L. (2021). CyanoMetDB, a
comprehensive public database of secondary metabolites from cyanobacteria. Water Res..

[8] Beversdorf L. J., Weirich C. A., Bartlett S. L., Miller T. R. (2017). Variable Cyanobacterial
Toxin and Metabolite Profiles across Six Eutrophic Lakes of Differing
Physiochemical Characteristics. Toxins.

[9] Beversdorf L. J., Rude K., Weirich C. A., Bartlett S. L., Seaman M., Kozik C., Biese P., Gosz T., Suha M., Stempa C., Shaw C., Hedman C., Piatt J. J., Miller T. R. (2018). Analysis of cyanobacterial
metabolites in surface and
raw drinking waters reveals more than microcystin. Water Res..

[10] Gkelis S., Lanaras T., Sivonen K. (2015). Cyanobacterial Toxic
and Bioactive
Peptides in Freshwater Bodies of Greece: Concentrations, Occurrence
Patterns, and Implications for Human Health. Marine Drugs.

[11] Filatova D., Jones M. R., Haley J. A., Núñez O., Farré M., Janssen E. M. L. (2021). Cyanobacteria and their secondary
metabolites in three freshwater reservoirs in the United Kingdom. Environmental Sciences Europe.

[12] Miller T. R., Bartlett S. L., Weirich C. A., Hernandez J. (2019). Automated
Subdaily Sampling of Cyanobacterial Toxins on a Buoy Reveals New Temporal
Patterns in Toxin Dynamics. Environ. Sci. Technol..

[13] Wang X., Wullschleger S., Jones M., Reyes M., Bossart R., Pomati F., Janssen E. M. L. (2024). Tracking Extensive Portfolio of Cyanotoxins
in Five-Year Lake Survey and Identifying Indicator Metabolites of
Cyanobacterial Taxa. Environ. Sci. Technol..

[14] Itou Y., Suzuki S., Ishida K., Murakami M. (1999). Anabaenopeptins G and
H, Potent Carboxypeptidase A inhibitors from the cyanobacterium Oscillatoria
agardhii (NIES-595. Bioorg. Med. Chem. Lett..

[15] Spoof L., Błaszczyk A., Meriluoto J., Cegłowska M., Mazur-Marzec H. (2016). Structures and Activity of New Anabaenopeptins Produced
by Baltic Sea Cyanobacteria. Mar Drugs.

[16] Ishida K., Kato T., Murakami M., Watanabe M., Watanabe M. F. (2000). Microginins,
Zinc Metalloproteases Inhibitors from the Cyanobacterium Microcystis
aeruginosa. Tetrahedron.

[17] Chen X. G., Xiang H. Y., Hu Y., Zhang Y., Ouyang L., Gao M. Y. (2014). Fates of Microcystis
aeruginosa Cells and Associated
Microcystins in Sediment and the Effect of Coagulation Process on
Them. Toxins.

[18] Perez S., Aga D. S. (2005). Recent advances
in the sample preparation, liquid chromatography
tandem mass spectrometric analysis and environmental fate of microcystins
in water. Trac-Trends in Analytical Chemistry.

[19] Schmidt J. R., Wilhelm S. W., Boyer G. L. (2014). The Fate of Microcystins in the Environment
and Challenges for Monitoring. Toxins.

[20] Song L. R., Chen W., Peng L., Wan N., Gan N. Q., Zhang X. M. (2007). Distribution and bioaccumulation of microcystins in
water columns: A systematic investigation into the environmental fate
and the risks associated with microcystins in Meiliang Bay, Lake Taihu. Water Res..

[21] Kurtz T., Zeng T., Rosario-Ortiz F. L. (2021). Photodegradation
of cyanotoxins in
surface waters. Water Res..

[22] Natumi R., Marcotullio S., Janssen E. M. L. (2021). Phototransformation kinetics of cyanobacterial
toxins and secondary metabolites in surface waters. Environmental Sciences Europe.

[23] Natumi R., Dieziger C., Janssen E. M. L. (2021). Cyanobacterial
Toxins and Cyanopeptide
Transformation Kinetics by Singlet Oxygen and pH-Dependence in Sunlit
Surface Waters. Environ. Sci. Technol..

[24] Ishii H., Nishijima M., Abe T. (2004). Characterization of degradation process
of cyanobacterial hepatotoxins by a gram-negative aerobic bacterium. Water Res..

[25] Kato H., Imanishi S. Y., Tsuji K., Harada K. (2007). Microbial degradation
of cyanobacterial cyclic peptides. Water Res..

[26] Kiviranta J., Sivonen K., Lahti K., Luukkainen R., Niemela S. I. (1991). PRODUCTION AND BIODEGRADATION OF CYANOBACTERIAL TOXINS
- A LABORATORY STUDY. Archiv Fur Hydrobiologie.

[27] Mohamed Z., Alamri S., Hashem M. (2022). Simultaneous biodegradation of harmful
Cylindrospermopsis raciborskii and cylindrospermopsin toxin in batch
culture by single Bacillus strain. Environmental
Science and Pollution Research.

[28] Mohamed Z. A., Alamri S. A. (2012). Biodegradation of
cylindrospermopsin toxin by microcystin-degrading
bacteria isolated from cyanobacterial blooms. Toxicon.

[29] Ndlela L. L., Oberholster P. J., Van Wyk J. H., Cheng P. H. (2019). A laboratory
based
exposure of Microcystis and Oscillatoria cyanobacterial isolates to
heterotrophic bacteria. Toxicon.

[30] Dziga D., Maksylewicz A., Maroszek M., Budzyńska A., Napiorkowska-Krzebietke A., Toporowska M., Grabowska M., Kozak A., Rosińska J., Meriluoto J. (2017). The biodegradation of microcystins in temperate freshwater
bodies with previous cyanobacterial history. Ecotoxicology and Environmental Safety.

[31] Babica P., Bláha L., Marsálek B. (2005). Removal of microcystins by phototrophic
biofilms. A microcosm study. Environ. Sci. Pollut
Res. Int..

[32] Martinez
i Quer A., Larsson Y., Johansen A., Arias C. A., Carvalho P. N. (2024). Cyanobacterial blooms in surface waters – Nature-based
solutions, cyanotoxins and their biotransformation products. Water Res..

[33] Ding Q., Liu K., Xu K., Sun R., Zhang J., Yin L., Pu Y. (2018). Further Understanding
of Degradation Pathways of Microcystin-LR by
an Indigenous Sphingopyxis sp. in Environmentally Relevant Pollution
Concentrations. Toxins.

[34] Wei J., Pengji Z., Zhang J., Peng T., Luo J., Yang F. (2023). Biodegradation of MC-LR
and its key bioactive moiety Adda by Sphingopyxis
sp. YF1: Comprehensive elucidation of the mechanisms and pathways. Water Res..

[35] Bourne D. G., Jones G. J., Blakeley R. L., Jones A., Negri A. P., Riddles P. (1996). Enzymatic pathway for the bacterial
degradation of
the cyanobacterial cyclic peptide toxin microcystin LR. Appl. Environ. Microbiol..

[36] Bourne D. G., Riddles P., Jones G. J., Smith W., Blakeley R. L. (2001). Characterisation
of a gene cluster involved in bacterial degradation of the cyanobacterial
toxin microcystin LR. Environmental Toxicology.

[37] Tellenbach C., Tardent N., Pomati F., Keller B., Hairston N. G., Wolinska J., Spaak P. (2016). Cyanobacteria facilitate
parasite epidemics in Daphnia. Ecology.

[38] Navarro E., Robinson C. T., Wagner B., Behra R. (2007). Influence of Ultraviolet
Radiation on UVR-Absorbing Compounds in Freshwater Algal Biofilms
and Scenedesmus vacuolatus Cultures. Journal
of Toxicology and Environmental Health, Part A.

[39] Gil-Allué C., Schirmer K., Tlili A., Gessner M. O., Behra R. (2015). Silver Nanoparticle
Effects on Stream Periphyton During Short-Term Exposures. Environ. Sci. Technol..

[40] Davis C. A., Janssen E. M. L. (2020). Environmental
fate processes of antimicrobial peptides
daptomycin, bacitracins, and polymyxins. Environ.
Int..

[41] Thomas M. K., Fontana S., Reyes M., Kehoe M., Pomati F. (2018). The predictability
of a lake phytoplankton community, over time-scales of hours to years. Ecology Letters.

[42] Tlili A., Hollender J., Kienle C., Behra R. (2017). Micropollutant-induced
tolerance of in situ periphyton: Establishing causality in wastewater-impacted
streams. Water Res..

[43] Janssen, E. M.-L. ; Jones, M. R. ; Pinto, E. ; Dörr, F. ; Torres, M. A. ; Rios Jacinavicius, F. ; Mazur-Marzec, H. ; Szubert, K. ; Konkel, R. ; Tartaglione, L. ; Dell’Aversano, C. ; Miglinone, A. ; McCarron, P. ; Beach, D. G. ; Miles, C. O. ; Fewer, D. P. ; Sivonen, K. ; Jokela, J. ; Wahlsten, M. ; Niedermeyer, T. H. J. ; Schnbacher, F. ; Leão, P. ; Preto, M. ; D’Agostino, P. M. ; Baunach, M. ; Dittmann, E. ; Raphael, R. Dataset S75: CyanoMetDB-Comprehensive database of secondary metabolites from cyanobacteria; Zenodo, 2023; Vol. 3, DOI: 10.5281/zenodo.7922070.

[44] Steiner T., Schanbacher F., Lorenzen W., Enke H., Janssen E. M. L., Niedermeyer T. H. J., Gademann K. (2024). UV–vis absorbance
spectra, molar extinction coefficients and circular dichroism spectra
for the two cyanobacterial metabolites anabaenopeptin A and anabaenopeptin
B. Data in Brief.

[45] Schymanski E. L., Jeon J., Gulde R., Fenner K., Ruff M., Singer H. P., Hollender J. (2014). Identifying
Small Molecules via High
Resolution Mass Spectrometry: Communicating Confidence. Environ. Sci. Technol..

[46] Torres M. d. A., Dax A., Grand I., vom Berg C., Pinto E., Janssen E. M.-L. (2024). Lethal and behavioral
effects of semi-purified microcystins,
Micropeptin and apolar compounds from cyanobacteria on freshwater
microcrustacean Thamnocephalus platyurus. Aquatic
Toxicology.

[47] Torres M. d. A., Jones M. R., vom Berg C., Pinto E., Janssen E. M. L. (2023). Lethal
and sublethal effects towards zebrafish larvae of microcystins and
other cyanopeptides produced by cyanobacteria. Aquatic Toxicology.

[48] Rougé V., von Gunten U., Janssen E. M. L. (2024). Reactivity of
Cyanobacteria Metabolites
with Ozone: Multicompound Competition Kinetics. Environ. Sci. Technol..

[49] Wicker J., Lorsbach T., Gütlein M., Schmid E., Latino D., Kramer S., Fenner K. (2016). enviPath –
The environmental
contaminant biotransformation pathway resource. Nucleic Acids Res..

[50] Djoumbou-Feunang Y., Fiamoncini J., Gil-de-la-Fuente A., Greiner R., Manach C., Wishart D. S. (2019). BioTransformer:
a comprehensive computational tool
for small molecule metabolism prediction and metabolite identification. Journal of Cheminformatics.

[51] Hafner J., Lorsbach T., Schmidt S., Brydon L., Dost K., Zhang K., Fenner K., Wicker J. (2024). Advancements
in biotransformation
pathway prediction: enhancements, datasets, and novel functionalities
in enviPath. Journal of Cheminformatics.

[52] Maraccini P. A., Wenk J., Boehm A. B. (2016). Photoinactivation
of Eight Health-Relevant
Bacterial Species: Determining the Importance of the Exogenous Indirect
Mechanism. Environ. Sci. Technol..

[53] Edwards C., Graham D., Fowler N., Lawton L. A. (2008). Biodegradation of
microcystins and nodularin in freshwaters. Chemosphere.

[54] Hyenstrand P., Rohrlack T., Beattie K. A., Metcalf J. S., Codd G. A., Christoffersen K. (2003). Laboratory studies of dissolved radiolabelled microcystin-LR
in lake water. Water Res..

[55] Christoffersen K, Lyck S, Winding A (2002). Microbial activity
and bacterial community structure
during degradation of microcystins. Aquatic
Microbial Ecology.

[56] Bourne D. G., Blakeley R. L., Riddles P., Jones G. J. (2006). Biodegradation of
the cyanobacterial toxin microcystin LR in natural water and biologically
active slow sand filters. Water Res..

[57] Li J., Li R., Li J. (2017). Current research scenario for microcystins biodegradation
– A review on fundamental knowledge, application prospects
and challenges. Science of The Total Environment.

[58] Seller-Brison C., Brison A., Yu Y., Robinson S. L., Fenner K. (2024). Adaptation
towards catabolic biodegradation of trace organic contaminants in
activated sludge. Water Res..

[59] Ren L., Hu Z., Wang Q., Du Y., Zong W. (2020). Regulation Efficacy
and Mechanism of the Toxicity of Microcystin-LR Targeting Protein
Phosphatase 1 via the Biodegradation Pathway. Toxins.

[60] Helbling D. E., Johnson D. R., Honti M., Fenner K. (2012). Micropollutant Biotransformation
Kinetics Associate with WWTP Process Parameters and Microbial Community
Characteristics. Environ. Sci. Technol..

[61] Nguyen T.
T. H., Myrold D. D., Mueller R. S. (2019). Distributions of Extracellular Peptidases
Across Prokaryotic Genomes Reflect Phylogeny and Habitat. Frontiers in Microbiology.

[62] Faccio G., Kruus K., Saloheimo M., Thöny-Meyer L. (2012). Bacterial
tyrosinases and their applications. Process
Biochemistry.

[63] Min K., Park G. W., Yoo Y. J., Lee J.-S. (2019). A perspective on
the biotechnological applications of the versatile tyrosinase. Bioresour. Technol..

[64] Panis F., Rompel A. (2023). Biochemical Investigations
of Five Recombinantly Expressed
Tyrosinases Reveal Two Novel Mechanisms Impacting Carbon Storage in
Wetland Ecosystems. Environ. Sci. Technol..

[65] Requena J. R., Levine R. L., Stadtman E. R. (2003). Recent advances in the analysis of
oxidized proteins. Amino Acids.

[66] Novák L., Zubáčová Z., Karnkowska A., Kolisko M., Hroudová M., Stairs C. W., Simpson A. G. B., Keeling P. J., Roger A. J., Čepička I., Hampl V. (2016). Arginine deiminase
pathway enzymes: evolutionary history in metamonads
and other eukaryotes. BMC Evolutionary Biology.

[67] Cunin R., Glansdorff N., Piérard A., Stalon V. (1986). Biosynthesis and metabolism
of arginine in bacteria. Microbiol Rev..

[68] Ohura T., Kominami E., Tada K., Katunuma N. (1982). Crystallization and
properties of human liver ornithine aminotransferase. J. Biochem.

[69] Phang J. M., Liu W., Hancock C., Christian K. J. (2012). The proline regulatory axis and cancer. Front Oncol.

[70] Stránská J., Kopečný D., Tylichová M., Snégaroff J., Šebela M. (2008). Ornithine
δ-aminotransferase. Plant Signaling &
Behavior.

[71] Yasuda M., Misono H., Soda K., Yonaha K., Toyama S. (1979). Purification
and crystallization of L-ornithine:alpha-ketoglutarate delta-aminotransferase
from Bacillus sphaericus. FEBS Lett..

[72] Niedermeyer T. H. J., Daily A., Swiatecka-Hagenbruch M., Moscow J. A. (2014). Selectivity
and Potency of Microcystin Congeners against OATP1B1 and OATP1B3 Expressing
Cancer Cells. PLoS One.

[73] Bouaicha N., Miles C. O., Beach D. G., Labidi Z., Djabri A., Benayache N. Y., Nguyen-Quang T. (2019). Structural Diversity, Characterization
and Toxicology of Microcystins. Toxins.

