# Supplementary material for: Biotransformation Dynamics and Products of Cyanobacterial Secondary Metabolites in Surface Waters
Source: Environ Sci Technol. 2025 Sep 20;59(38):20726–37. doi: 10.1021/acs.est.5c09247 (PMC12490008; doi:10.1021/acs.est.5c09247)
Supplement: Supplementary file 1 [file es5c09247_si_001.pdf]

Biotransformation dynamics and products of cyanobacterial secondary metabolites in surface waters

Xuejian Wang,[1] Andrea Ingold[1], Elisabeth M.-L. Janssen[1*]

[1] *Swiss Federal Institute of Aquatic Science and Technology (EAWAG), Dübendorf 8600, Switzerland.*

*Corresponding author: elisabeth.janssen@eawag.ch

Supporting Information

The electronic supporting information contains 35 pages including 4 text, 5 tables and 11 figures.

List of Text

Text S1. Cyanobacterial culture condition…………………………………………………...S3

Text S2. Benzoic acid analysis…………...………………………………………………S4

Text S3. Estimation of LOD………………………...………………………...……...…S4

Text S4. Evaluation of shoulder-log fitting…………………………………...……...…S4

List of Tables

Table S1. Biotransformation experiment details…………………………………….....S5

Table S2. Reference standards and bioreagents……………………………………......S6

Table S3. 3d removal in Chriesbach, MA and Glatt……………………………………….S7

Table S4. Lag-phase and initial removal……………………………………………......S8

Table S5. Biphasic exponential decay model……………………………………………S8

List of Figures

Figure S1. Imaged biofilm growth……………………………………………...………S9

Figure S2. Kinetics in Chriesbach, MA and Glatt…………………………………..……S10

Figure S3. BZA kinetics in Chriesbach, MA and Glatt………………………………..…S21

Figure S4. Abiotic decreased metabolites………………………………………..…..…..S21

Figure S5. Kinetics metabolite dilution and biofilm dilution…………………….….….…S22

Figure S6. Half-lives of three metabolites in diluted Chriesbach biofilm suspension……..S30

Figure S7. Kinetics of additional metabolites from Brazilian strains……..……….………S31

Figure S8. Kinetics of additional metabolites from Greifensee isolates……………………S33

Figure S9. Transformation product for [D-Asp^3, (*E*)D-hb^7]MC-RR ………………………S34

Figure S10. Transformation product for MC-HtyR ……………………………...……S34

Figure S11. Transformation product for Anabaenopeptin F S35

Text S1. Cyanobacterial cultures and growth conditions.

Microcystis aeruginosa PCC7806 was originally isolated from Braakman reservoir in The Netherlands (1972) and was obtained from the Pasteur Culture Collection of Cyanobacteria (France). *Dolichospermum flos aquae* NIVA-CYA 269/6 was originally isolated from Lake Frøylandsvatnet in Norway (1990) and *Planktothrix rubescence* K-0576 was originally isolated from Lake Borre Sø in Denmark, both strains were obtained from the Norwegian Culture Collection of Algae (NORCCA). *Microcystis aeruginosa* UV006 was originally isolated from Hartebeespoort Dam in South Africa and an inoculum was provided by Prof. Jakob Pernthaler (University of Zurich, Switzerland). *Microcystis panniformis* (MIRS-04, isolated from the Samuel Reservoir in Rondônia, Brazil), and *Microcystis aeruginosa* (NPDC-01, isolated from a sewage treatment plant in Cidade de Deus, Rio de Janeiro, Brazil) were obtained from the culture collection of the Laboratory of Ecophysiology and Toxicology of Cyanobacteria, at the Federal University of Rio de Janeiro (Brazil). *Microcystis* G2011, *Microcystis* G2020 and *Planktothrix* G2020 were originally isolated from Lake Greifensee in Switzerland (2011 and 2020).

Strains MIRS-04 and NPCD-01 were cultivated in ASM-1 medium (pH 7.5) for 20 days (Gorham et al., 1964), and culture flasks were maintained under a 12 h light/dark photoperiod, with light intensity of 20 μmol photons m-2s-1, continuous aeration, and temperature of 24 ± 2 ˚C for 20 days. After 20 days of growth, cells were harvested by centrifugation (9000 rpm at 15 ˚C, 10 min). The resulting biomass was stored at -20 ˚C, lyophilized (-55 ˚C, 500 μHg, 24 h, L101 Liotop® lyophilizer, Liobras, Brazil) (Torres et al., 2023). Other cyanobacterial strains were cultured in WC medium at 20 ± 2 ˚C and irradiated at 12 μmol photons m^{-2} s^{-1} on a 12:12-hlight/dark cycle (Guillard & Lorenzen, 2008). The cells were harvested by centrifugation (rcf of 4000 g at 10 ˚C, 10 min, Herolab HiCen XL), lyophilized (-40 ˚C, -3 mbar, 24 h, Lyovac GT2, Leybold) and stored at -20 ˚C until metabolite extraction.

References

1. Gorham, P. R., McLachlan, J., Hammer, U. T., & Kim, W. K. (1964). Isolation and culture of toxic strains of Anabaena flos-aquae (Lyngb.) de Bréb. SIL Proceedings, 1922-2010, 15(2), 796-804. https://doi.org/10.1080/03680770.1962.11895606

2. Torres, M. d. A., Jones, M. R., vom Berg, C., Pinto, E., & Janssen, E. M. L. (2023). Lethal and sublethal effects towards zebrafish larvae of microcystins and other cyanopeptides produced by cyanobacteria. Aquatic Toxicology, 263, 106689. https://doi.org/https://doi.org/10.1016/j.aquatox.2023.106689

3. Guillard, R., & Lorenzen, C. (2008). Yellow-greenalgae with Chlorophyllide. Journal of Phycology, 8, 10-14. https://doi.org/10.1111/j.1529-8817.1972.tb03995.x

Text S2. Benzoic acid analysis.

The benzoic acid analysis was performed by UHPLC (Dionex UltiMate 3000 RS Pump and RS Autosampler, Thermo Scientific) coupled to a UV/VIS detector (Dionex UltiMate 3000 RS Diode Array Detector, Thermo Scientific). For the chromatographic separation, a XBridge C18 column (3.5 µm, 2.1 × 50 mm, Waters) was used. The mobile phases were nanopure water (A) and acetonitrile (B) both acidified with formic acid (0.1%). The measurement was run at a flow rate of 350 µL/min at 70% of B for 5 min and the injection volume was 20 µL. The detection of benzoic acid was achieved by measuring the UV absorbance at 245 nm as well as comparison of the retention time with a reference standard.

Text S3. Estimation of Limits of Detection.

For metabolites, identified on confidence level 2, the limit of detection (LOD) of a structural similar reference standards or bioreagents were used to calculate k_{bio} value of biofilm dilution experiment: oscillamide Y for anabaenopeptin F; cyanopeptolin D for cyanopeptolin B and C; cyanopeptolin A for cyanopeptolin 1020 and 963A; MC-LA for MC-LL, MC-LAba and [D-Asp^3]MC-LA; MC-YR for MC-HtyR; MC-LR for [D-Asp^3]MC-(H4)YR.

Text S4. Evaluation of shoulder-log fitting.

Model fit quality was assessed using a pseudo R^2, calculated as below, and by computing a chi-square (χ^2) statistic along with its corresponding p-value to evaluate the discrepancy between observed and predicted values. Fitted parameters (k, S, and C_0), their standard deviation, half-life estimates, pseudo R^2 values, and χ^2 statistics were recoded and used to compare decay kinetics across different groups. The cutting threshold for pseudo R^2 is 0.6 in this study.

$$R^2 = 1 - \frac{\sum \left(C_{observed} - C_{predicted}\right)^2}{\sum \left(C_{observed} - \overline{C}_{observed}\right)^2}$$

Table S1. Biotransformation experimental overview and details.

Biotransformation experiments		1	2	3	4	5
		Comparison surface water and biofilm and different sites	Biofilm dilutions	Metabolite dilutions	Metabolites from Brazilian strains	Metabolites from Lake Greifensee isolates
**Biofilm incubation**	river	Chriesbach, Glatt, Mönchaltorder Aa	Chriesbach	Chriesbach	Chriesbach	Chriesbach
	biofilm grew time	Chriesbach: 25.03.2022-25.04.2022<br>Glatt: 15.06.2022-12.07.2022<br>MA: 15.06.2022-12.07.2022	24.02.2023/03.03.2023 -21.04.2023	19.06.2023-21.07.2023	12.08.2024-13.09.2024	08.10.2024-15.11.2024
	average water temparature/˚C	17.75 ± 3.23	14	18.11 ± 1.36	17.93 ± 0.81	15.08 ± 1.77
	river flow rate/m/s	Chriesbach: 0.06-0.1<br>Glatt: 0.1-0.7<br>MA: 0.05-0.3	0.07-0.08	0.06-0.1	0.06-0.1	0.06-0.1
**Toxin extraction**	cyanobacterial strains	*M. aeruginosa* PCC7806<br>*P. rubescens*<br>UV *M. aeruginosa* 006<br>*D. flos aquae*	*M. aeruginosa* PCC7806<br>*P. rubescens*<br>UV *M. aeruginosa* 006<br>*D. flos aquae*	*M. aeruginosa* PCC7806<br>*P. rubescens*<br>UV *M. aeruginosa* 006<br>*D. flos aquae*	*M. aeruginosa* NPDC-1<br>*M. panniformis* MIRS-04	M. G2011<br>M. G2020<br>P. G2020
**Experiment setups**	initial BZA concentration / µM	30	30	30	30	30
	EtOH concentration in reactor/ mM	0.4	13.3	13.3	39.4	no EtOH
	sub-sampling time/ d	0, 1, 3, 5, 7, 10	0, 0.08, 0.17, 0.33, 1, 1.17, 1.33, 2, 3, 5, 7	0, 0.17, 0.33, 1, 1.33, 2, 2.33, 3, 3.33, 4	0, 0.17, 0.33, 1, 1.33, 2, 2.33, 3, 3.33, 5	0, 0.25, 0.5, 1, 1.25, 1.5, 2, 2.25, 2.5, 3, 4

Table S2. Reference standards and bioreagents used in the metabolite analysis.
Reference standards and bioreagents used in the metabolite analysis with their molecular formula, monoisotopic mass, and dominant precursor ion form with associated mass-to-charge (m/z) ratio.

**metabolite**	**molecular formula**	**Monoisotopic mass (Da; rounded to 5 d.p.)**	**dominant precursor ion and *m*/*z* value**	
Aerucyclamide A	$C_{24}H_{34}N_6O_4S_2$	534.20830	$[M+H]^+$	535.21557
Anabaenopeptin A	$C_{44}H_{57}N_7O_{10}$	843.41669	$[M+H]^+$	844.42397
Anabaenopeptin B	$C_{41}H_{60}N_{10}O_9$	836.45447	$[M+H]^+$	837.46175
Oscillamide Y	$C_{45}H_{59}N_7O_{10}$	857.43234	$[M+H]^+$	858.43962
Cyanopeptolin A	$C_{46}H_{72}N_{10}O_{12}$	956.53312	$[M+H]^+$	957.54039
Cyanopeptolin D	$C_{48}H_{76}N_8O_{12}$	956.55827	$[M+H]^+$	957.56555
MC-LR	$C_{49}H_{74}N_{10}O_{12}$	994.54877	$[M+H]^+$	995.55604
MC-HilR	$C_{50}H_{76}N_{10}O_{12}$	1008.56442	$[M+H]^+$	1009.57169
MC-LA	$C_{46}H_{67}N_7O_{12}$	909.48477	$[M+H]^+$	910.49205
MC-LF	$C_{52}H_{71}N_7O_{12}$	985.51607	$[M+H]^+$	986.52335
MC-LW	$C_{54}H_{72}N_8O_{12}$	1024.52697	$[M+H]^+$	1025.53425
MC-LY	$C_{52}H_{71}N_7O_{13}$	1001.51099	$[M+H]^+$	1002.51826
MC-RR	$C_{49}H_{75}N_{13}O_{12}$	1037.56581	$[M+2H]^{2+}$	519.79018
MC-YR	$C_{52}H_{72}N_{10}O_{13}$	1044.52803	$[M+H]^+$	1045.53531
[D-Asp³,(*E*)-Dhb⁷]MC-RR	$C_{48}H_{73}N_{13}O_{12}$	1023.55016	$[M+2H]^{2+}$	512.78236
[D-Asp³]MC-LR	$C_{48}H_{72}N_{10}O_{12}$	980.53312	$[M+H]^+$	981.54039
Nodularin-R	$C_{41}H_{60}N_8O_{10}$	824.44324	$[M+H]^+$	825.45052
Aeruginosin 98B	$C_{29}H_{46}N_6O_9S$	654.30470	$[M+H]^+$	655.31197

Other Materials. Microcystin reference standards MC-LR, MC-YR, MC-RR, MC-LF, MC-LA, MC-LW, MC-LY, and nodularin (all >95% purity by HPLC) were obtained from Enzo Life Science (Lausen, Switzerland) and [D-Asp^3, E-Dhb^7] MC-RR (>95% purity by HPLC) from CyanoBiotech GmbH (Berlin, Germany). Bioreagents for aeruginosin 98B, cyanopeptolin A, cyanopeptolin D, anabaenopeptin A, anabaenopeptin B, and oscillamide Y (all >90% purity by HPLC) were obtained from CyanoBiotech. Aerucyclamide A was obtained as purified bioreagent in dimethyl sulfoxide by Prof. Karl Gademann (University Zurich, Switzerland) (Portmann et al., 2008).

Reference

Portmann, C., Blom, J. F., Gademann, K., & Jüttner, F. (2008). Aerucyclamides A and B: Isolation and Synthesis of Toxic Ribosomal Heterocyclic Peptides from the Cyanobacterium Microcystis aeruginosa PCC 7806. Journal of Natural Products, 71(7), 1193-1196. https://doi.org/10.1021/np800118g

Table S3. Removal of cyanopeptides after 3-day exposure to biofilm suspensions from River Chriesbach, River Mönschaltorfer Aa (MA) and River Glatt.

3d	Chriesbach		MA		Glatt	
	Removal	stdev	Removal	stdev	Removal	stdev
Anabaneopeptin A	35%	14%	35%	42%	0%	19%
Anabaenopeptin B	74%	7%	39%	48%	18%	10%
Oscillamide Y	33%	13%	35%	42%	0%	17%
Anabaenopeptin F	75%	6%	45%	43%	21%	8%
Cyanopeptolin A	90%	2%	91%	6%	55%	9%
Cyanopeptolin B	81%	3%	76%	15%	51%	10%
Cyanopeptolin C	61%	6%	48%	32%	48%	32%
Cyanopeptolin D	54%	8%	28%	42%	34%	12%
Cyanopeptolin 1020	87%	4%	80%	14%	69%	27%
Cyanopeptolin 963A	32%	16%	30%	44%	22%	13%
Aerucylamide A	20%	11%	18%	45%	13%	11%
MC-LR	18%	12%	14%	62%	5%	10%
[D-Asp3]MC-LR	18%	14%	19%	58%	5%	14%
MC-LA	12%	15%	12%	54%	5%	6%
MC-YR	30%	12%	28%	51%	2%	15%
MC-LL	13%	12%	26%	42%	21%	11%
MC-HtyR	31%	14%	29%	49%	16%	11%
[D-Asp3]MC-(H4)YR	86%	4%	81%	12%	81%	12%
[D-Asp3]MC-LA	1%	15%	13%	55%	8%	6%
MC-LAba	12%	14%	7%	57%	15%	11%
[D-Asp3EDhb7]MC-RR	46%	12%	12%	79%	15%	17%

Table S4. Parameters of kinetic analysis including lag-time, half-lives and initial removal for cyanopeptides when (A) the initial spiked metabolite mixture was diluted from 100% to 10% and 2% at constant biofilm concentration (100%), and when (B) the initial metabolite concentration was constant (100%) and the biofilm suspensions was diluted from 100% to 10% and 1%. Note: "0 h" lag time means, the model suggests no lag-phase (setting S=0 in the model) and the remaining model equals a pseudo first-order model fit; "*" means that the log-shoulder model performed poorly with $R^2 < 0.6$, and a bi-phasic-model was applied instead (Table S5); "n.d.(data)" means that parameters were not determined because data did not allow modeling due to lack of data from at least three time points above LOD; "n.d.(decay)" means no significant decay was observed with no statistically different concentration of final datapoints to data from timepoint zero.

A	Metabolite Dilution Experiment											
	Lag-time (h)			Half-lives (h)			1.3d removal					
							100%		10%		2%	
	100%	10%	2%	100%	10%	2%	removal	stdev	removal	stdev	removal	stdev
Anabaneopeptin A	34.8	12.3	6.7	34.9	13.4	7.2	46%	10%	98%	1%	100%	0%
Anabaenopeptin B	29.9	10.3	5.3	30.0	11.2	6.2	62%	9%	99%	1%	100%	0%
Oscillamide Y	33.8	11.7	6.8	33.9	12.8	7.1	51%	9%	98%	1%	100%	0%
Anabaenopeptin F	30.0	9.2	n.d.(data)	30.2	10.8	n.d.(data)	62%	9%	99%	0%	100%	0%
Cyanopeptolin A	0.0	6.1	n.d.(data)	5.8	6.5	n.d.(data)	97%	1%	100%	0%	100%	0%
Cyanopeptolin B	0.0	n.d.(data)	n.d.(data)	7.7	n.d.(data)	n.d.(data)	96%	3%	100%	0%	100%	0%
Cyanopeptolin C	9.6	8.5	n.d.(data)	18.1	8.5	n.d.(data)	82%	9%	100%	0%	100%	0%
Cyanopeptolin D	27.2	10.5	7.3	27.2	11.1	7.4	76%	11%	100%	0%	100%	0%
Cyanopeptolin 1020	0.4	8.3	5.7	9.9	8.3	6.2	94%	4%	100%	0%	100%	0%
Cyanopeptolin 963A	29.5	*	n.d.(data)	29.6	*	n.d.(data)	64%	11%	98%	3%	100%	0%
Aerucylamide A	56.9	34.8	28.0	56.9	34.9	28.4	21%	5%	43%	15%	64%	8%
MC-LR	75.5	45.5	41.3	75.6	45.5	41.3	14%	9%	15%	22%	28%	14%
[D-Asp3]MC-LR	60.4	40.7	*	60.4	40.7	*	11%	6%	20%	23%	37%	15%
MC-YR	56.0	32.6	n.d.(data)	56.1	32.6	n.d.(data)	19%	6%	32%	17%	53%	9%
MC-HtyR	70.1	n.d.(data)	n.d.(data)	70.4	n.d.(data)	n.d.(data)	9%	6%	14%	22%	33%	20%
[D-Asp3]MC-(H4)YR	1.1	n.d.(data)	n.d.(data)	11.0	n.d.(data)	n.d.(data)	94%	3%	100%	0%	100%	0%
[D-Asp3, (E)Dhb7]MC-RR	35.3	22.6	n.d.(data)	35.5	22.8	n.d.(data)	46%	7%	90%	8%	94%	6%
MC-LA	n.d. (decay)	n.d. (decay)	n.d.(data)	n.d. (decay)	n.d. (decay)	n.d.(data)	6%	6%	5%	20%	-1%	14%
MC-LAba	n.d. (decay)	n.d.(data)	n.d.(data)	n.d. (decay)	n.d.(data)	n.d.(data)	8%	8%	-2%	17%	-7%	13%
MC-LL	n.d. (decay)	n.d.(data)	n.d.(data)	n.d. (decay)	n.d.(data)	n.d.(data)	5%	8%	-1%	17%	44%	32%
[D-Asp3]MC-LA	n.d. (decay)	n.d.(data)	n.d.(data)	n.d. (decay)	n.d.(data)	n.d.(data)	3%	7%	-8%	21%	-7%	21%

B	Biofilm Dilution Experiment															
	Lag-time (h)				Half-lives (h)				1d removal							
									100%		10%		1%	River water		
	100%	10%	1%	River water	100%	10%	1%	River water	removal	stdev	removal	stdev	removal	stdev	removal	stdev
Anabaneopeptin A	10.2	102.8	98.1	n.d.(decay)	11.5	103.7	177.1	n.d.(decay)	86%	6%	25%	10%	-9%	16%	-28%	33%
Anabaenopeptin B	8.5	111.7	*	n.d.(decay)	10.4	111.9	*	n.d.(decay)	85%	5%	23%	9%	-11%	14%	-23%	28%
Oscillamide Y	9.4	99.6	0.0	n.d.(decay)	11.1	101.7	191.5	n.d.(decay)	86%	6%	28%	8%	1%	12%	-24%	25%
Anabaenopeptin F	9.9	106.2	*	n.d.(decay)	11.5	106.3	*	n.d.(decay)	83%	5%	28%	7%	-13%	12%	-44%	32%
Cyanopeptolin A	0.8	0.0	89.7	n.d.(decay)	5.1	35.9	105.9	n.d.(decay)	98%	0%	57%	9%	10%	27%	3%	30%
Cyanopeptolin B	n.d.(data)	n.d.(data)	n.d.(data)	n.d.(data)	n.d.(data)	n.d.(data)	n.d.(data)	n.d.(data)	100%	0%	49%	10%	16%	22%	2%	19%
Cyanopeptolin C	n.d.(data)	n.d.(data)	n.d.(data)	n.d.(data)	n.d.(data)	n.d.(data)	n.d.(data)	n.d.(data)	80%	9%	29%	18%	1%	24%	-3%	27%
Cyanopeptolin D	18.7	*	n.d.	n.d.(decay)	19.1	*	n.d.	n.d.(decay)	77%	10%	26%	17%	4%	22%	11%	18%
Cyanopeptolin 1020	6.7	60.5	97.7	128.5	9.0	64.2	112.1	129.5	91%	4%	31%	15%	5%	16%	-23%	15%
Cyanopeptolin 963A	n.d.(data)	n.d.(data)	n.d.(data)	n.d.(data)	n.d.(data)	n.d.(data)	n.d.(data)	n.d.(data)	56%	15%	5%	16%	-26%	27%	-81%	32%
Aerucylamide A	66.8	n.d.(decay)	n.d.(decay)	n.d.(decay)	66.9	n.d.(decay)	n.d.(decay)	n.d.(decay)	-2%	11%	-2%	9%	-14%	15%	-23%	20%
MC-LR	44.9	n.d.(decay)	n.d.(decay)	n.d.(decay)	45.2	n.d.(decay)	n.d.(decay)	n.d.(decay)	4%	14%	12%	8%	-9%	15%	-23%	28%
[D-Asp3]MC-LR	32.2	n.d.(decay)	n.d.(decay)	n.d.(decay)	32.6	n.d.(decay)	n.d.(decay)	n.d.(decay)	21%	14%	11%	14%	-19%	15%	-43%	28%
MC-YR	28.9	n.d.(decay)	n.d.(decay)	n.d.(decay)	30.9	n.d.(decay)	n.d.(decay)	n.d.(decay)	30%	8%	25%	11%	-11%	13%	-42%	36%
MC-HtyR	42.9	n.d.(decay)	n.d.(decay)	n.d.(decay)	51.8	n.d.(decay)	n.d.(decay)	n.d.(decay)	17%	6%	19%	11%	-13%	13%	-36%	32%
[D-Asp3]MC-(H4)YR	5.8	56.4	108.8	168.0	7.6	62.2	120.3	170.5	94%	6%	31%	12%	6%	7%	-24%	10%
[D-Asp3, (E)Dhb7]MC-RR	19.7	168.0	n.d.(decay)	n.d.(decay)	21.1	168.3	n.d.(decay)	n.d.(decay)	29%	10%	28%	15%	5%	32%	27%	33%
MC-LL	n.d.(decay)	n.d.(decay)	n.d.(decay)	n.d.(decay)	n.d.(decay)	n.d.(decay)	n.d.(decay)	n.d.(decay)	53%	10%	5%	6%	-11%	15%	-2%	16%
MC-LA	n.d.(decay)	n.d.(decay)	n.d.(decay)	n.d.(decay)	n.d.(decay)	n.d.(decay)	n.d.(decay)	n.d.(decay)	-8%	18%	11%	11%	-14%	16%	-19%	27%
[D-Asp3]MC-LA	n.d.(decay)	n.d.(decay)	n.d.(decay)	n.d.(decay)	n.d.(decay)	n.d.(decay)	n.d.(decay)	n.d.(decay)	-18%	21%	15%	5%	-13%	7%	-22%	18%
MC-LAba	n.d.(decay)	n.d.(decay)	n.d.(decay)	n.d.(decay)	n.d.(decay)	n.d.(decay)	n.d.(decay)	n.d.(decay)	-8%	11%	13%	8%	-6%	13%	-11%	14%

Table S5. Biphasic exponential decay model

A biphasic exponential decay model was used for 3 cases where the log-shoulder model did not allow for a satisfying fit (i.e., $R^2<0.6$): anabaenopeptin B (1% biofilm group) anabaenopeptin F (1% biofilm group) and cyanopeptolin D (10% biofilm group), using:

$$\frac{A}{A_0} = N_1 e^{-\alpha t} + N_2 e^{-\beta t}$$

with the coefficients N_1 and N_2, α being the rate constant of the first phase, β being the rate constant of the second phase with α>β (the first phase ends before the second one dominates).

The results for fitting with the biphasic model using all triplicates at each sampling time point (top) and using the average of the triplicates:

A	fitted with triplicates at each time						
Compound	N1	α (1/h)	N2	β (1/h)	half-life 1 (h)	half-life 2 (h)	R^2
cyanopeptolin D	0.25	8.63	0.75	0.00	0.08	287.49	0.51
anabaenopeptin B	-124.17	0.02	124.98	0.02	41.06	41.47	0.61
anabaenopeptin F	-162.11	0.02	162.93	0.02	37.65	37.98	0.60
B	fitted with the average of triplicates at each time						
Compound	N1	α (1/h)	N2	β (1/h)	half-life 1 (h)	half-life 2 (h)	R^2
cyanopeptolin D	0.24	9.45	0.76	0.00	0.07	285.66	0.76
anabaenopeptin B	-152.81	0.02	153.62	0.02	40.82	41.16	0.80
anabaenopeptin F	-196.29	0.02	197.11	0.02	37.29	37.56	0.77

Note that for cyanopeptolin 963A (10% metabolite group) and [D-Asp^3]MC-LR (2% metabolite group) in the first phase no decay was observed and only at the end of the experimental time, a sharp drop was observed below the limit of detection. The biphasic model was evaluated but did not provide a satisfying fit.

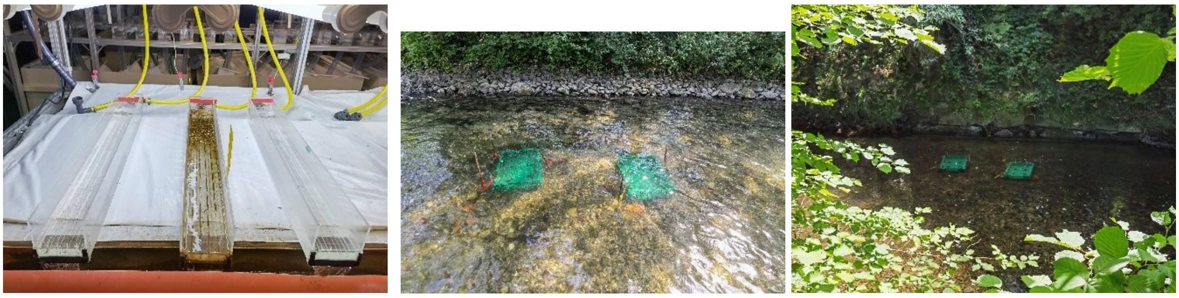

Figure S1. Images of the growth of biofilm in River Chriesbach where a bypass of the river water was diverted into laboratory growth channels with glass slides on which the biofilm established (left), in-situ biofilm growth on glass slides deployed in protective boxes (green) in River Glatt (middle) and River Mönschaltorfer Aa (right).

Figure S2 (continuing until page S20). Biotransformation kinetics of 21 cyanopeptides in River Chriesbach, River Glatt and River Mönschaltorfer Aa (MA) for biofilm suspensions and river water as well as abiotic controls denoted as “aBiofilm” for autoclaved biofilm suspension and “aWater” for autoclaved surface water. The fourth quadrant shows data for lake Greifensee surface water and abiotic control in anaopure water (NPwater) and autoclaved lake water (aWaterL). Data shows the detected peak area normalized to the initial peak area across the incubation time. Data points are connected by lines. Note: here not representing the shoulder-log model.

1. Aerucyclamide A

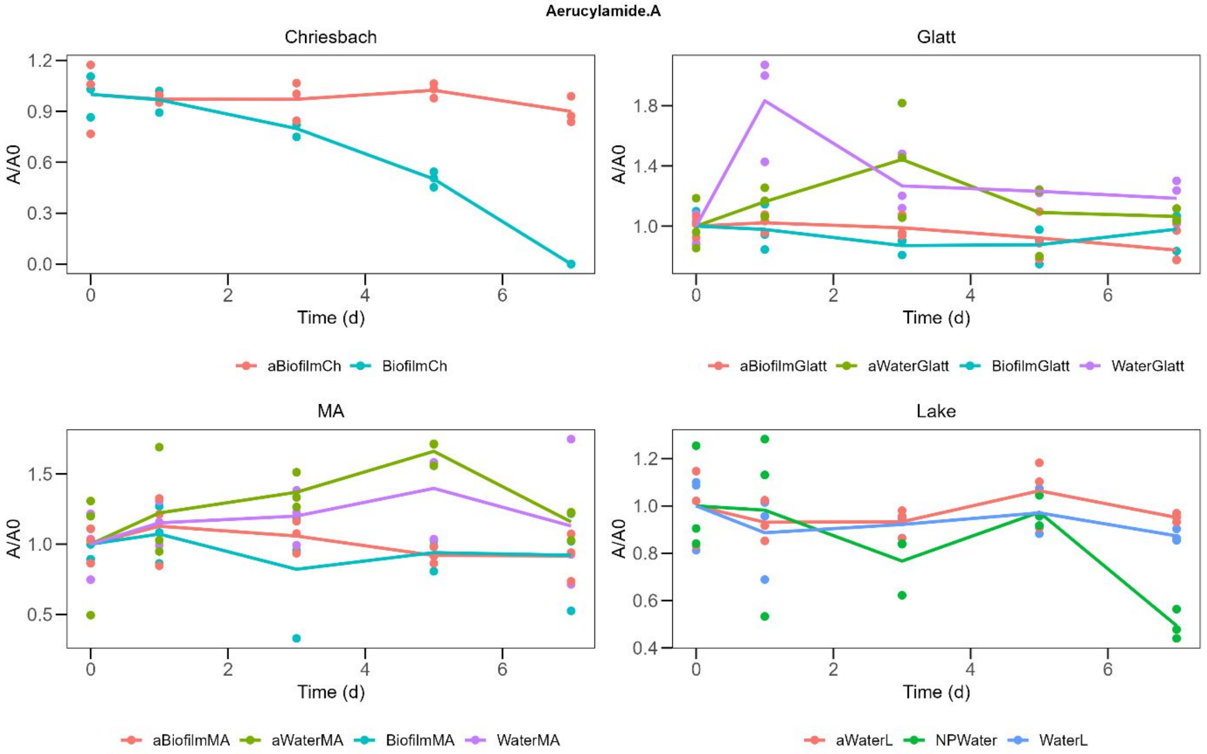

2. Anabaenopeptin B

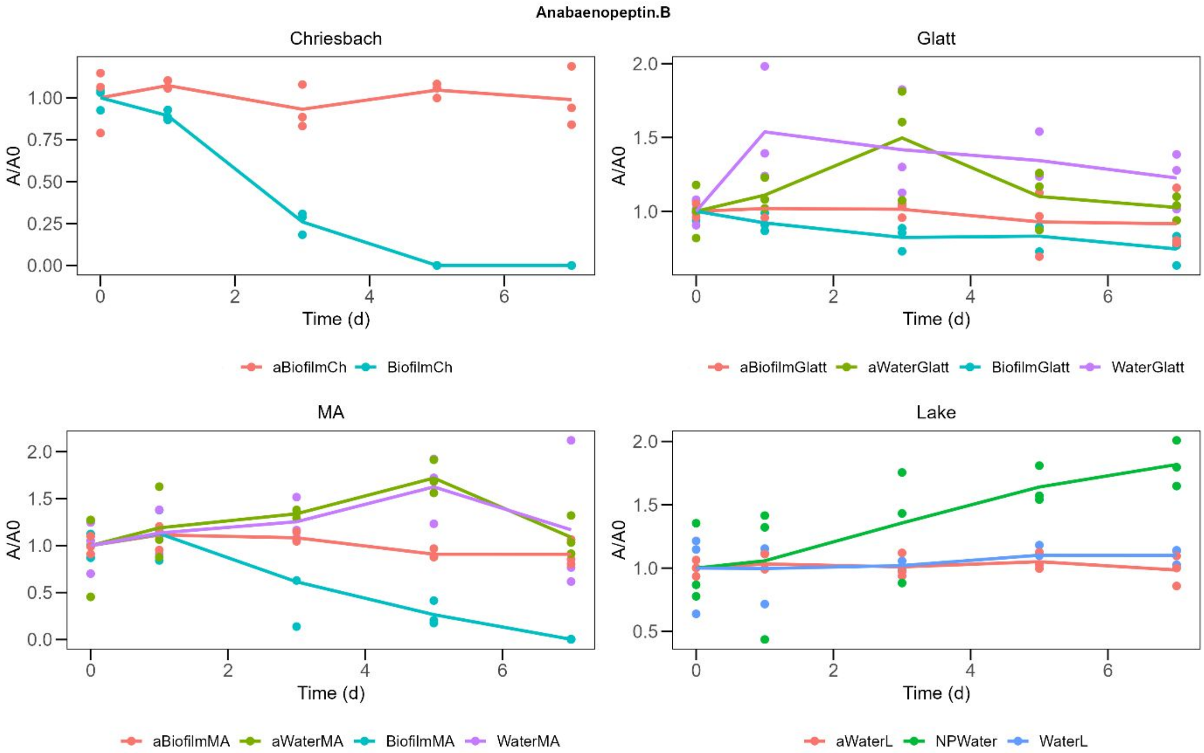

3. Anabaneopeptin F

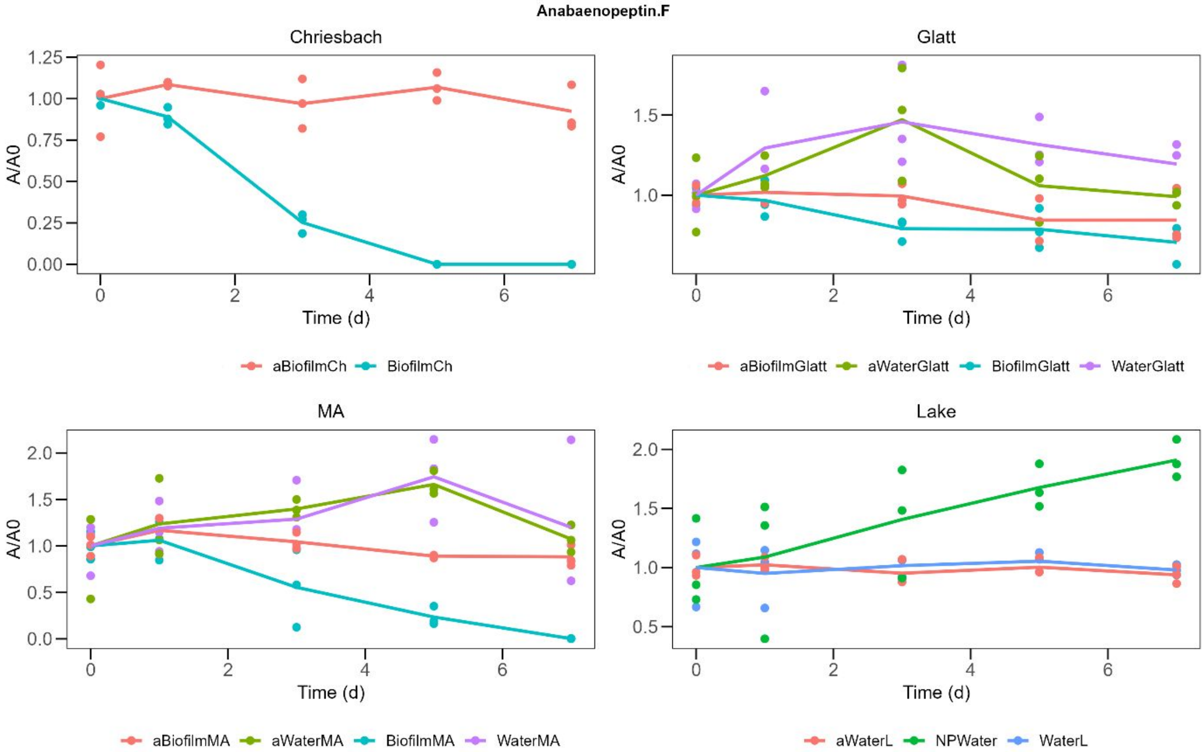

4. Anabaenopeptin A

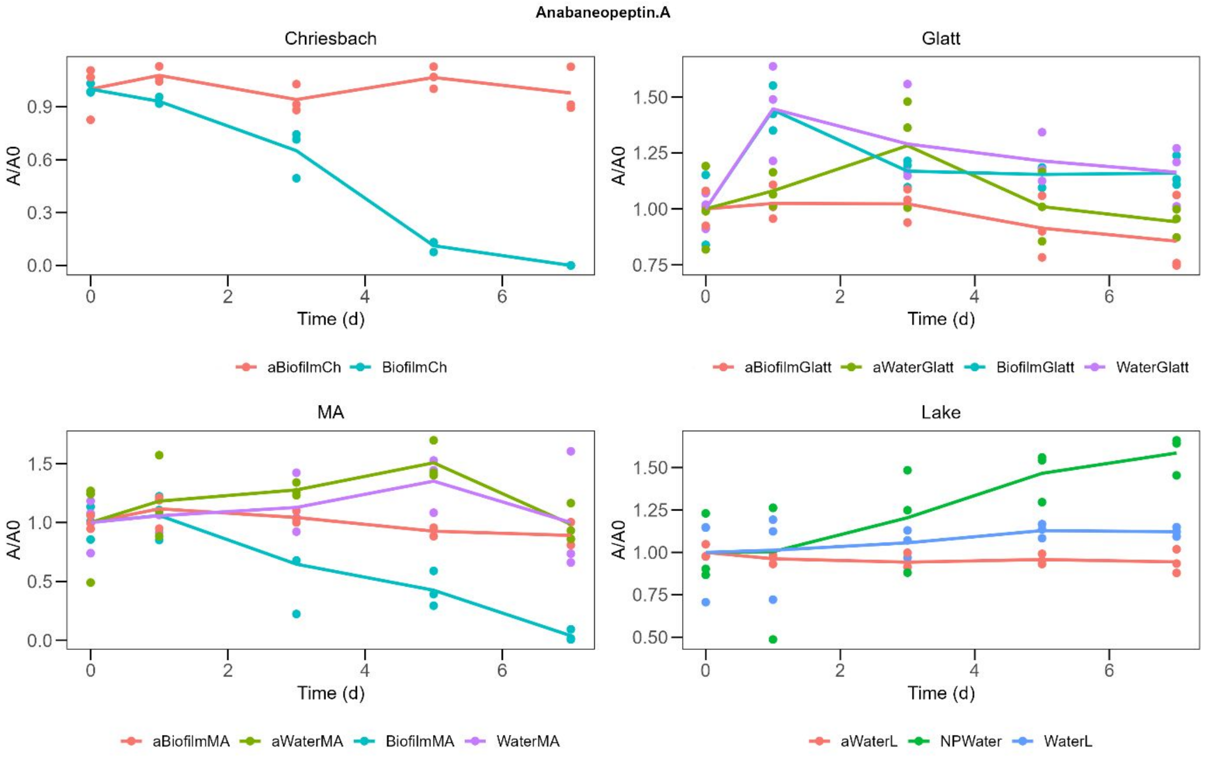

5. Oscillamide Y

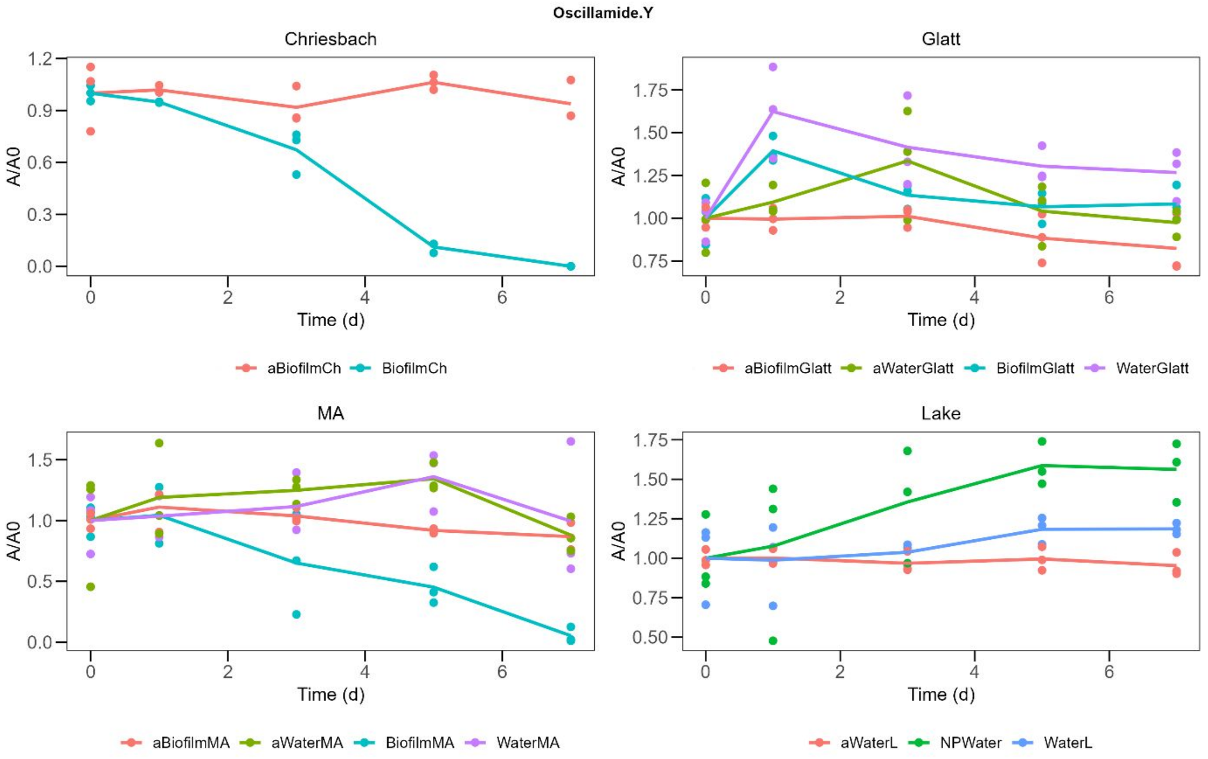

6. Cyanopeptolin 963A

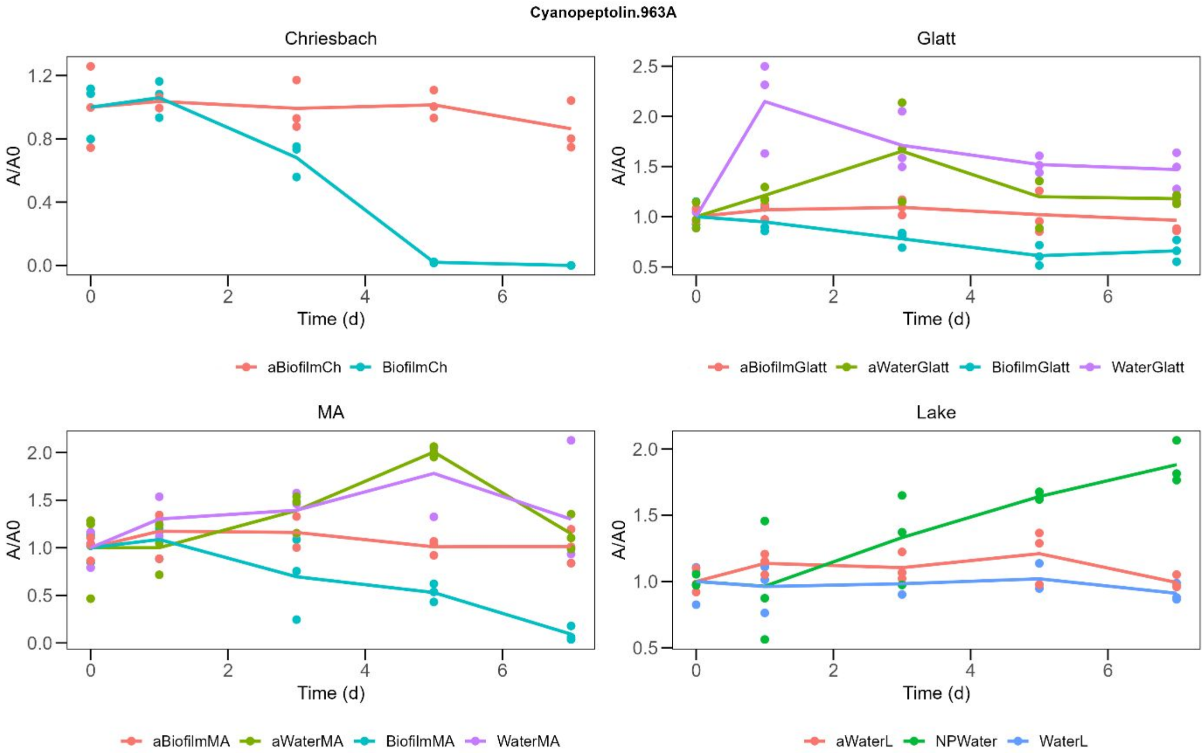

7. Cyanopeptolin 1020

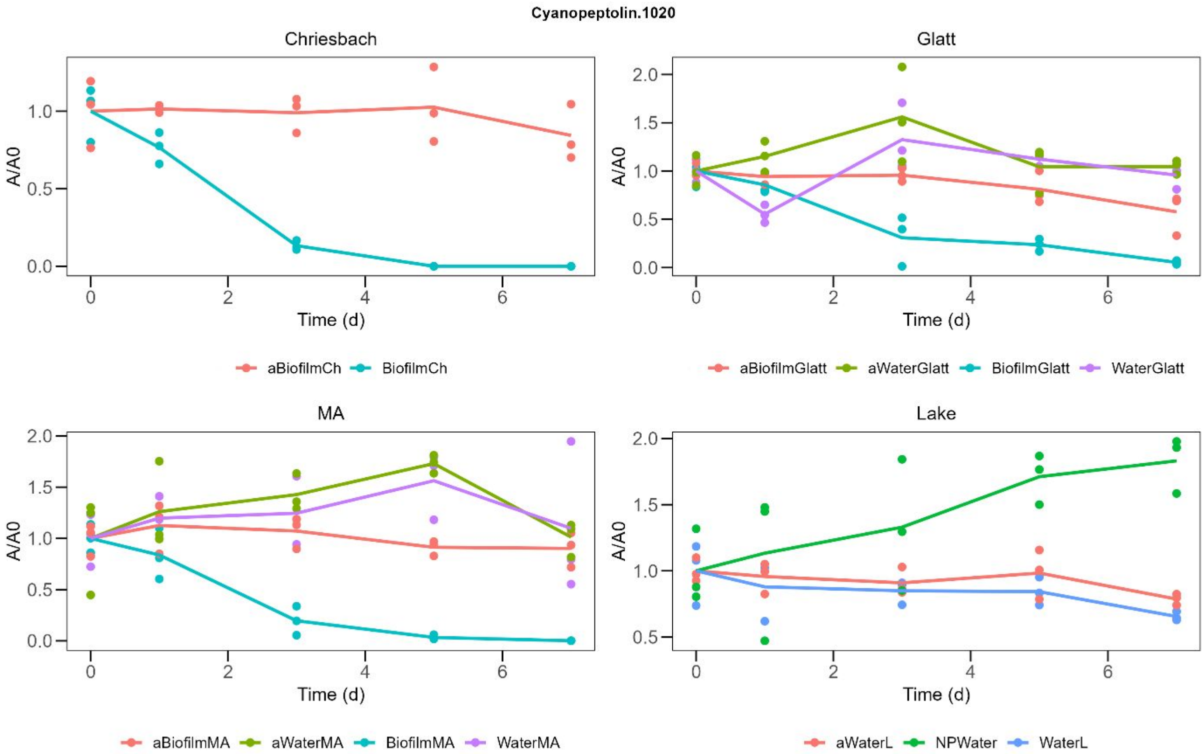

8. Cyanopeptolin A

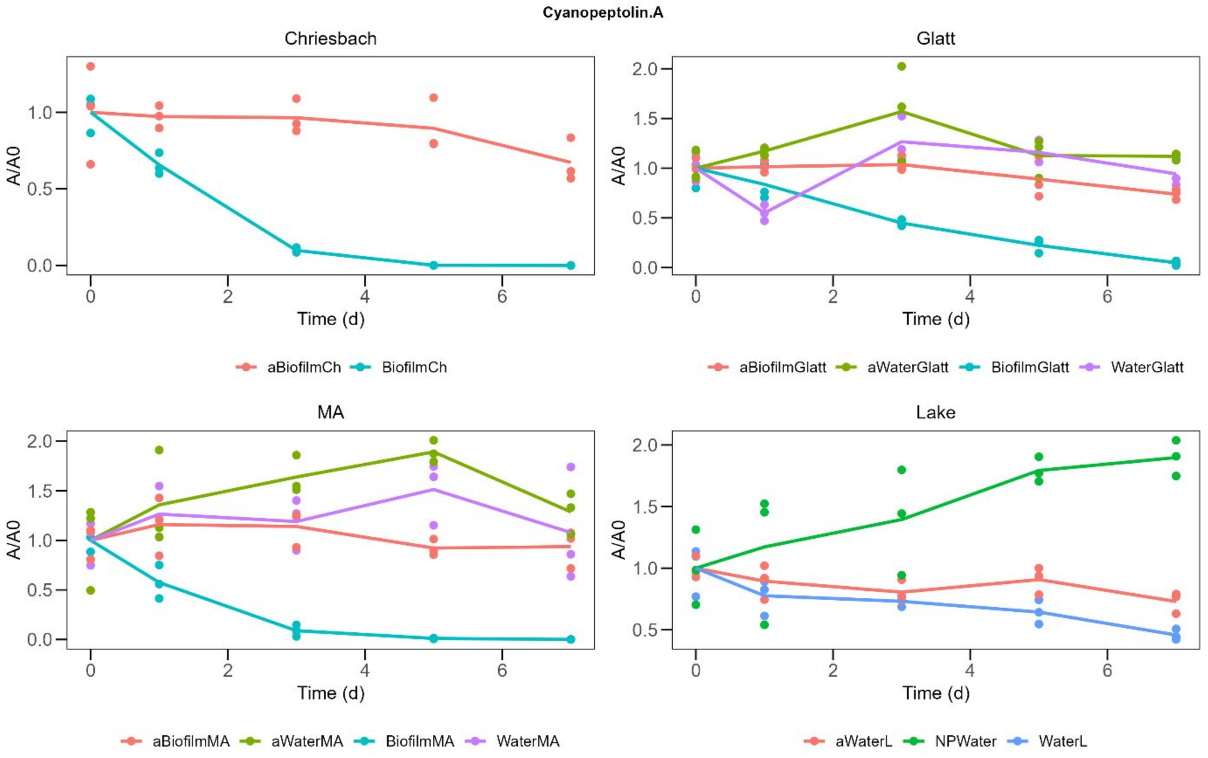

9. Cyanopeptolin B

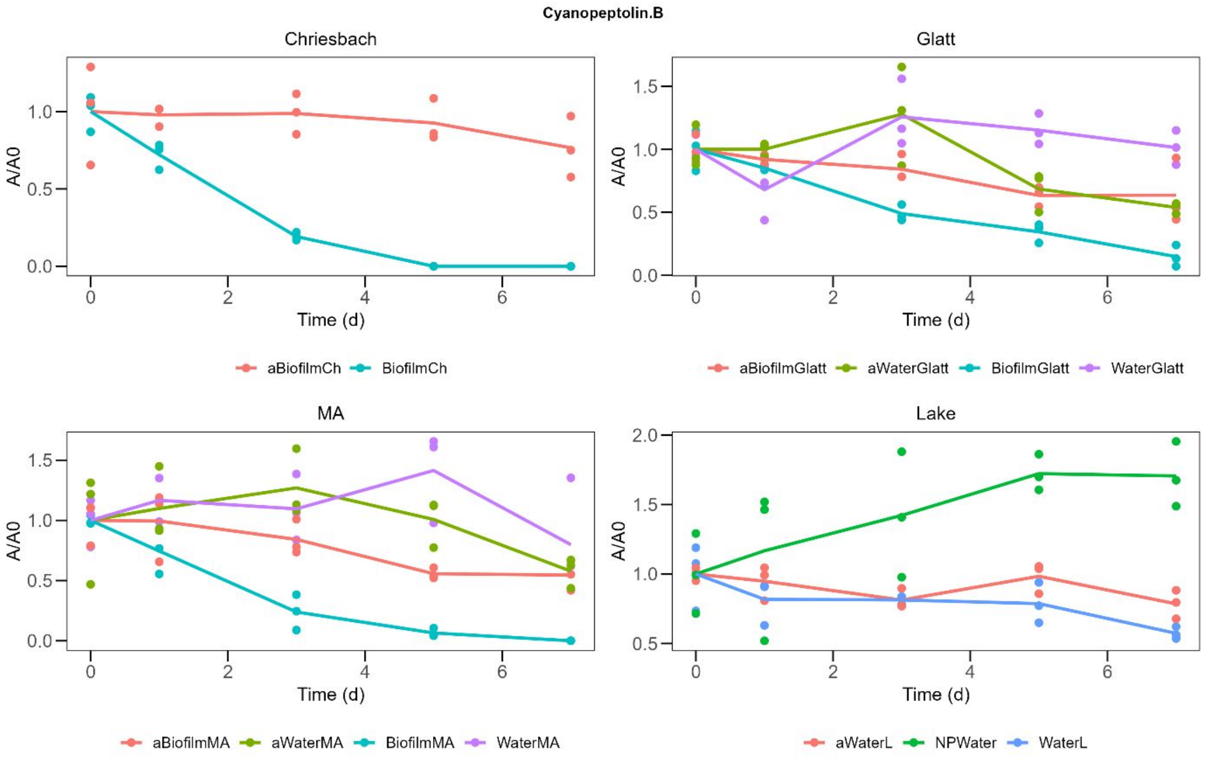

10. Cyanopeptolin C

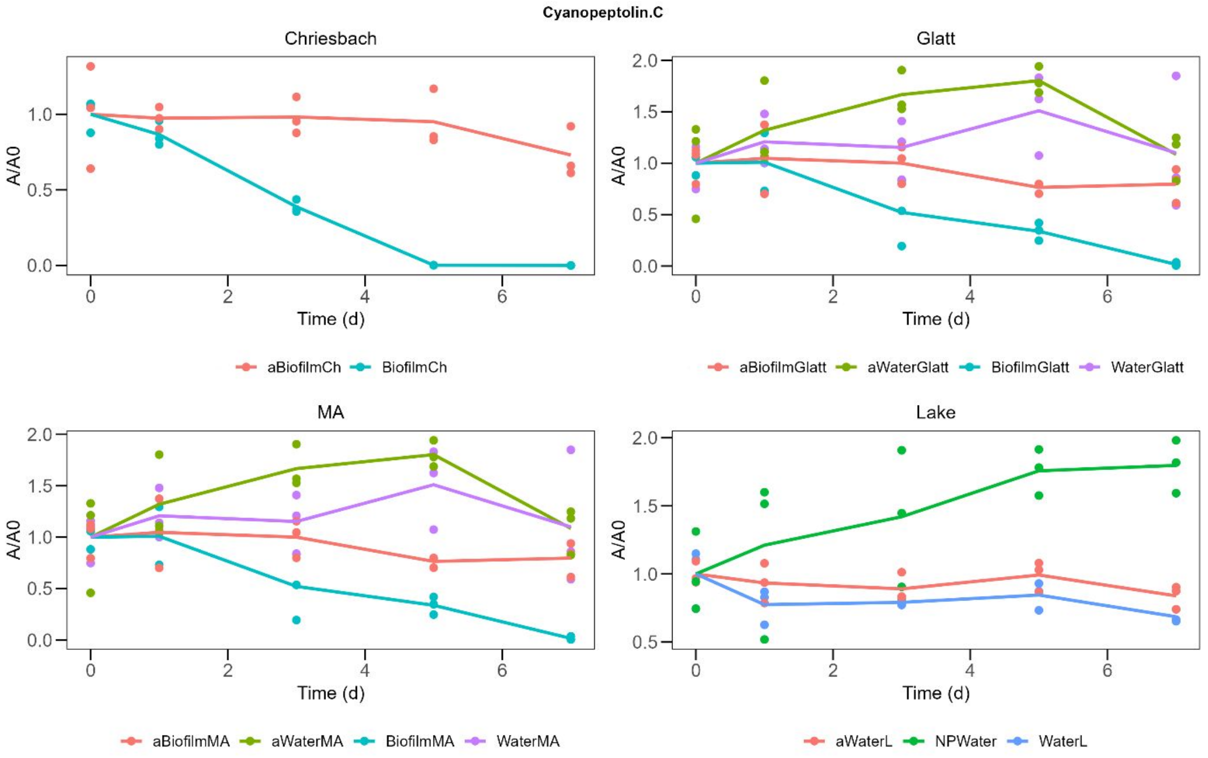

11. Cyanopeptolin D

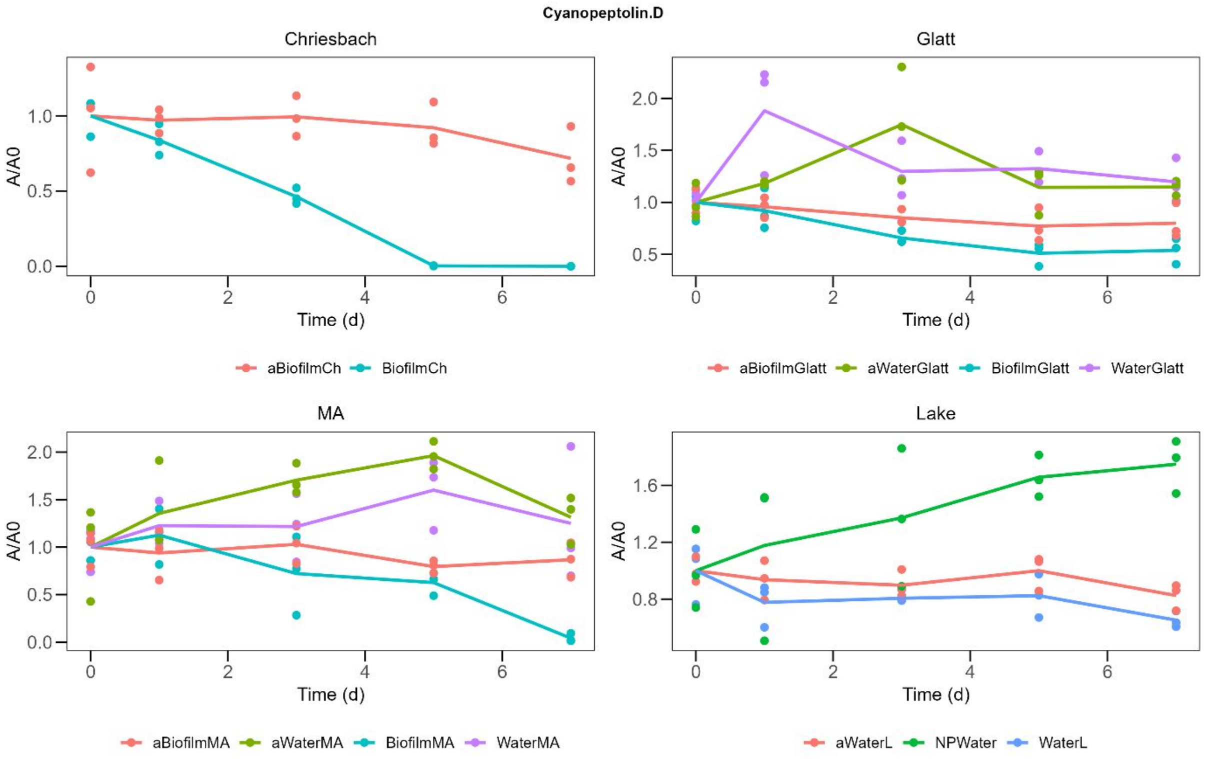

12. MC-HtyR

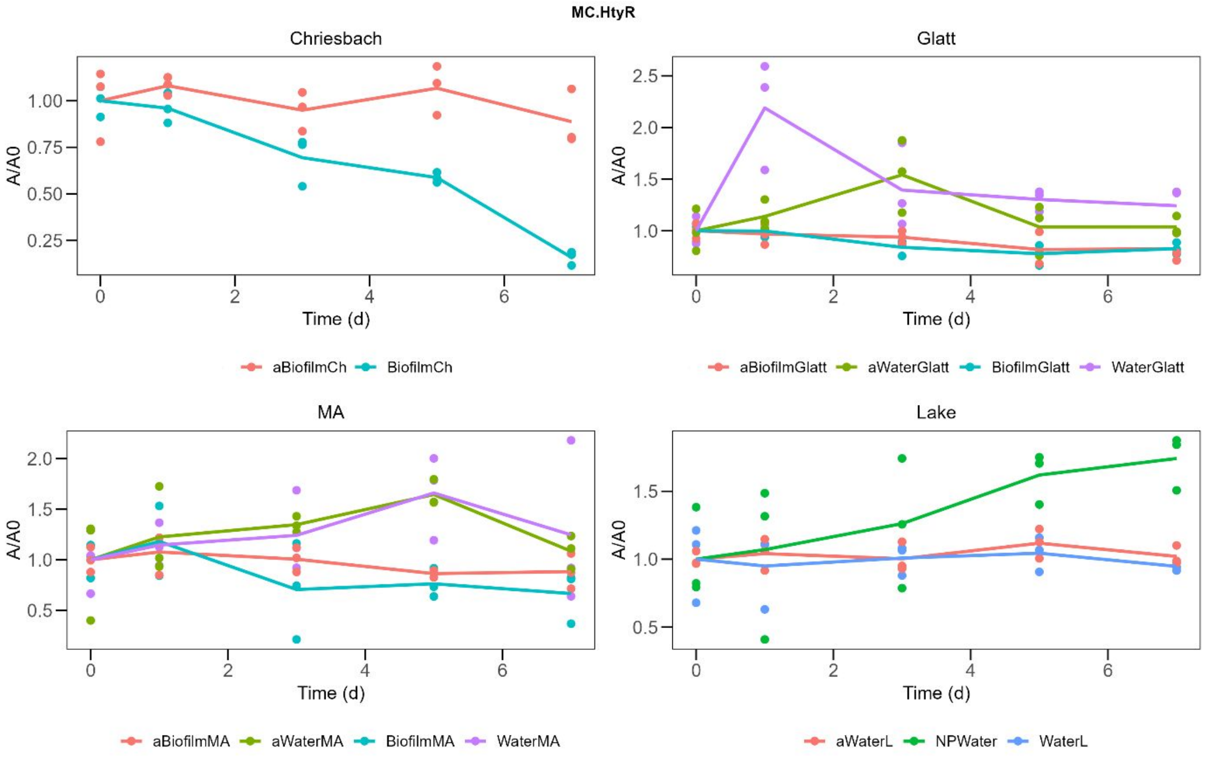

13. MC-LA

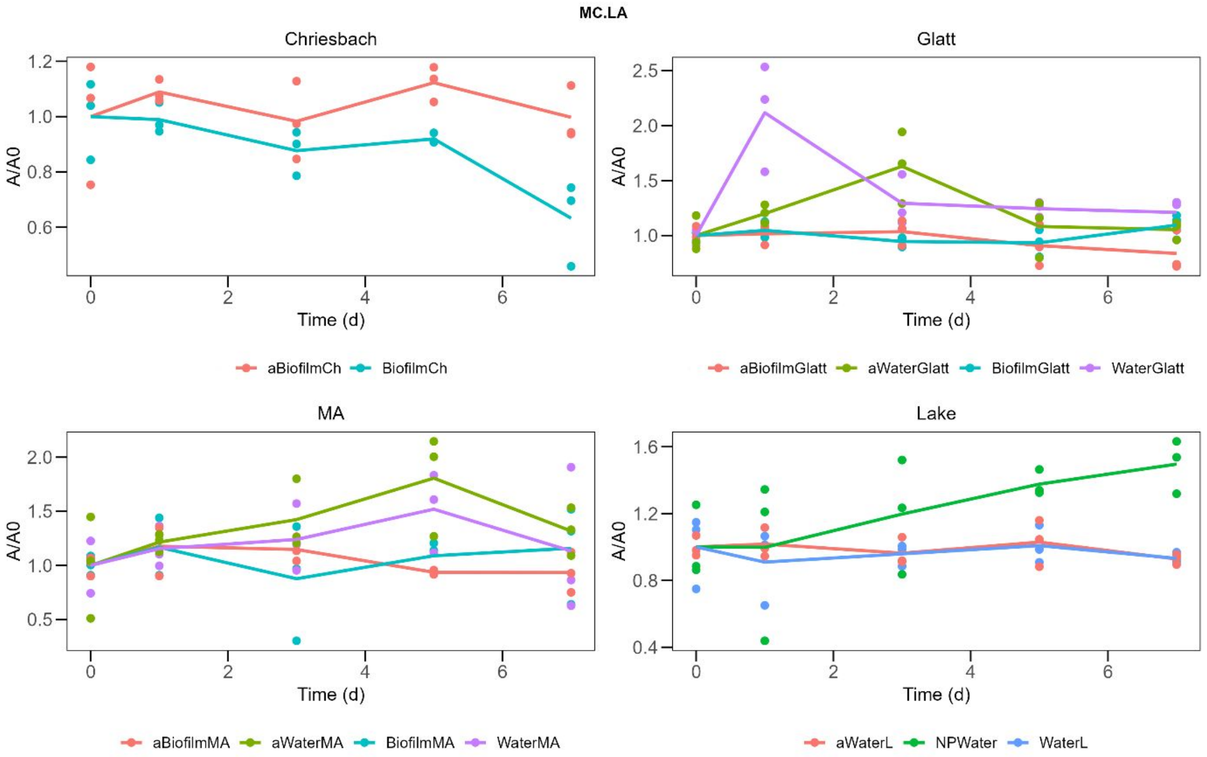

14. MC-LAba

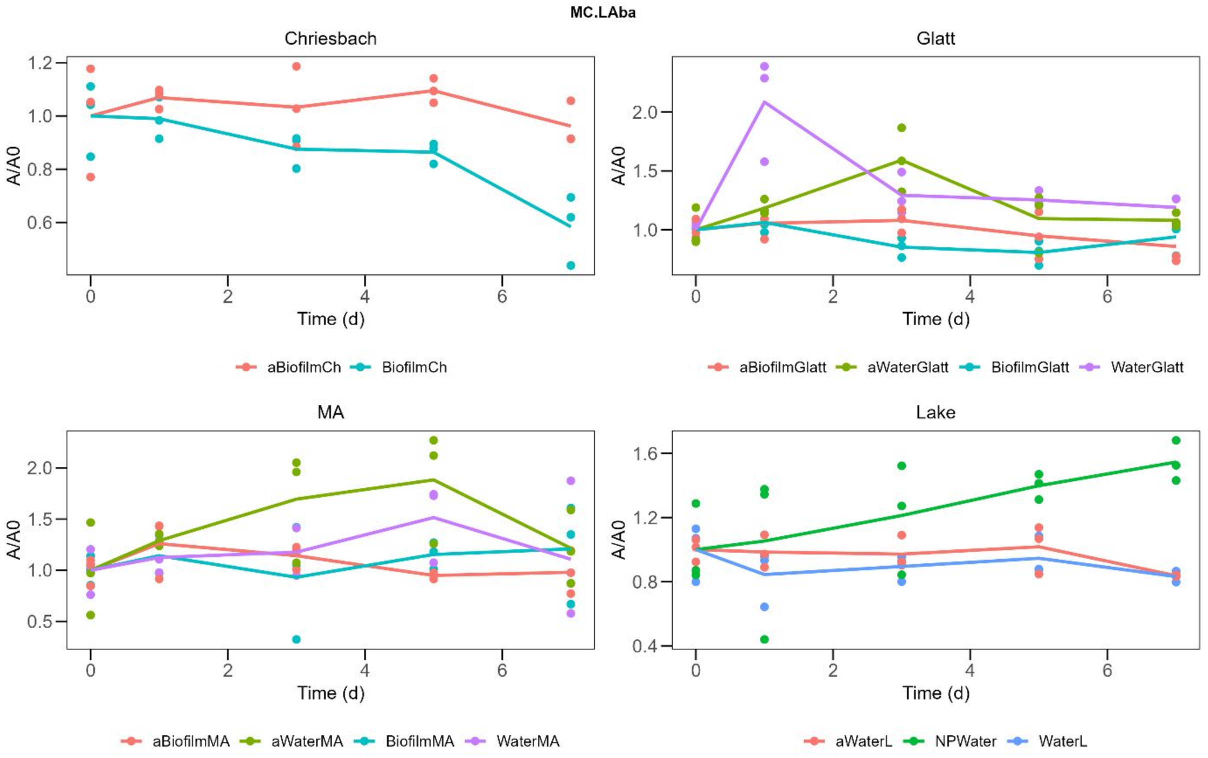

15. MC-LL

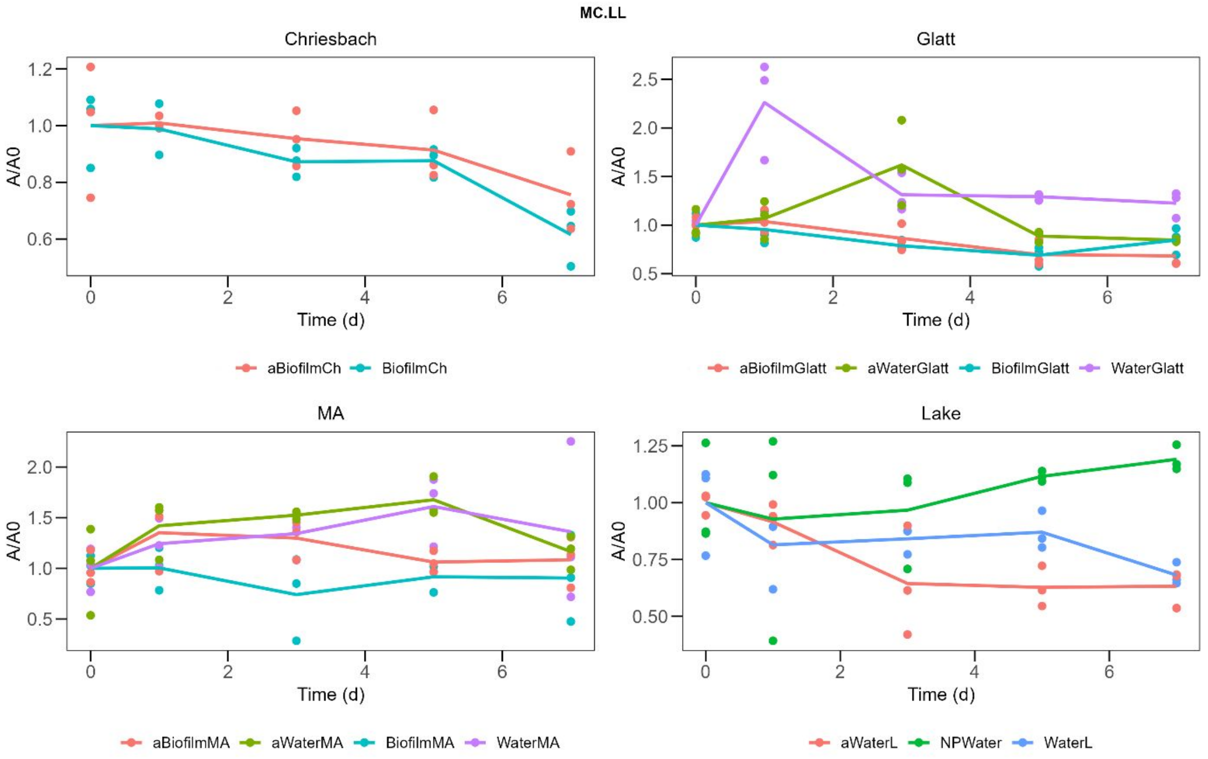

16. MC-LR

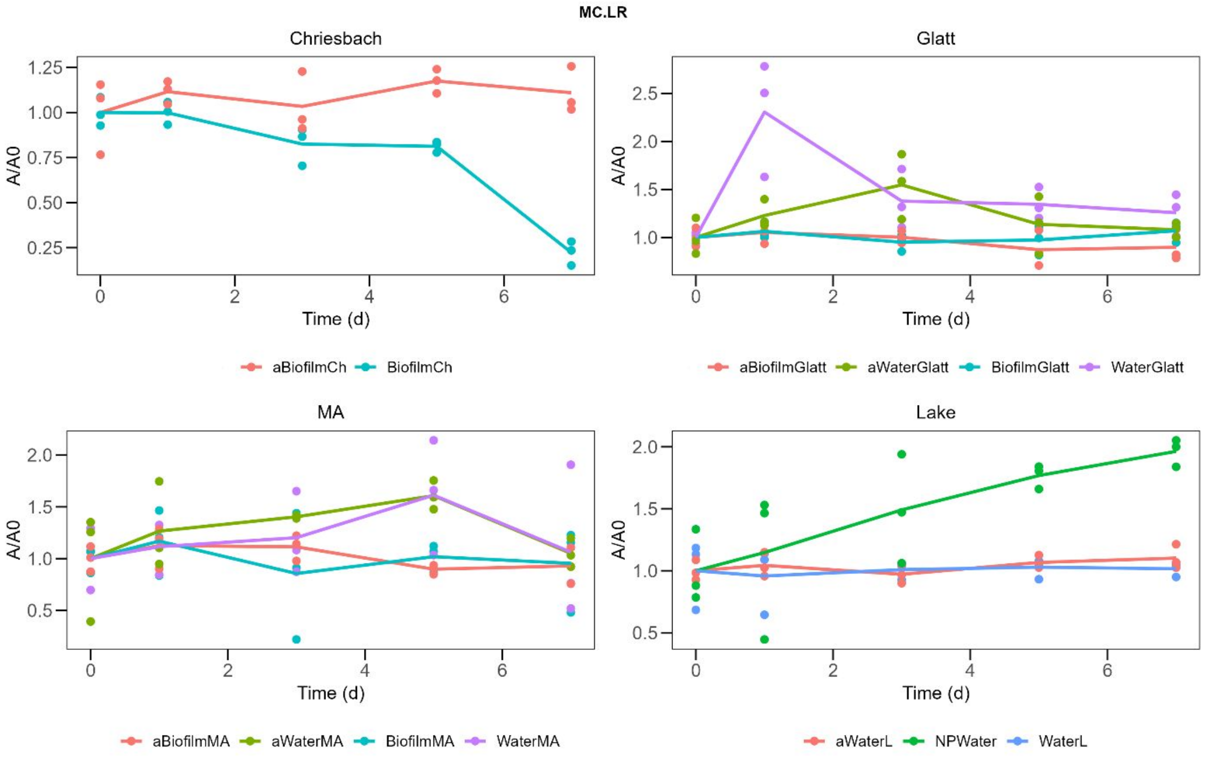

17. MC-YR

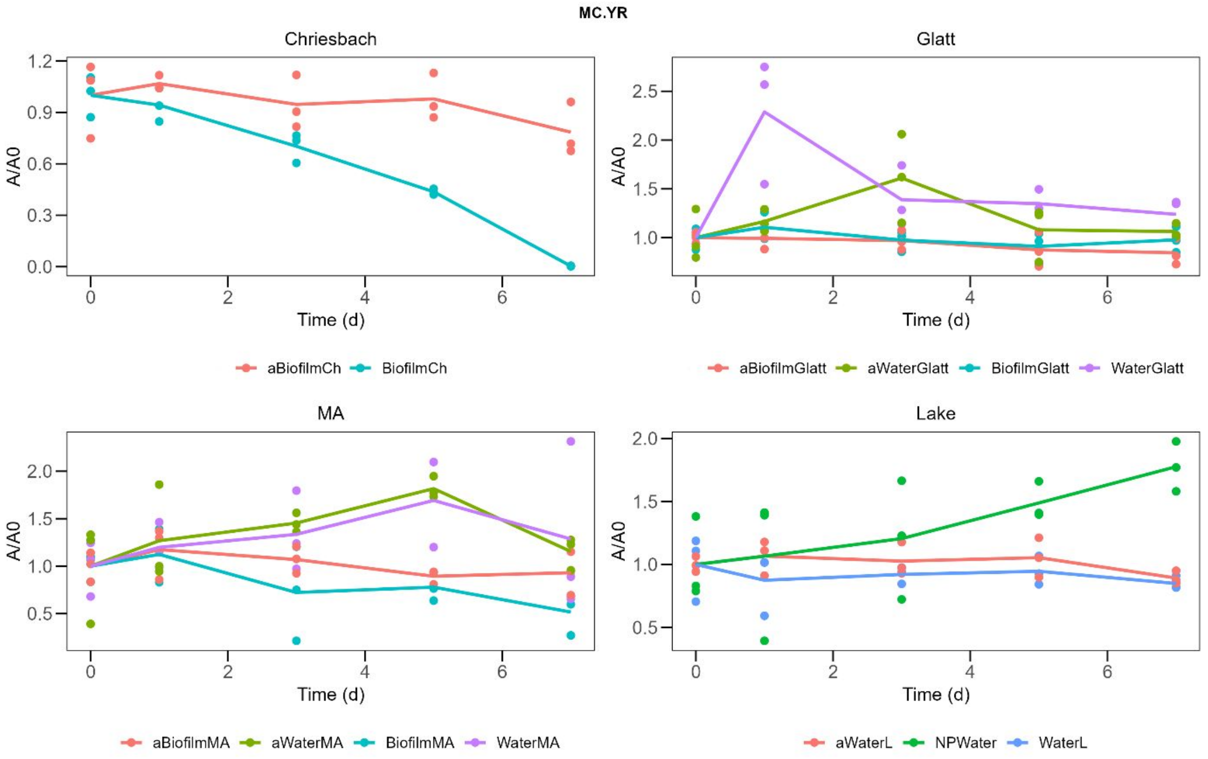

18. [D-Asp^3]MC-(H4)YR

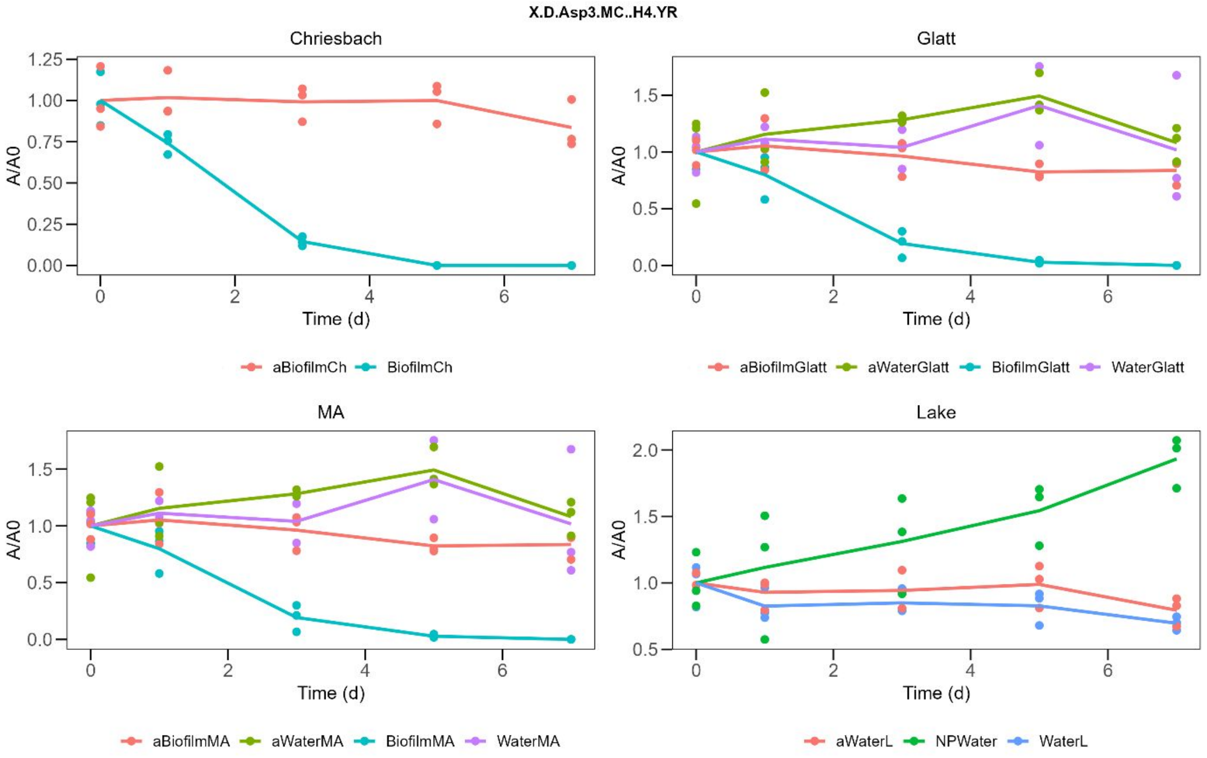

19. [D-Asp^3]MC-LA

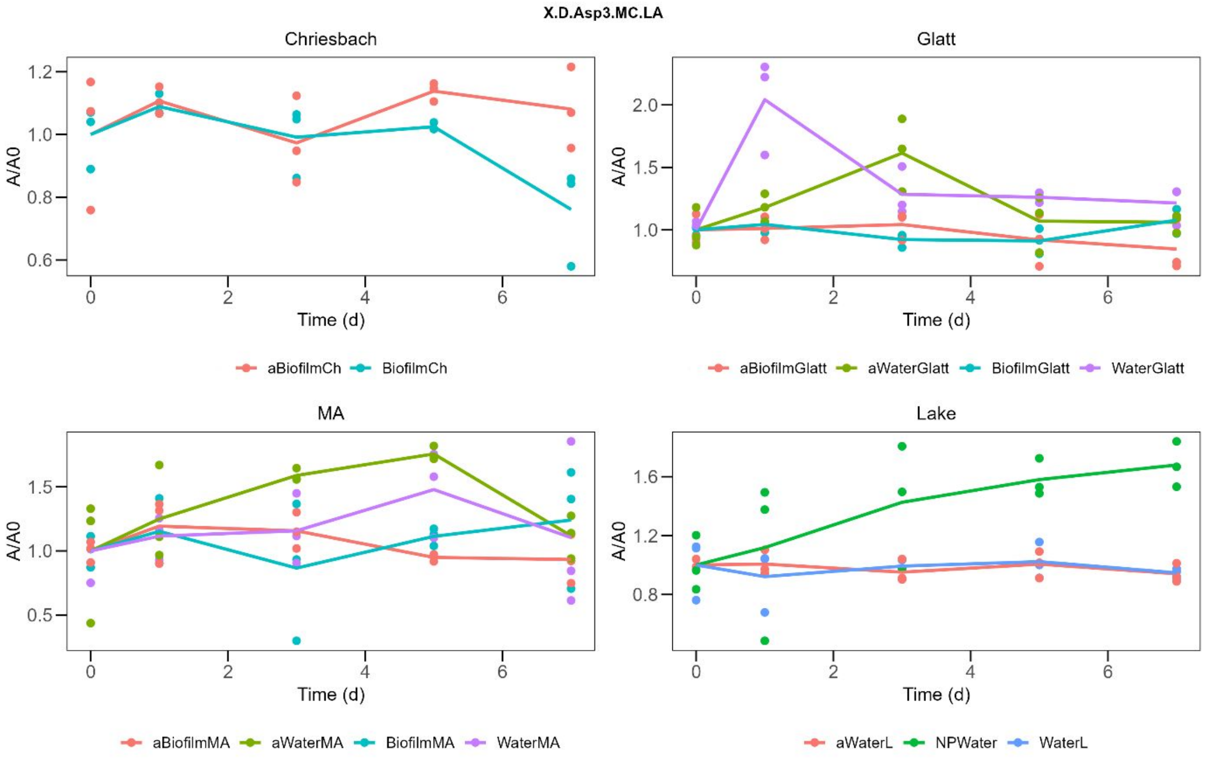

20. [D-Asp^3]MC-LR

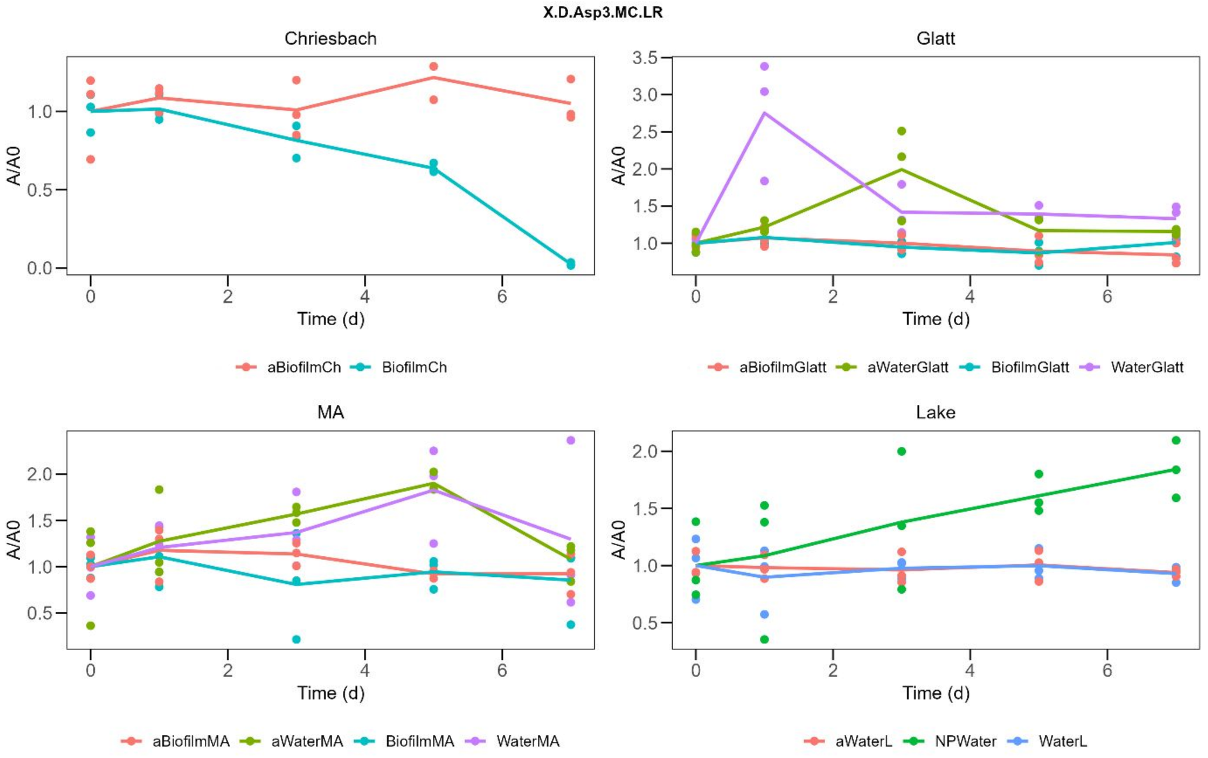

21. [D-Asp^3, (*E*)Dhb^7]MC-RR

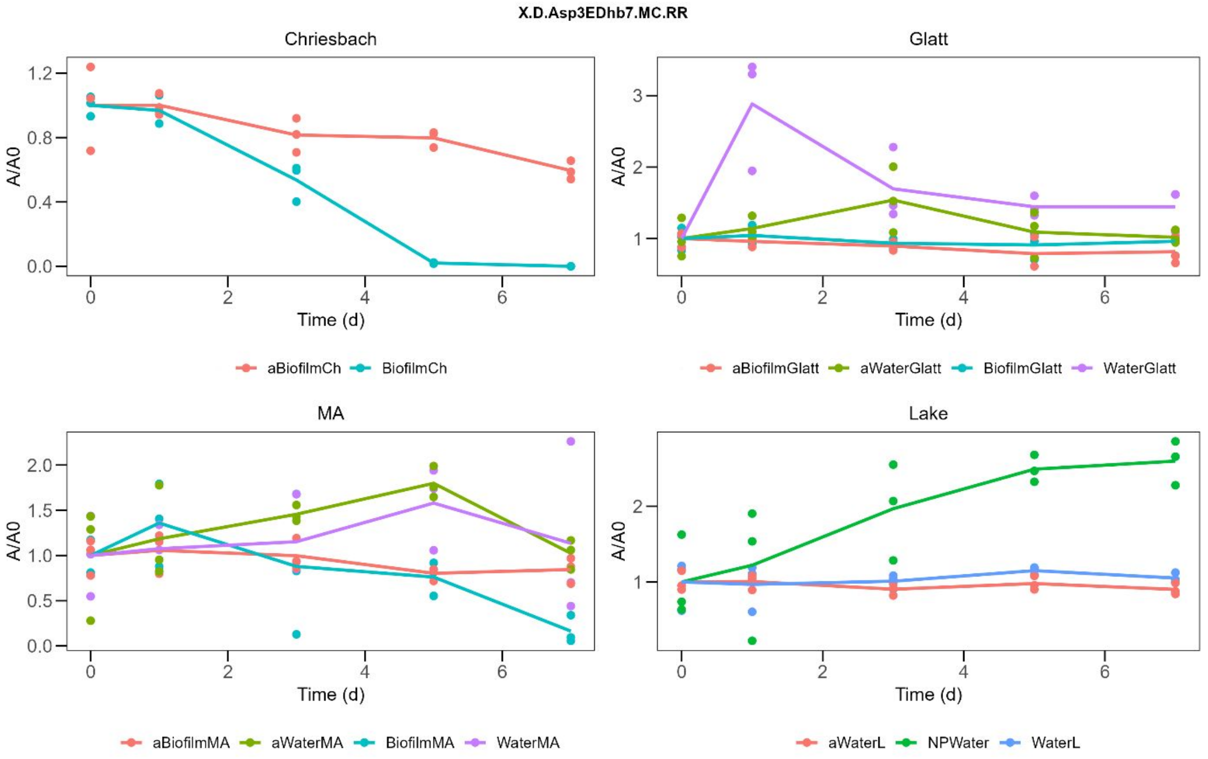

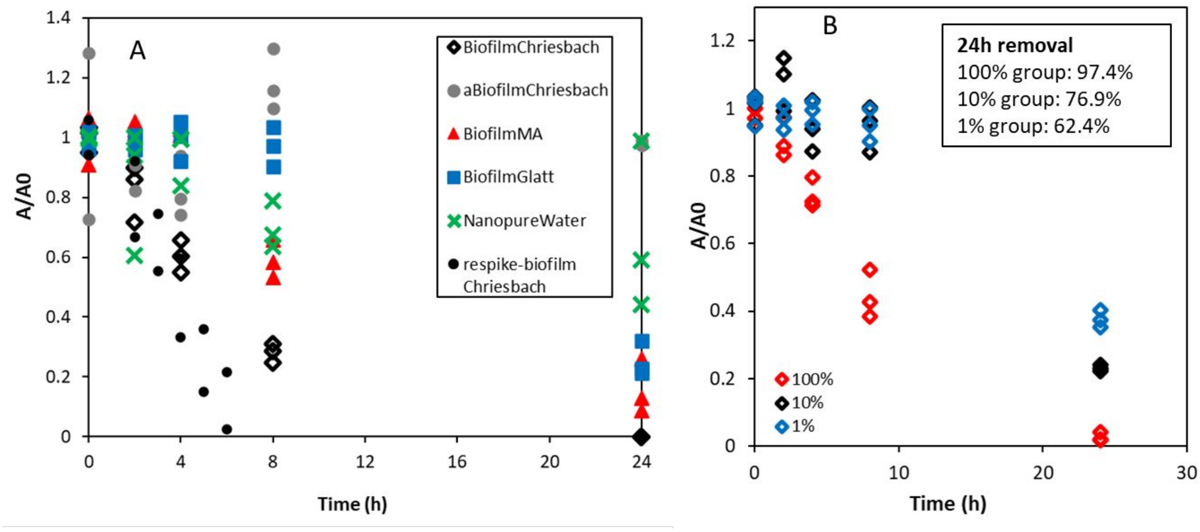

Figure S3. Biotransformation of benzoic acid (BZA) in panel A: biofilm suspensions from River Chriesbach, River Glatt and River Mönschaltorfer Aa (MA), as well as in autoclaved biofilm suspensions from River Chriesbach (aBiofilmChriesbach), in nanopure water and respiked BZA after 24 h to Chriesbach biofilm, showing the detected peak area normalized to the initial peak area across the incubation time; panel B: in 100%, 10% and 1% biofilm suspension groups and 24 h removal.

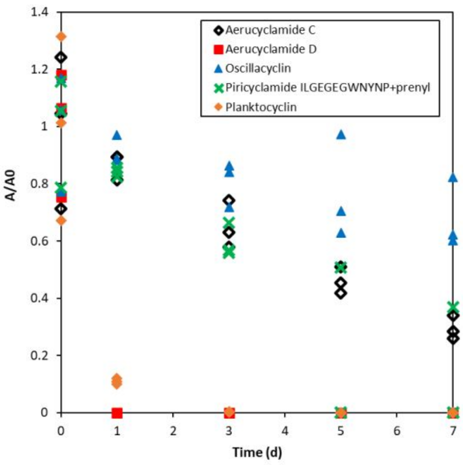

Figure S4. Cyanopeptides that showed removal in abiotic controls (autoclaved biofilm suspension), for example with 32% for oscillacyclin and >70% for other metabolites after 7-day exposure, showing the detected peak area normalized to the initial peak area across the incubation time.

Figure S5 (continuing until page S29). Biotransformation kinetics of 21 cyanopeptides in diluted biofilm suspension (100, 10, 1%) and dilute initial metabolite mixtures (100, 10, 2%), showing the detected peak area normalized to the initial peak area across the incubation time.

1. Aerucyclamide A

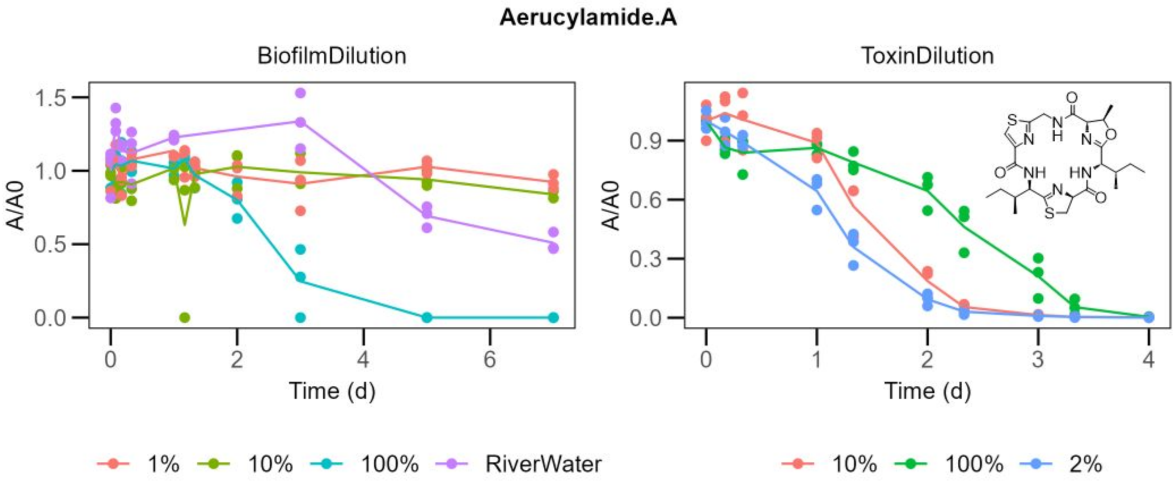

2. Anabaenopeptin B

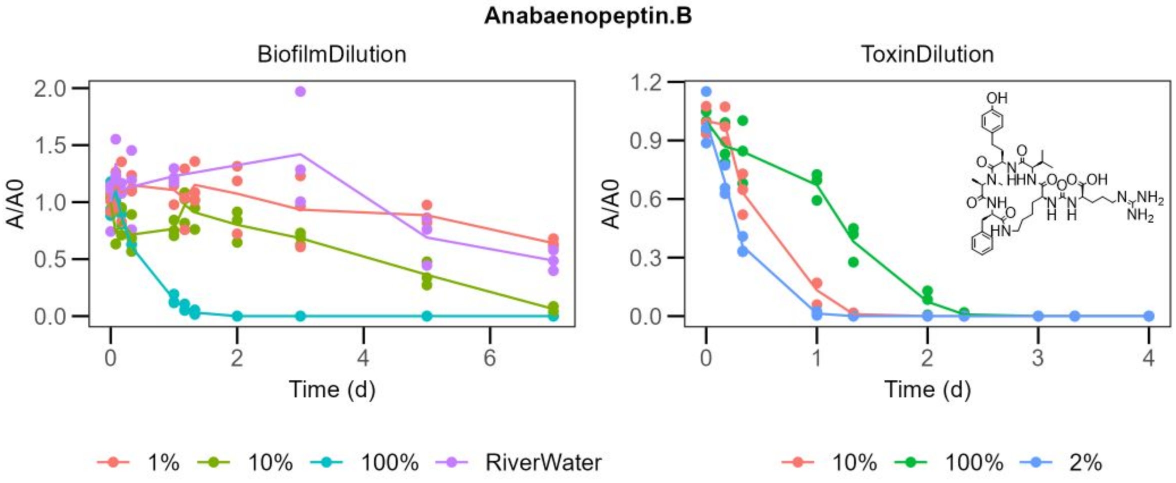

3. Anabaenopeptin A

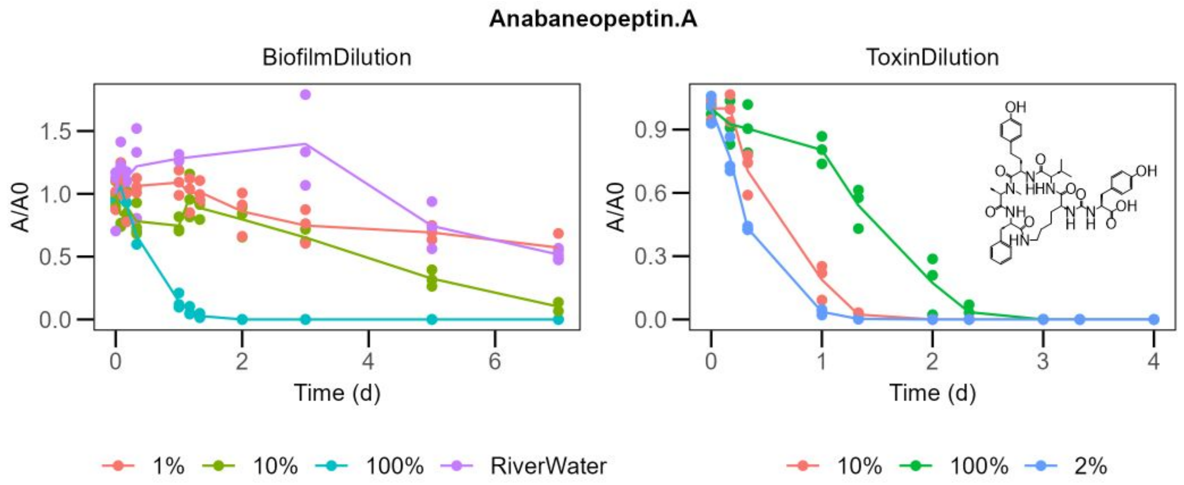

4. Anabaenopeptin F

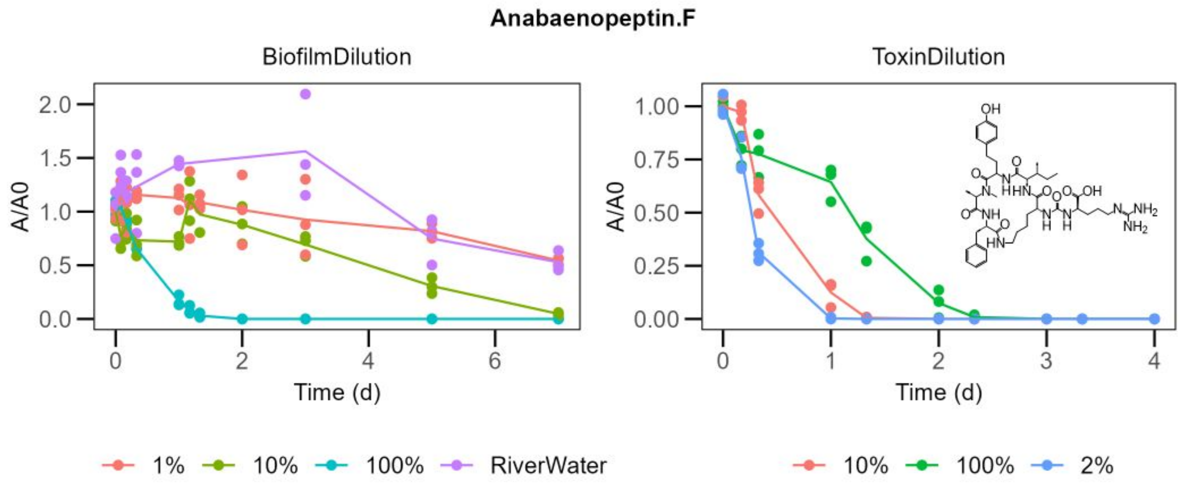

5. Oscillamide Y

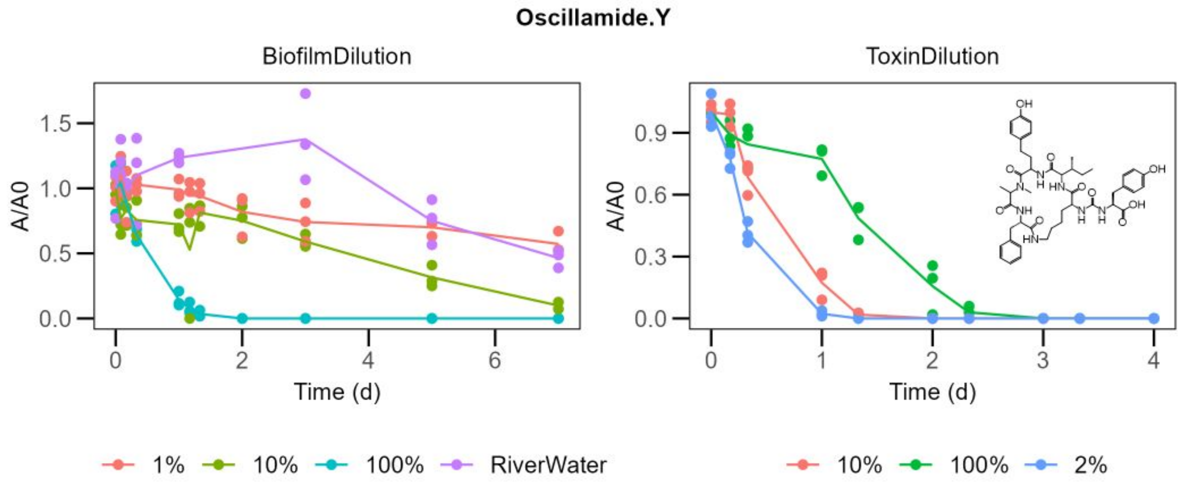

6. Cyanopeptolin 963A

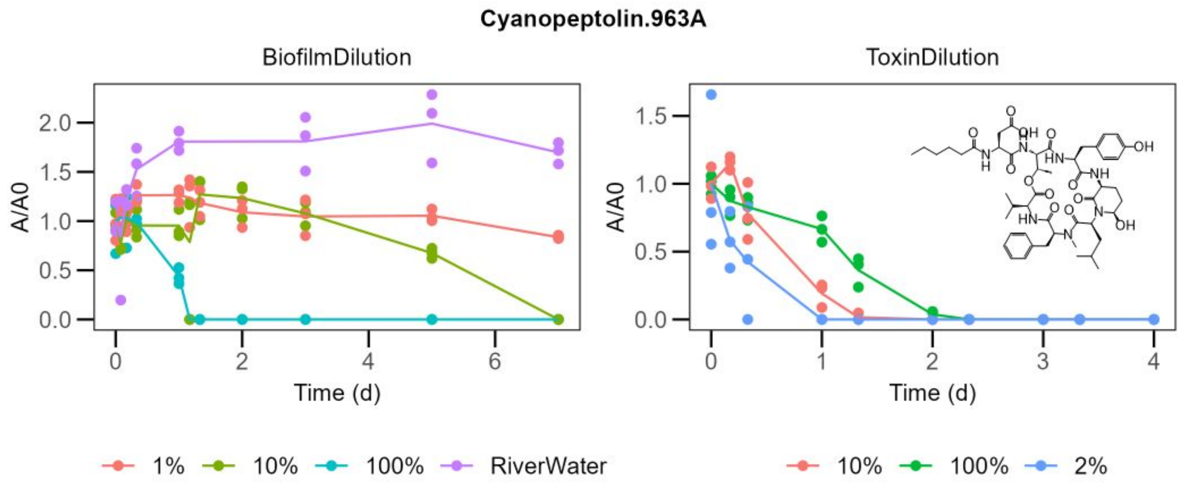

7. Cyanopeptolin 1020

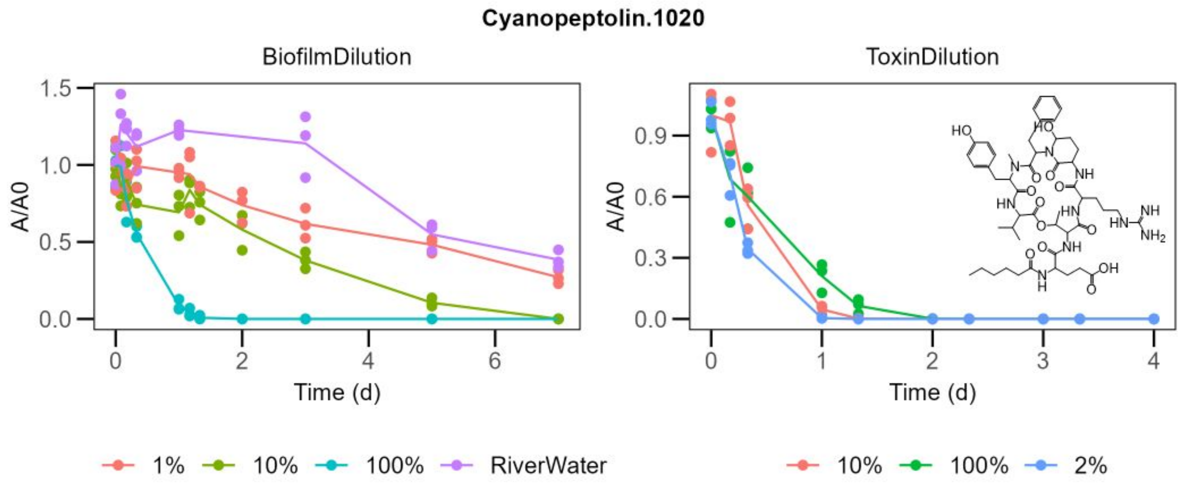

8. Cyanopeptolin A

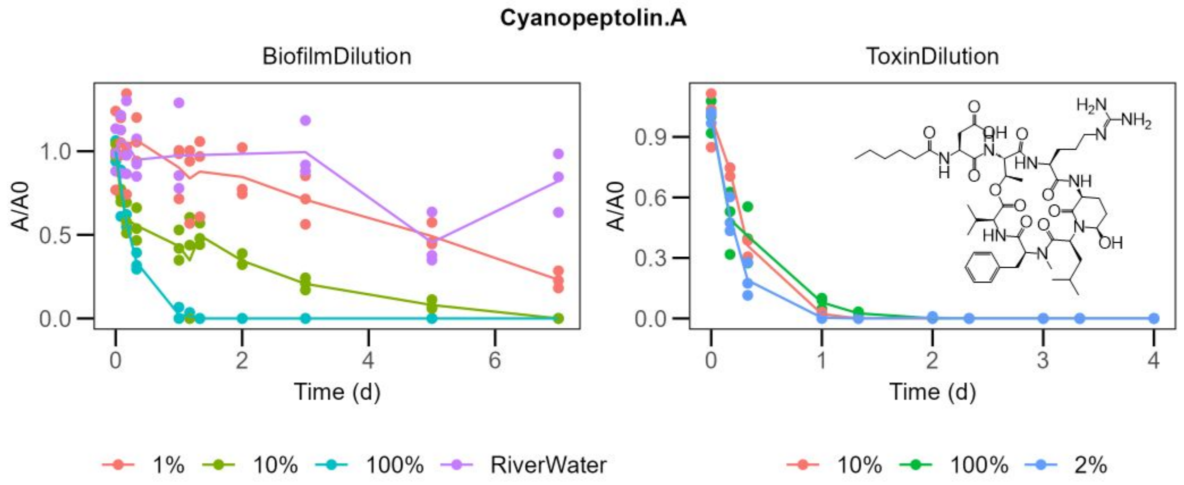

9. Cyanopeptolin B

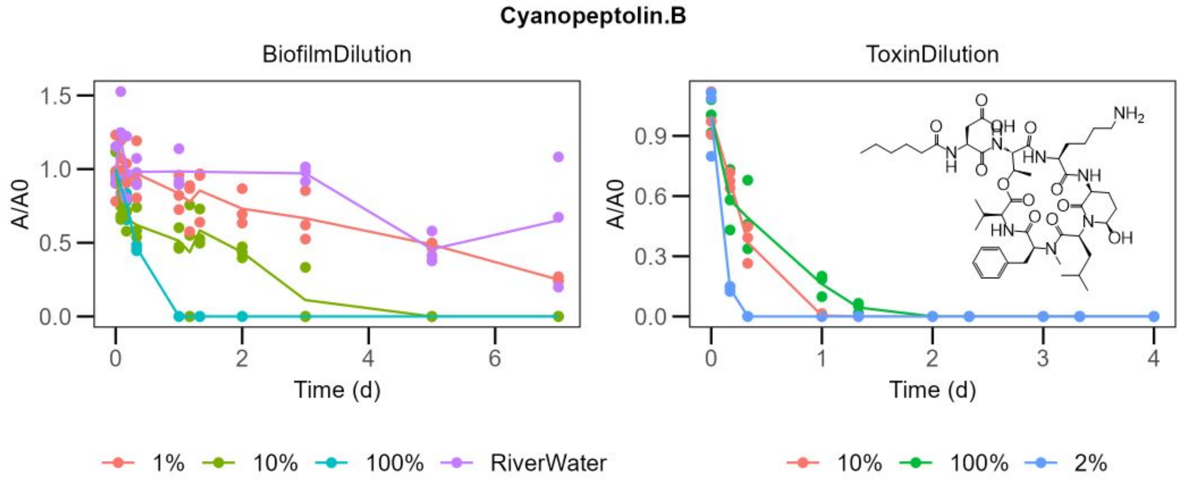

10. Cyanopeptolin C

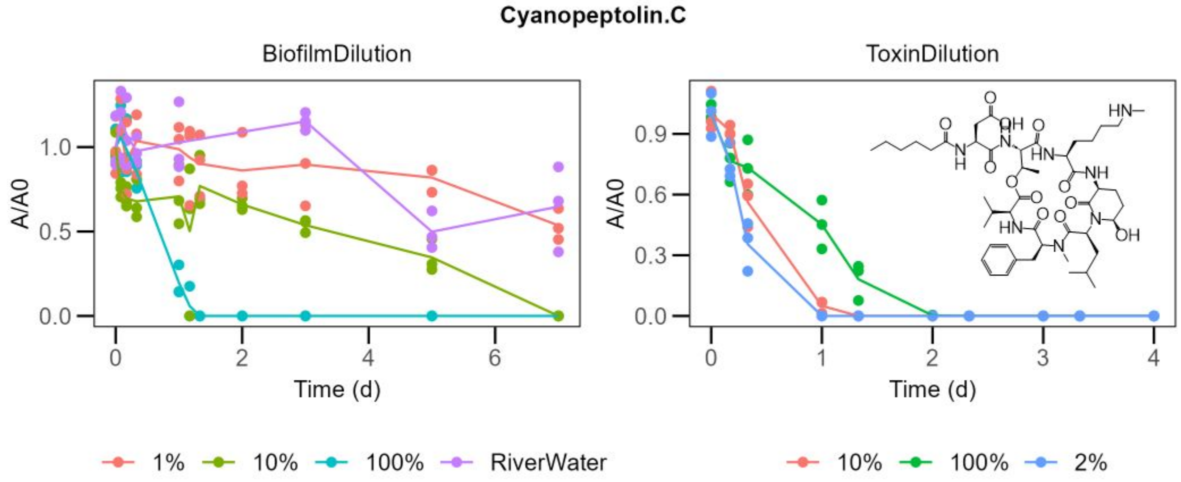

11. Cyanopeptolin D

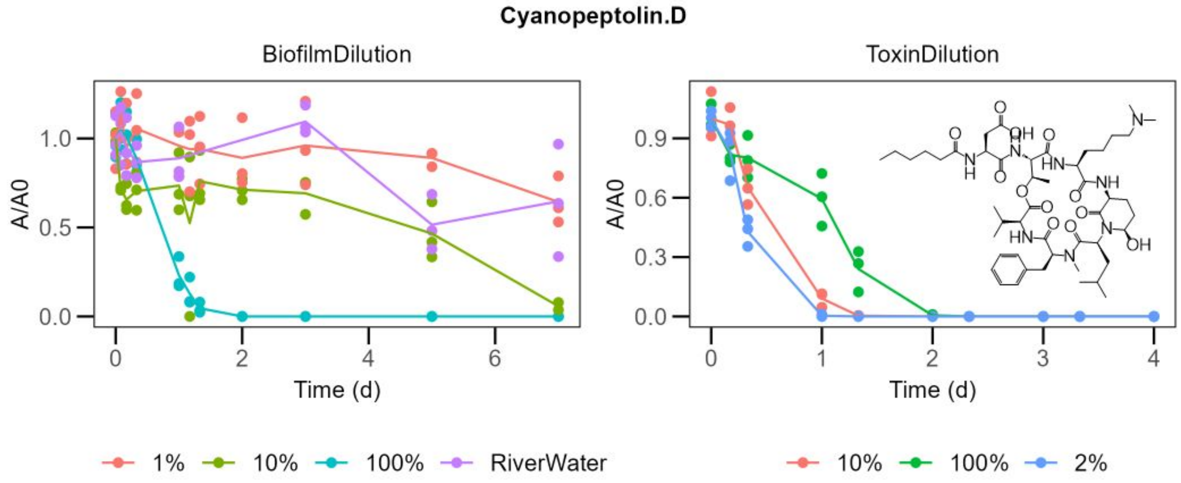

12. MC-HtyR

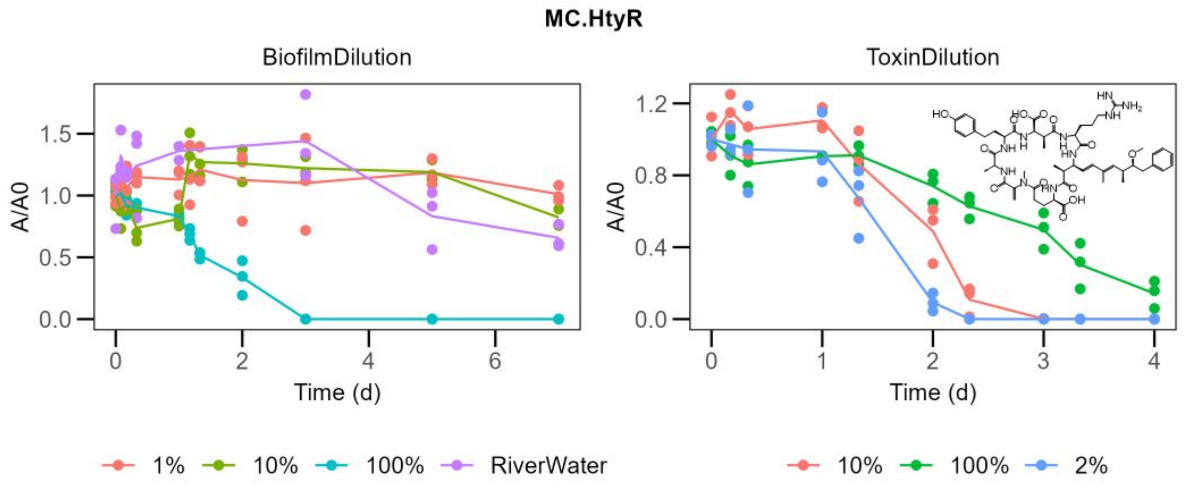

13. MC-LA

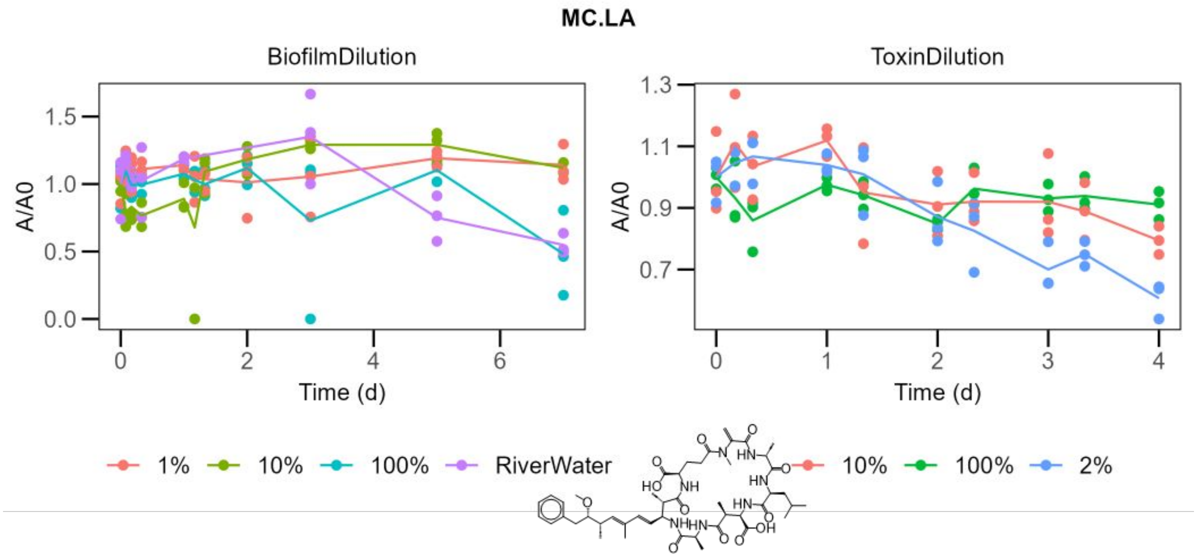

14. MC-LAba

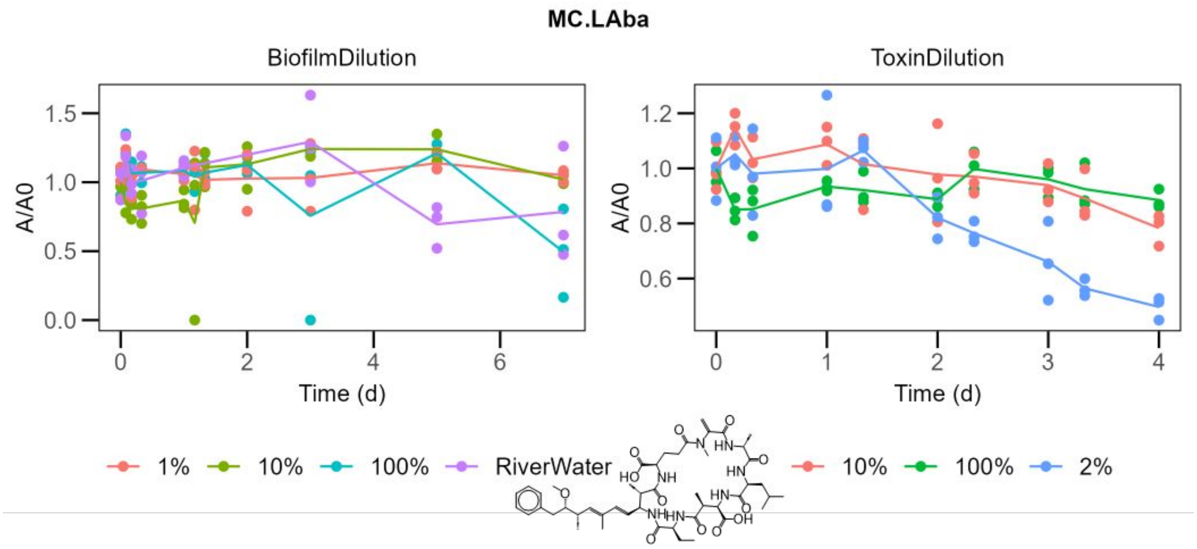

15. MC-LL

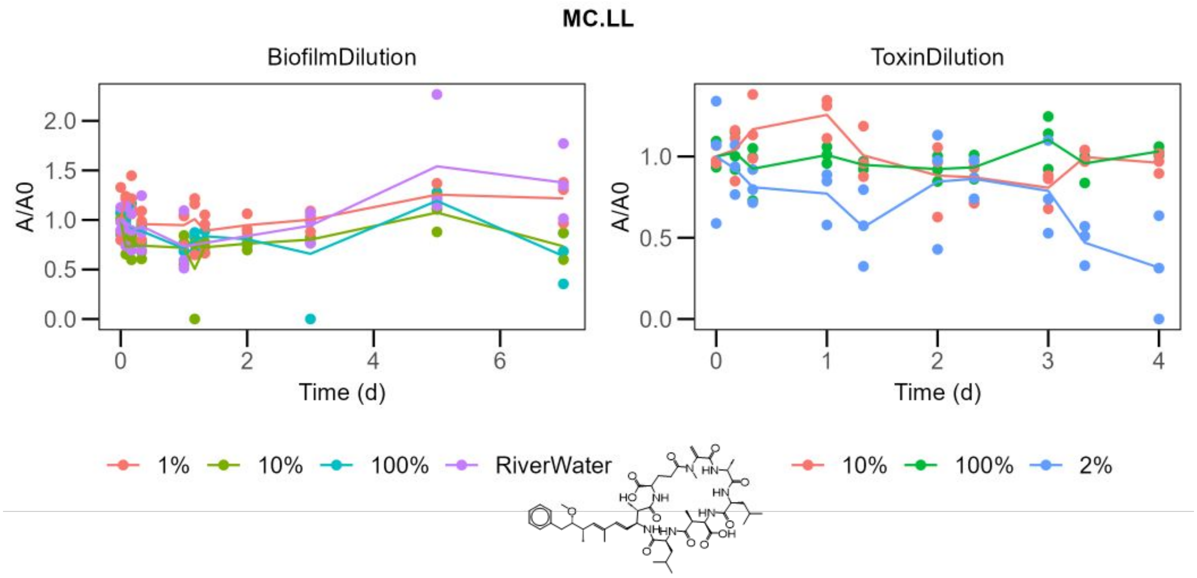

16. MC-LR

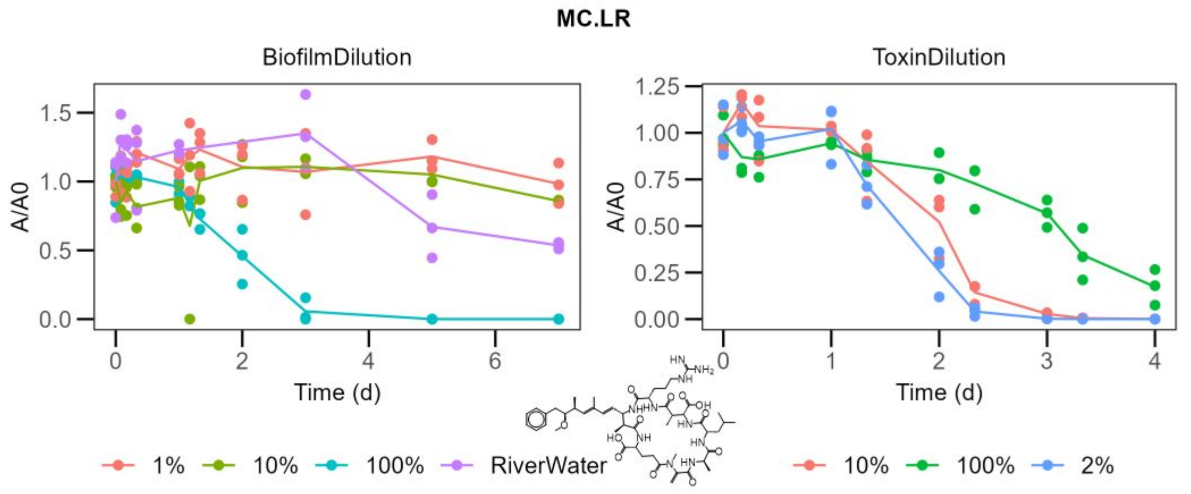

17. MC-YR

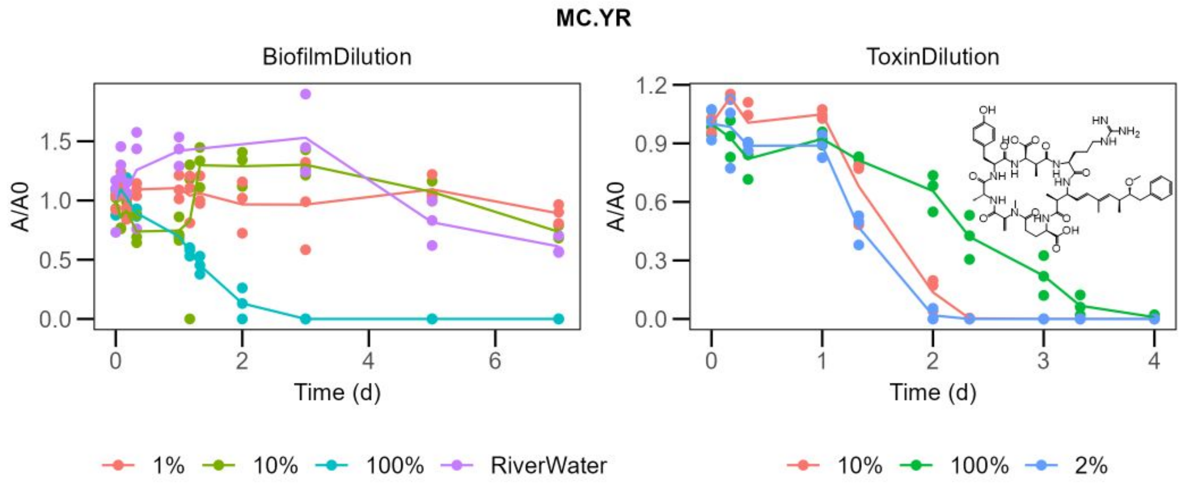

18. [D-Asp^{3}]MC-(H4)YR

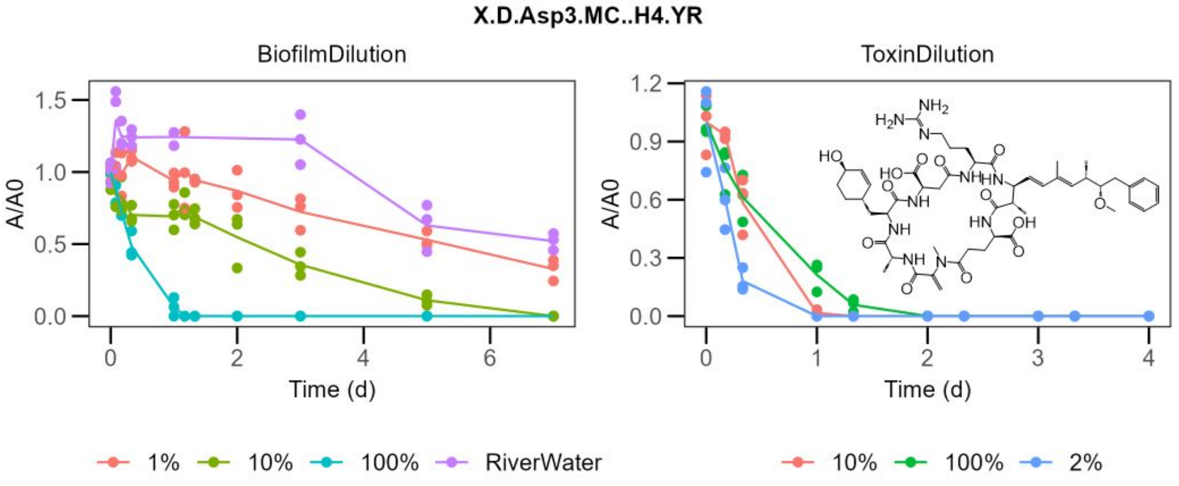

19. [D-Asp^{3}]MC-LA

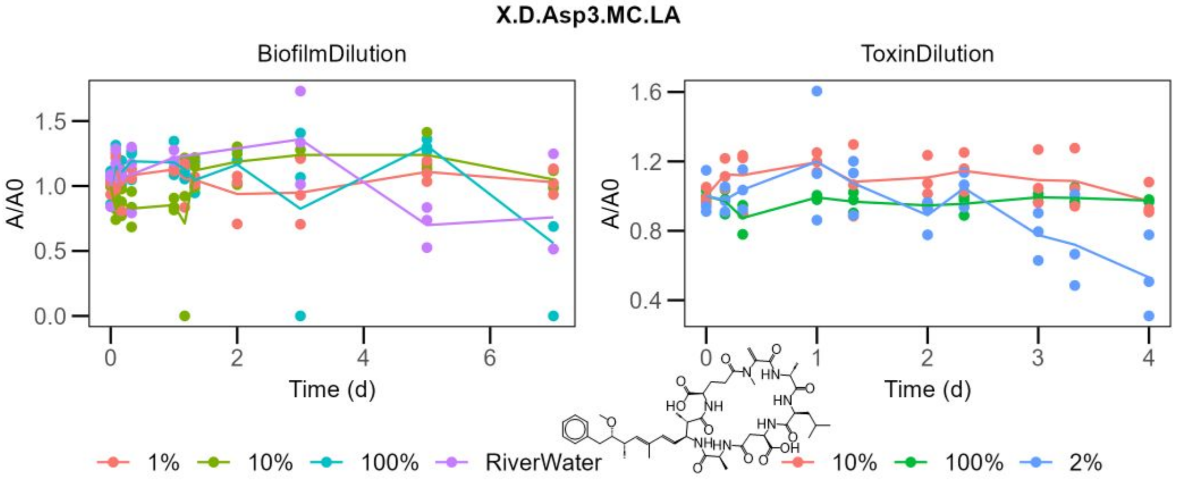

20. [D-Asp^{3}]MC-LR

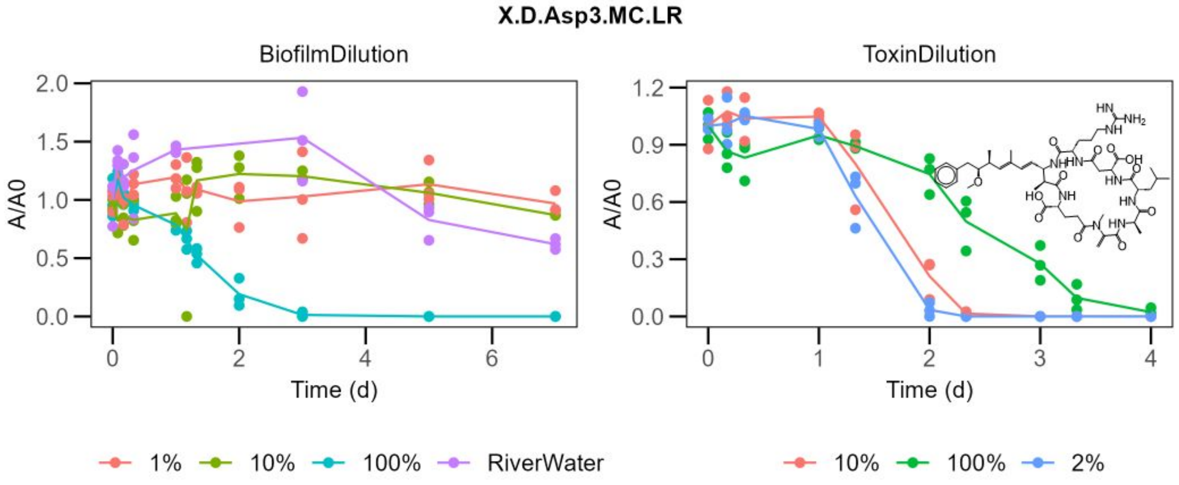

21. [D-Asp^{3}, (*E*)Dhb^{7}]MC-RR

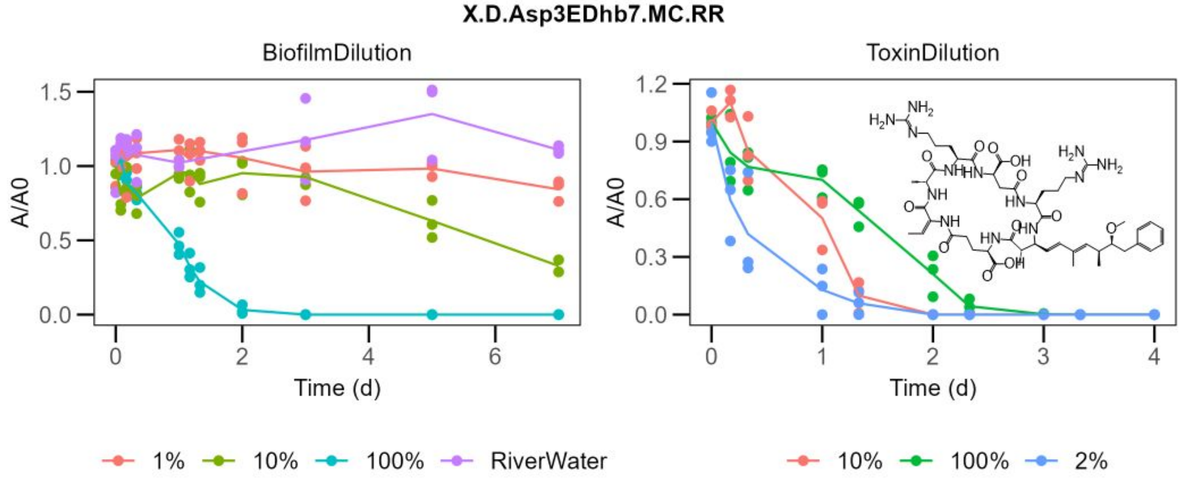

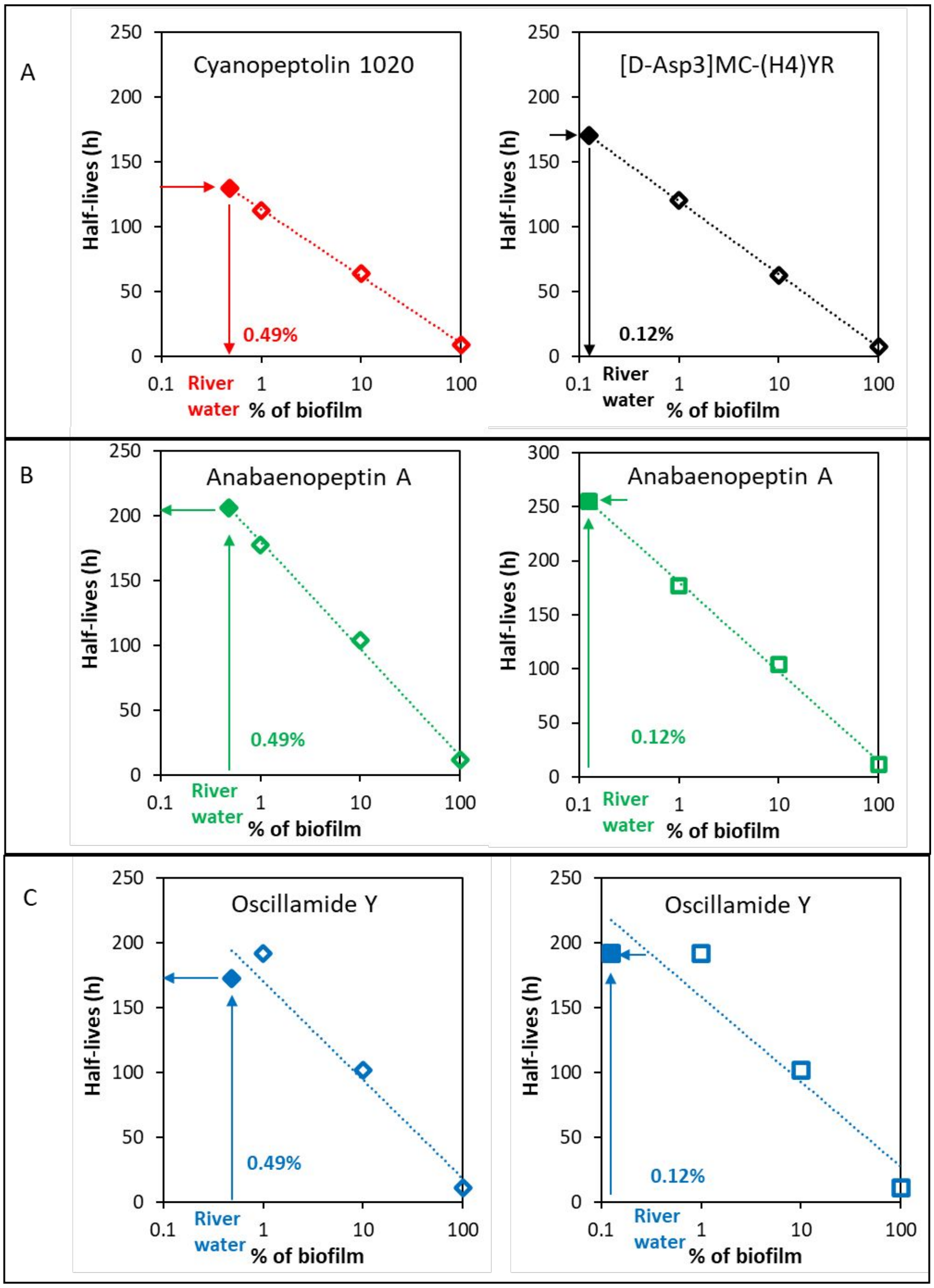

Figure S6. Half-lives of Cyanopeptolin 1020 (red, panel A), [D-Asp3]MC-(H4)YR (black, panel B), anbaenopeptin A (green, panel B) and oscillamide Y (blue, panel C) in different Chriesbach biofilm dilutions (100%, 10%, 1% of biofilm suspension and river water). Note: the half-lives of oscillamide Y and anabaenopeptin A in river waters were calculated with % biofilm equivalent of Cyanopeptolin 1020 and [D-Asp3]MC-(H4)YR, respectively.

Figure S7. Biotransformation kinetics of 9 additional cyanobacterial metabolites, extracted from Microcystis panniformis MIRS-04 and Microcystis aeruginosa NPDC-01 exposed to Chriesbach biofilm suspensions and the respective abiotic controls (aBiofilm), showing the detected peak area normalized to the initial peak area across the incubation time.

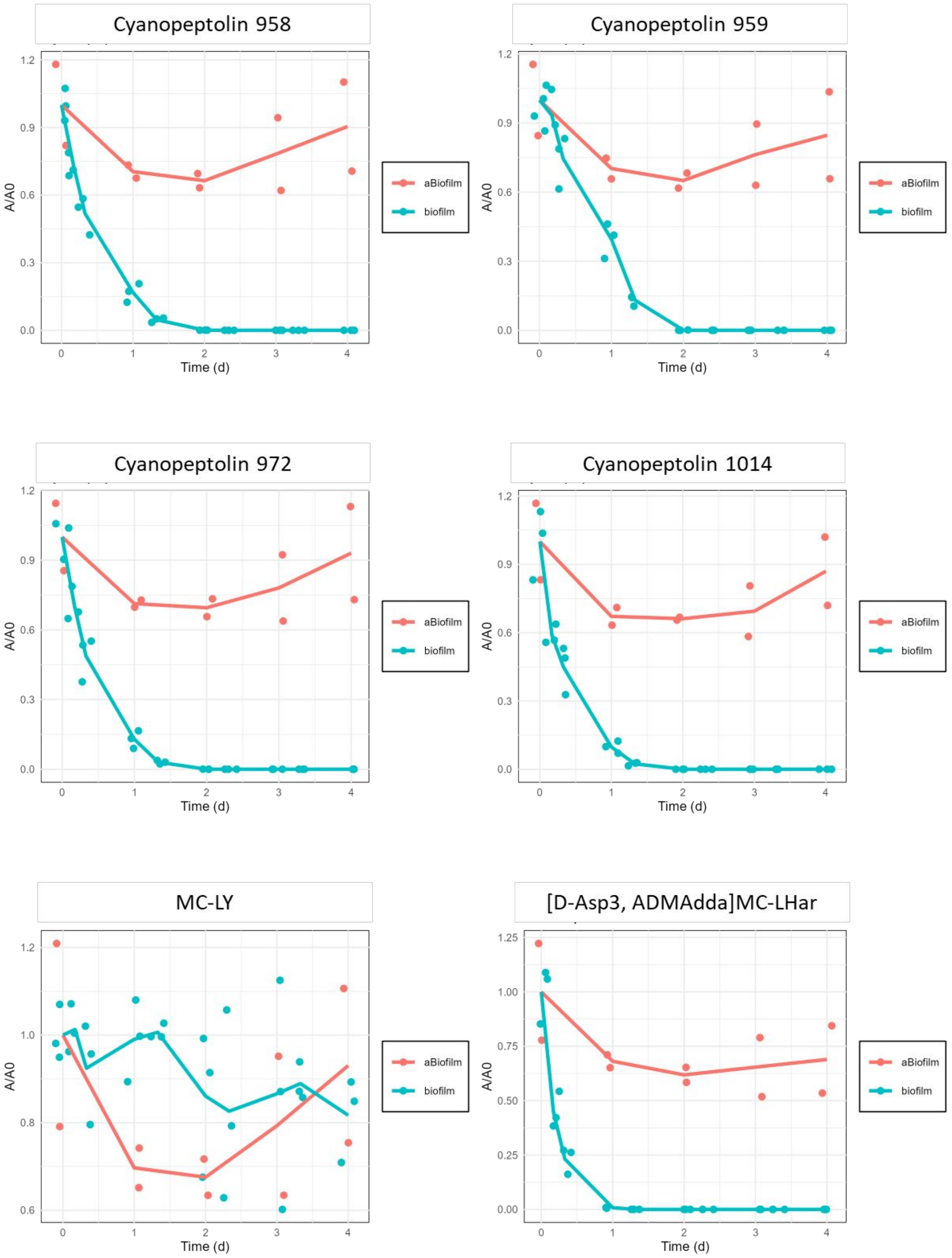

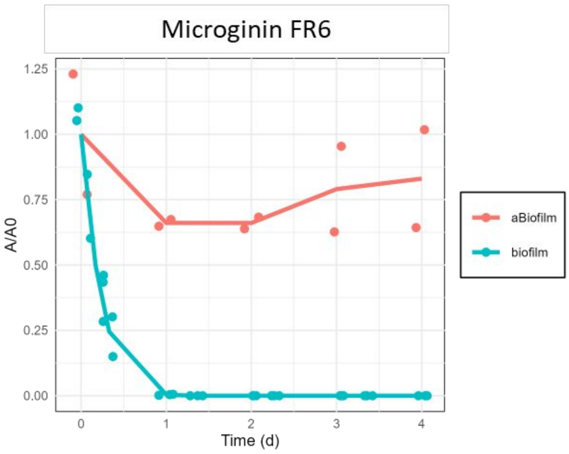
Microginin FR6
A/A0
1.25
1.00
0.75
0.50
0.25
0.00
0
1
2
3
4
Time (d)
aBiofilm
biofilm

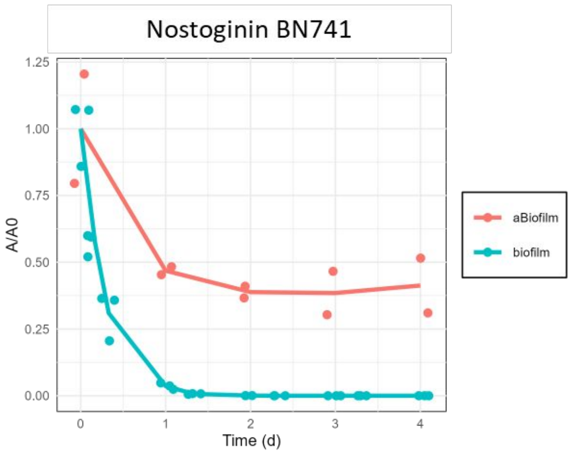
Nostoginin BN741
A/A0
1.25
1.00
0.75
0.50
0.25
0.00
0
1
2
3
4
Time (d)
aBiofilm
biofilm

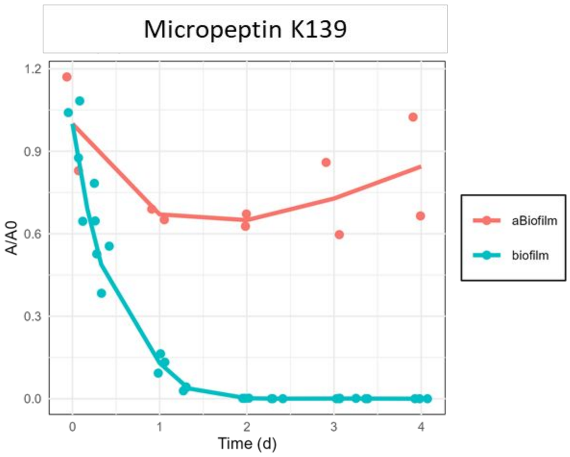
Micropeptin K139
A/A0
1.2
0.9
0.6
0.3
0.0
0
1
2
3
4
Time (d)
aBiofilm
biofilm

Figure S8. Biotransformation kinetics of 6 additional cyanobacterial metabolites, extracted form *Microcystis* G2011, *Microcystis* G2020 and *Planktothrix* G2020 exposed to Chriesbach biofilm suspensions and the respective abiotic controls (aBiofilm), showing the detected peak area normalized to the initial peak area across the incubation time. Note: due to a lack of experimental materials and low metabolite abundance in extract mixture, the result of the following metabolites in autoclaved biofilms was not determined: anabaenopeptin 807, anabaenopeptin D and anabaenopeptin J.

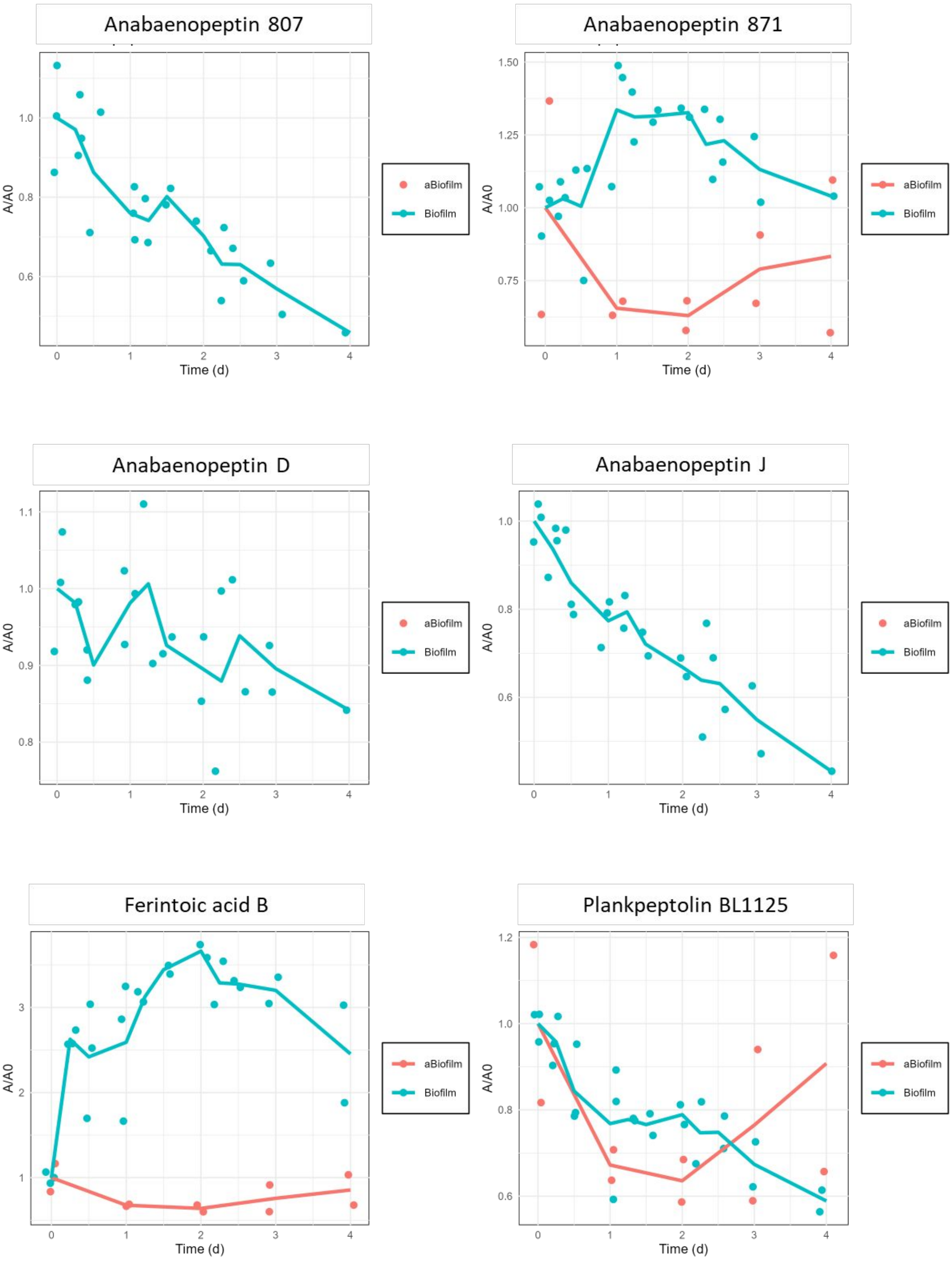

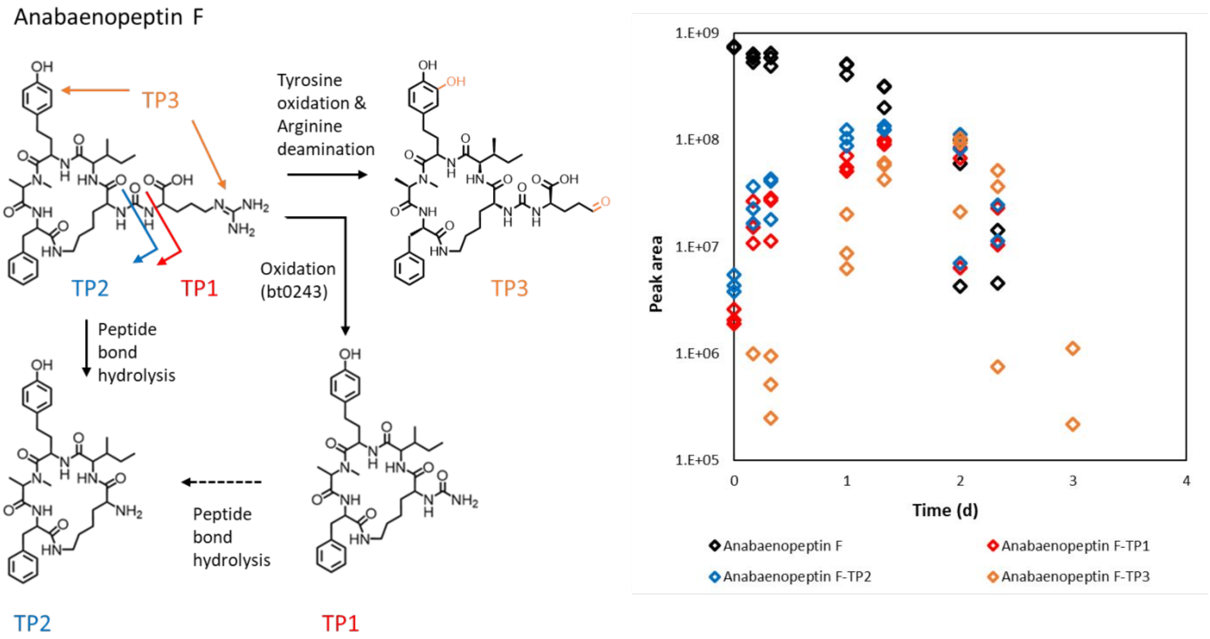

Figure S9. Kinetics of biotransformation products for Anabaenopeptin F, showing the peak area units of the parent (black) and transformation product TP1-3.

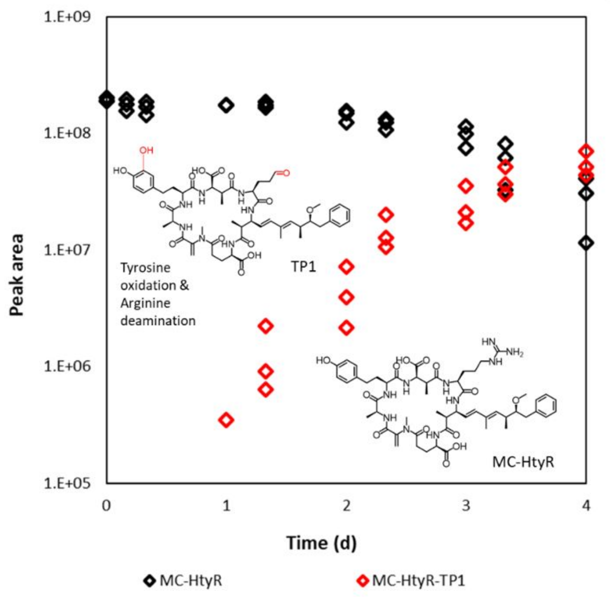

Figure S10. Kinetics of biotransformation products for MC-HtyR, showing the peak area units of the parent (black) and transformation product TP1 (red).

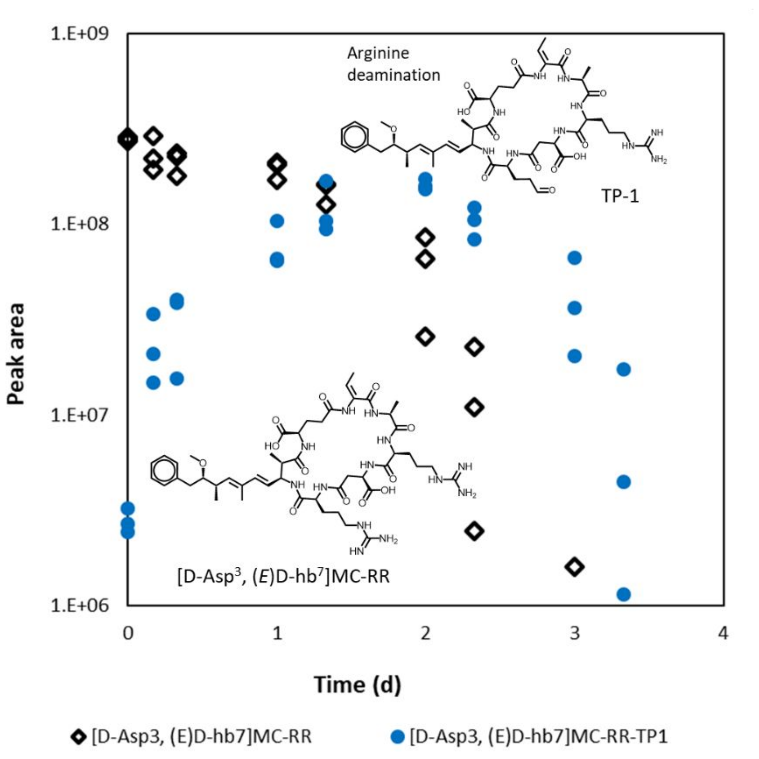

Figure S11. Kinetics of biotransformation products for [D-Asp^3, (*E*)D-hb^7]MC-RR, showing the peak area units of the parent (black) and transformation product TP1 (blue).
